# Bottom-Up Approach to Understand Chirality Transfer across Scales in Cellulose Assemblies

**DOI:** 10.1021/jacs.2c04522

**Published:** 2022-06-29

**Authors:** Giulio Fittolani, Denisa Vargová, Peter H. Seeberger, Yu Ogawa, Martina Delbianco

**Affiliations:** † Department of Biomolecular Systems, Max Planck Institute of Colloids and Interfaces, Am Mühlenberg 1, 14476 Potsdam, Germany; ‡ Department of Chemistry and Biochemistry, Freie Universität Berlin, Arnimallee 22, 14195 Berlin, Germany; § Univ. Grenoble Alpes, CNRS, CERMAV, 38000 Grenoble, France

## Abstract

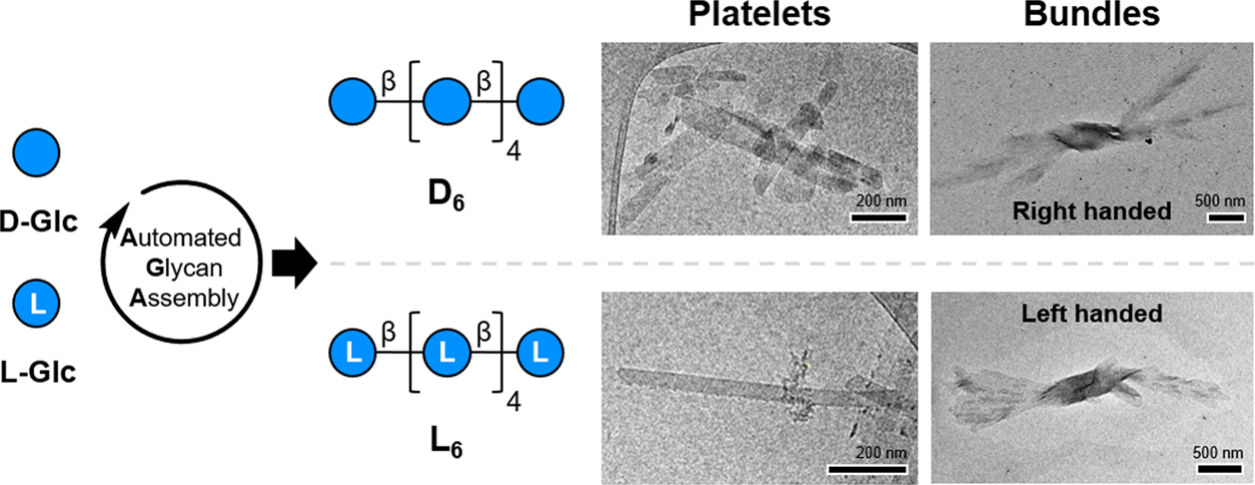

Cellulose is a polysaccharide that displays chirality across different scales, from the molecular to the supramolecular level. This feature has been exploited to generate chiral materials. To date, the mechanism of chirality transfer from the molecular level to higher-order assemblies has remained elusive, partially due to the heterogeneity of cellulose samples obtained *via* top-down approaches. Here, we present a bottom-up approach that uses well-defined cellulose oligomers as tools to understand the transfer of chirality from the single oligomer to supramolecular assemblies beyond the single cellulose crystal. Synthetic cellulose oligomers with defined sequences self-assembled into thin micrometer-sized platelets with controllable thicknesses. These platelets further assembled into bundles displaying intrinsic chiral features, directly correlated to the monosaccharide chirality. Altering the stereochemistry of the oligomer termini impacted the chirality of the self-assembled bundles and thus allowed for the manipulation of the cellulose assemblies at the molecular level. The molecular description of cellulose assemblies and their chirality will improve our ability to control and tune cellulose materials. The bottom-up approach could be expanded to other polysaccharides whose supramolecular chirality is less understood.

## Introduction

Chirality is a key feature of biopolymers and natural products.^1,2^ Molecular chirality based on covalent bonds is well understood, and synthetic methods provide control over asymmetric molecular construction.^3^ In contrast, the driving forces and key interactions imparting chirality at the supramolecular level are harder to elucidate and reproduce.^4^ Supramolecular chirality has been observed in inorganic materials, polymers, and biological aggregates (*e.g.*, amyloids), and much effort has been devoted to describing the origin of these helices.^5^ Synthetic models offered valuable tools to reveal key molecular features responsible for supramolecular chirality in more complex systems. For instance, well-defined model peptides have contributed to the long-lasting debate on the origin of the twist in amyloid aggregates.^6−8^ Synthetic peptides made of l- and d-amino acid sequences suggested that amino acids located at the termini were responsible for the twist adopted by peptide supramolecular fibers,^9,10^ with a left-handed twist associated with the presence of l-amino acids.^11,12^ Moreover, fine-tuning of these synthetic analogues generated novel, chiral self-assembled materials with controllable shapes and properties.^13−15^

The chirality of cellulose, the most abundant organic material on Earth, has not been dissected using a synthetic approach, despite the growing interest in the chiral properties of cellulose nanofibers (CNFs), cellulose nanocrystals (CNCs), and their assemblies.^16^ Native CNFs are intrinsically chiral, with nanoscale twists along the fiber direction. Chiral cellulose particles can assemble into larger chiral architectures *in vivo*. Helicoidal arrangements of cellulose fibrils are found in plant cell walls and are capable of generating colors in the absence of any pigment through structural coloration.^17,18^ Left-handed and, in some rare cases, right-handed helicoids have been observed.^17^ Still, the mechanism for helicoid formation in plants remains debated, invoking the role of other cell wall matrix constituents such as hemicelluloses.^18^

Inspired by nature, CNCs have been used to fabricate optical materials such as films^16,19,20^ and structurally colored pigments.^21^ Due to their chirality, CNCs have found applications as nanosized chiral inducers for liquid crystal assemblies^22^ and heterogeneous enantioselective palladium catalysis^23^ or as chiral templates.^24,25^*In vitro*, when a suspension of CNCs transitions from a diluted to a concentrated regime, spontaneous self-organization results in the formation of a chiral nematic liquid crystalline phase. Although the intrinsic twisted morphology of single CNCs is considered to govern the chirality of these chiral nematic phases, the mechanism by which nanoscale chirality is transferred to a larger scale is debated.^26−28^ While single particles (*i.e.*, CNCs) are right-handed, upon self-assembly, an inversion of chirality occurs, resulting in a left-handed chiral nematic phase.^29^ This inversion was quantified using atomic force microscopy (AFM) and electron diffraction methods.^30−32^ Recently, bundles of naturally sourced cellulose crystallites have been suggested to be responsible for the transfer of chirality across different hierarchical levels.^28,33^ However, heterogeneous degrees of polymerization and crystal sizes of cellulose nanomaterials obtained *via* top-down approaches complicated the description of these systems at the molecular level.^34^ Computational models have speculated on the origin of the twist in cellulose crystals and are, to date, the only option to study these systems at the molecular level.^35^ Thus, the molecular origin of this twist and the mechanism of chirality transfer to higher hierarchical assemblies remain open questions.

Herein, we exploit synthetic cellulose oligomers to understand the molecular bases of chirality transfer across scales. Using a bottom-up approach, we synthesized oligomers with well-defined sequences of natural d-glucose (d-Glc) and its enantiomer l-glucose (l-Glc) to manipulate the chirality at the sequence level. These cellulose oligomers self-assembled into platelets with controlled dimensions that further aggregated into bundles, displaying chiral features directly connected to their monosaccharide composition. The insertion of l-Glc units in the sequence of d-Glc cellulose oligomers drastically impacted the macroscopic properties, such as solubility, crystallinity, and chirality of the bundles. The bottom-up approach to study polysaccharide materials highlights the importance of the molecular sequence in dictating supramolecular assembly and chirality.

## Results and Discussion

### Cellulose Oligomers Assemble into Nanocrystals

**Figure 1:**
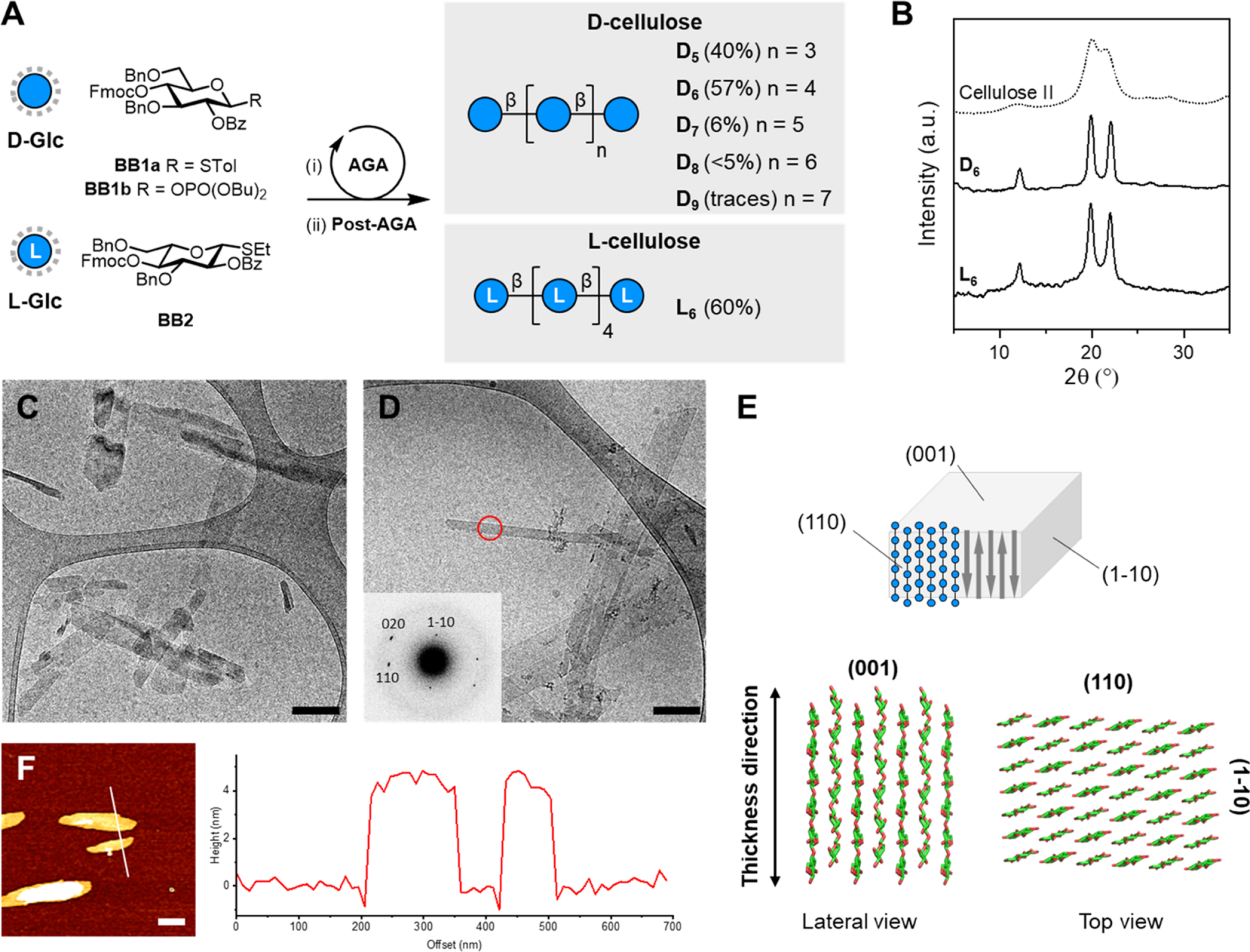
(A) AGA of cellulose analogues using protected monosaccharide BBs (overall yields reported in parentheses. Solid support and modules used for AGA and post-AGA are reported in the Supporting information). (B) Powder X-ray diffraction (XRD) profiles for selected cellulose oligomers indicating a cellulose II-type molecular packing. (C) Cryogenic transmission electron microscope (cryoTEM) image of **D_6_** platelets (scale bar 200 nm). (D) CryoTEM image of **L_6_** platelets with the electron diffraction diagram obtained from the red circled area (scale bar 200 nm). (E) Three-dimensional (3D) molecular model of the platelets composed of cellulose oligomers (**D_6_**) arranged in antiparallel fashion according to the cellulose II crystal structure. (F) AFM image of **L_6_** platelets (left) and height trace measurement (right) (scale bar 200 nm).

We targeted well-defined oligosaccharides resembling the macroscopic properties of cellulose (*i.e.*, crystallinity and mode of assembly) as a model system to study cellulose assembly. Cellulose oligomers with defined lengths (uniform dispersity, *Đ* = 1) were synthesized by automated glycan assembly (AGA)^36,37^ on a solid support (functionalized Merrifield resin; see the Supporting information) using protected d-Glc building blocks (BBs) **BB1a** and **BB1b** (Figure 1A). The BBs were equipped with an anomeric thioether or dibutylphosphate leaving group, while most hydroxyl groups were masked as benzyl (Bn) ethers and benzoyl (Bz) esters. Upon glycosylation, the cleavage of the 9-fluorenylmethoxycarbonyl (Fmoc) temporary protecting group liberated the hydroxyl group to be used in the following regioselective chain elongation. Iterative cycles of glycosylation and Fmoc deprotection permitted precise control over the length of the oligomer. Neighboring group participation of the ester group at C-2 ensured β-stereoselectivity during glycosylation. Each oligosaccharide was assembled overnight following previously reported conditions.^37^ Post-AGA manipulations included solid-phase methanolysis, photocleavage from the solid support, and hydrogenolysis (see the Supporting information). A single final purification step afforded the target cellulose analogues in overall yields of 6–60%. Longer cellulose analogues (degree of polymerization, DP > 6) were poorly soluble after the hydrogenolysis step and therefore obtained in a drastically decreasing isolated yield. The enantiomeric unnatural analogue **L**_**6**_ was assembled using protected l-Glc **BB2** (for the synthesis of **BB2**, see the Supporting information).

To evaluate the crystallinity and the aggregation tendency of these oligomers, we employed powder XRD, transmission electron microscopy (TEM), and AFM. Powder XRD indicated that all analogues **D**_**5**_, **D**_**6**_, and **D**_**7**_ (DP ranging from 5 to 7) assemble with the cellulose II crystal structure (Figures 1B and S3).^38^**D**_**5**_ was highly soluble, **D**_**6**_ showed intermediate solubility, while **D**_**7**_, **D**_**8**_, and **D**_**9**_ were poorly soluble in water (Table S1). The mirror-image oligomer **L**_**6**_ showed a cellulose II-type powder XRD profile and solubility similar to **D**_**6**_ (Figure 1B and Table S1).

Conventional TEM imaging of the negatively stained cellulose oligomers (1 mg/mL in water) indicated the presence of thin platelet-like particles for analogues with DP ≥ 6 (Figures S5, S9, S15, and S17). No platelets were observed for **D**_**5**_, suggesting that its high water solubility prevented the assembly at the concentration used for this study (1 mg/mL in water). The platelets obtained from **D**_**6**_ are *ca*. 50–500 nm long and *ca*. 20–50 nm wide. A similar morphology was observed for the assembly of enzymatically synthesized cellulose oligomers^39^ and for mercerized cellulose nanocrystals,^40^ both based on the cellulose II-type crystal structure. CryoTEM demonstrated that the platelets existed in aqueous suspension and were not the result of solvent evaporation (Figure 1C,D). Intra- and intermolecular hydrogen bonds between the hydroxyl groups and hydrophobic interactions between the C–H-rich faces of the glucopyranose rings stabilize the platelets through noncovalent interactions.^41^ Electron diffraction (ED) analysis of the platelets generated from **D**_**6**_ and **L**_**6**_ confirmed the cellulose II-type assembly (Figure 1D) and indicated a molecular packing where the cellulose chains are aligned in an antiparallel manner along the platelet thickness (Figure 1E). The (001) faces are exposed on the top and bottom sides of the platelets, presenting an alternation of reducing and nonreducing ends at the surface. The (110) face is exposed at the platelet tip and presents the Glc hydrophobic face. In aqueous media, the crystal growth along the hydrophobic [110] direction is faster than along the hydrophilic [1–10] direction, resulting in elongated platelets.^39^ Owing to the controlled length of the cellulose oligomers, the platelets have a well-defined thickness corresponding to the oligomer length. AFM analysis confirmed the tunable thickness of the platelets (Figure 1F). The increments of about 0.5 nm across the oligomer series are consistent with the addition of a single Glc unit (Figures S32–S36). This finding suggests that chemical synthesis can generate cellulose materials with tunable and controlled subnanometer dimensions.

### Nanocrystals Assemble into Chiral Bundles

**Figure 2:**
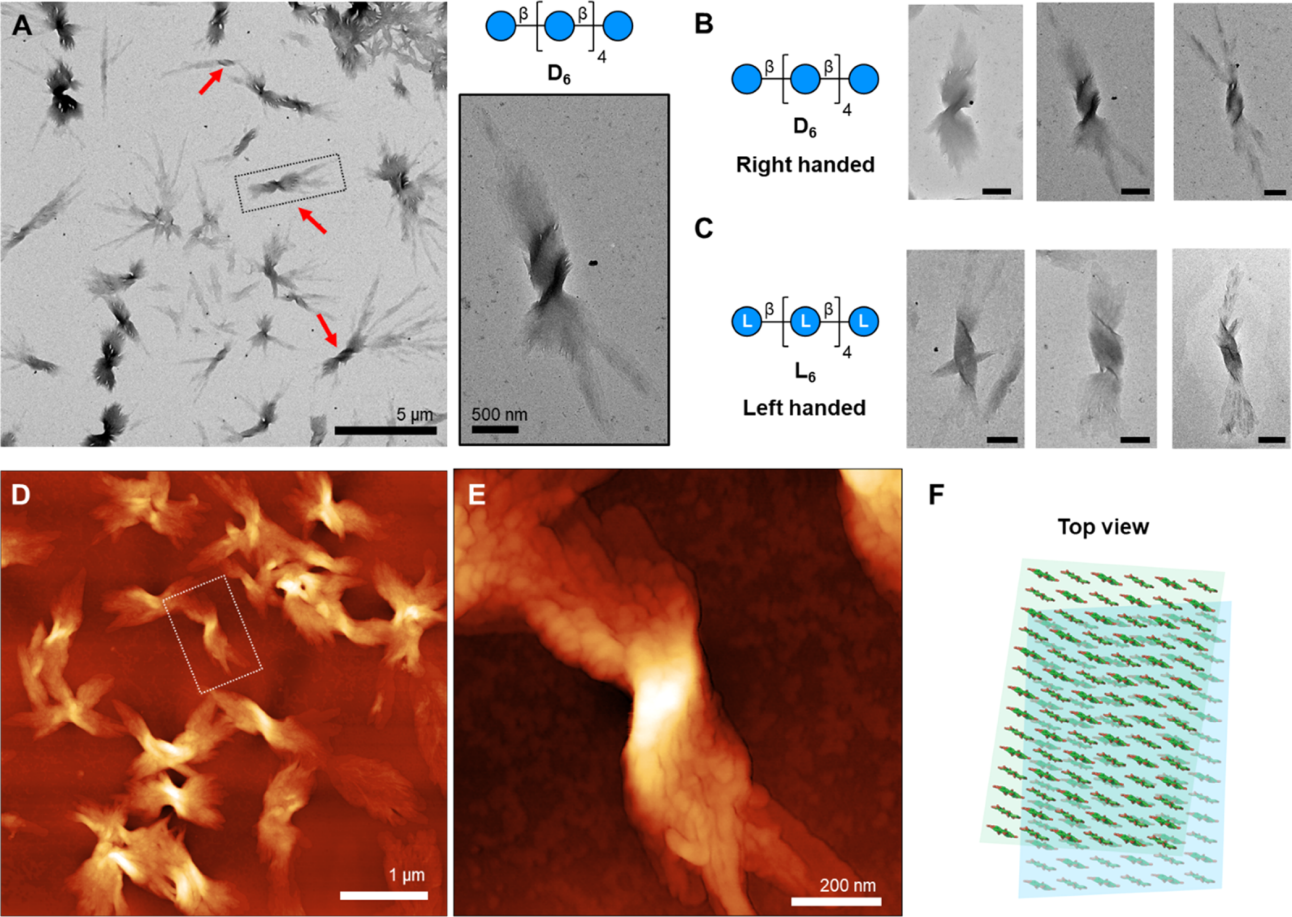
(A) TEM image of **D_6_** (1 mg/mL aqueous suspension) shows bundles of platelets with intrinsic chirality (red arrows). (B) TEM images of **D_6_** (1 mg/mL aqueous suspension) bundles showing an intrinsic right-handed chirality (scale bar 500 nm). (C) TEM images of **L_6_** (1 mg/mL aqueous suspension) bundles showing an intrinsic left-handed chirality (scale bar 500 nm). (D, E) AFM images of **D_6_** (1 mg/mL aqueous suspension) bundles showing an intrinsic right-handed chirality. AFM samples were prepared on TEM carbon-coated copper grids (see the Supporting information, Figure S37). (F) The fan-like arrangement of the stacking platelets was interpreted as a rotation between the (001) planes.

No evident chiral features were observed when inspecting the single platelets. However, TEM and AFM imaging revealed the formation of bundles of platelets alongside the isolated ones. These bundles displayed intrinsic chiral features clearly distinguishable by TEM (Figure 2A) and retained the cellulose II crystal structure (Figure S13). **D**_**6**_ bundles showed a right-handed twist (Figures 2B and S6–S8), while **L**_**6**_ bundles were left-handed (Figures 2C and S10–S12). The absolute chirality of the bundles was confirmed by AFM imaging (Figure 2D,E). Bundles were observed when preparing samples on different surfaces and confirmed by different imaging techniques (*i.e.*, SEM and AFM; Figure S39). Spherulite-type assemblies have been observed previously for enzymatically synthesized α-chitin^42^ and cellulose II^43^ aggregates, although no chiral features were recognized. The formation of bundles in our system reflected the strong tendency of the platelets to interact with each other during the drying process, likely due to the increasing concentration.

The analysis of samples at different drying conditions clarified the mechanism of the evaporation-induced assembly. Aqueous suspensions were deposited on TEM grids, and the suspension was blotted with filter paper to a different extent, leading to different amounts of water left on the grid surface at the end of the drying process. The sample prepared with extensive blotting showed mainly flat individual platelets, while short blotting, hence more remaining water, promoted the formation of large bundles (Figure S19). With decreasing blotting strength, multiple platelets stack on top of each other and a twist along the main axis appears (Figure 2E). When enough water remained after blotting, no edge features can be seen by TEM, suggesting that the platelets tend to merge (or fuse). In agreement with this observation, we found that slowing down the evaporation process favored the formation of a large number of bundles, while at fast drying times, the sample is mostly composed of platelets (Figure S20). Varying the concentration of the initial solution did not significantly affect the morphology of the bundles (Figure S21).

### Chirality Transfer from the Oligomers to the Bundles

During the evaporation-induced assembly, the platelets stack on top of each other and rotate in a fan-like manner, suggesting a slight rotation between the (001) crystal faces (Figures 2E and S14). Thus, we hypothesized that the (001) surface of the platelets (or the interaction between these surfaces) plays a major role in the assembly process, determining the chirality of the bundles (Figure 2F). This hypothesis is in agreement with amyloid-type assemblies of peptides, in which amino acids at the termini are responsible for the supramolecular twist.^9,10^ Chirality transfer processes from different parts of the monomeric units (core and/or termini) have also been reported for supramolecular polymer assemblies.^44−46^

**Figure 3:**
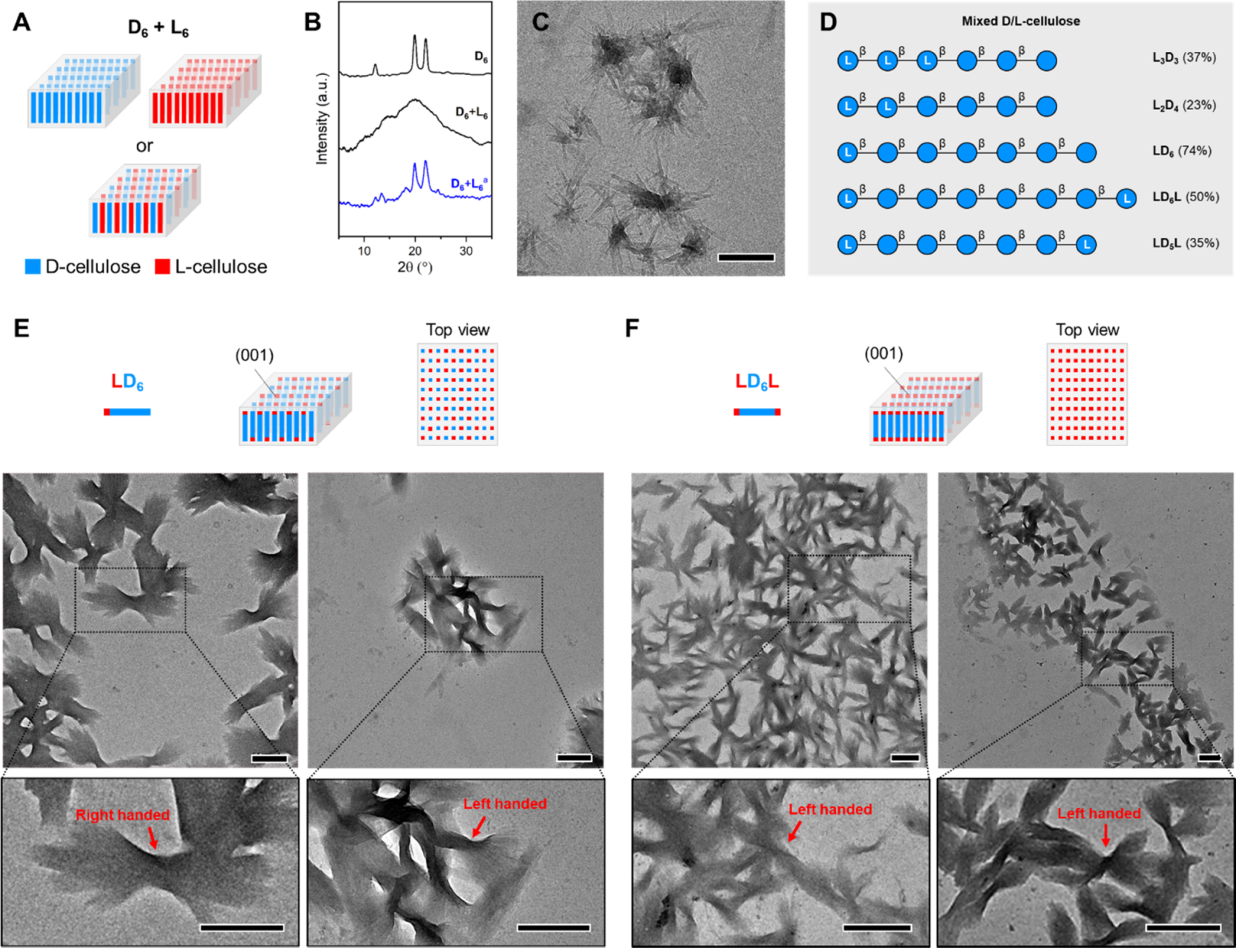
(A) Simplified model of platelets formed by mixing **D_6_** and **L_6_**. Self-sorting (top) or coassembly (bottom) of the two enantiomers are two possible scenarios. (B) Powder XRD profiles for **D_6_** + **L_6_** racemic mixtures. ^a^The sample was prepared by recrystallization from DMSO/MeOH (see the Supporting information). (C) TEM image of **D_6_** + **L_6_** (MeOH suspension) after recrystallization (scale bar, 500 nm). (D) Hybrid d-/l-cellulose analogues were synthesized using protected monosaccharide BBs (overall yields reported in parentheses). BBs, solid support, and modules used for AGA and post-AGA are reported in the Supporting information. (E) Simplified model of **LD_6_** platelets and top view of the (001) face (top). TEM images of **LD_6_** bundles (aqueous suspension, bottom) showing a twisted morphology (red arrows, scale bars 500 nm). (F) Simplified model of **LD_6_L** platelets and top view of the (001) face (top). TEM images of **LD_6_L** bundles (MeOH suspension, bottom) showing a twisted morphology (red arrows, scale bars 500 nm).

To gain insight into the origin of the twist, we designed three different experiments. First, we analyzed the assembly of a **D**_**6**_ + **L**_**6**_ racemic mixture (Figure 3A). A 1:1 mixture of **D**_**6**_ and **L**_**6**_ was dissolved in water and lyophilized; the XRD profile showed an amorphous arrangement (Figure 3B). No platelets or bundles were observed during TEM analysis of the **D**_**6**_ + **L**_**6**_ solution in water (1 mg/mL), suggesting an interaction between the two enantiomers that prevented crystallization. When the **D**_**6**_ + **L**_**6**_ racemic mixture was recrystallized using a DMSO to MeOH solvent switch method (see the Supporting information), the powder XRD showed a prevalence of the cellulose II pattern and platelets were regenerated, but no chiral features were observed (Figures 3B,C and S22). Mixing suspensions of **D**_**6**_ and **L**_**6**_ platelets in different ratios generated bundles with right- or left-handed chirality depending on the enantiomer in excess (Figure S23).

We then focused on the assembly of cellulose oligomers with a mixed sequence of d- and l-Glc residues. Five new oligomers were synthesized to selectively change the residues placed at the termini of the sequence (Figure 3D). In the second experiment, we analyzed the assembly of **L**_**2**_**D**_**4**_, **L**_**3**_**D**_**3**_, and **LD**_**6**_ in which a d-Glc core was capped with l-Glc residues. In this scenario, both d- and l-Glc residues are displayed at the (001) surface due to the antiparallel arrangement of cellulose II (Figure 3E, top). When a large portion of d-Glc residues was replaced by their mirror-image l-Glc, such as in **L**_**2**_**D**_**4**_ and **L**_**3**_**D**_**3**_, crystallinity was lost and solubility drastically increased (Table S1 and Figures S3 and S4). In contrast, when only one l-Glc unit was placed at the nonreducing end of the oligomer, such as in **LD**_**6**_, the solubility was comparable to that of **D**_**6**_ and the cellulose II crystal packing was preserved (Table S1 and Figure S3). TEM analysis showed **LD**_**6**_ platelets and bundles with similar sizes and shapes as for the model system **D**_**6**_ (Figures S24 and S25). However, the introduction of l-Glc weakened the twisting tendency and no clear handedness could be deduced from TEM images (Figures 3E, bottom, and S25).

In a third experiment, we focused on oligomers bearing l-Glc residues at both termini, such as in **LD**_**5**_**L** and **LD**_**6**_**L**. In this scenario, only l-Glc residues are displayed at the (001) surface, while the core of the crystal is based on d-Glc units (Figure 3F, top). The solubility of these oligomers was comparable to the model system **D**_**6**_ (Table S1); however, unexpectedly, **LD**_**5**_**L** and **LD**_**6**_**L** displayed a cellulose IV_II_-type XRD profile^47^ (Figure S3) and assembled into square platelets with no bundle-type aggregates (Figures S26 and S30). Recrystallization using a solvent switch method from DMSO to MeOH converted **LD**_**6**_**L** to cellulose II, as confirmed by XRD (Figure S4), and generated the typical platelet morphology (Figures S27 and S28). Similar to the second scenario, **LD**_**6**_**L** bundles of platelets displayed attenuated chiral features and, in some cases, the bundles appeared to be left-handed (Figure 3F, bottom, and S29).

Overall, the data on **LD**_**6**_ and **LD**_**6**_**L** suggest that perturbing the molecular nature of the surface of cellulose II platelets drastically affects the chirality of their assembly. Bundles with much less pronounced chiral features were observed. No complete inversion of bundle chirality was noticed in the case of **LD**_**6**_**L**, suggesting that not only the surface but also the core of the platelets are responsible for inducing the chirality of the homochiral assemblies (*i.e.*, **D**_**6**_).

## Conclusions

We established a model system for the study of cellulose aggregation using synthetic oligomers produced by AGA. Cellulose oligomers of well-defined lengths and sequences, including both d- and l-Glc, self-assembled into thin platelets with thickness matching the length of a single oligomer chain and arranged in a cellulose II fashion (antiparallel chain arrangement). The thickness of the platelet can be manipulated at the nanoscale by tuning the length of the synthetic cellulose oligomer. The synthetic cellulose platelets further assembled into bundles displaying intrinsic chiral features directly connected to the chirality of the cellulose chain (right-handed for **D**_**6**_ and left-handed for **L**_**6**_). Synthetic hybrid d- and l-cellulose oligomers helped to elucidate the origin of the chirality of these bundles. Terminal residues of opposite chirality drastically weakened the twisting tendency of the bundles, suggesting that the surface of the platelets plays an important role in determining the chirality of the bundles. Still, the surface does not solely determine the twist, as complete inversion of chirality was not observed for analogues displaying only l-Glc at the extremities.

Bundles of naturally sourced cellulose crystallites have been recently proposed to be a key element in the transfer of chirality across different hierarchical levels.^28,33^ Our model system offers a well-defined approach to validate this hypothesis and could be extended to other biopolymers, such as chitin, a polysaccharide composed of β-1,4-linked *N*-acetyl glucosamine. In contrast to cellulose, the chiral nature of chitin nanocrystals (ChNCs) remains elusive^32^ even though the *in vitro* formation of chiral nematic phases^48^ and helicoidal organization of chitin microfibrils have been reported.

## Supporting Information Available

All experimental details regarding building block synthesis, AGA, XRD, TEM, AFM, and SEM are reported in the Supporting information (PDF)
