# Supplementary material for: Bottom-Up Approach to Understand Chirality Transfer across Scales in Cellulose Assemblies
Source: J Am Chem Soc. 2022 Jun 29;144(27):12469–75. doi: 10.1021/jacs.2c04522 (PMC9284553; doi:10.1021/jacs.2c04522)
Supplement: Supplementary file 1 — ja2c04522_si_001.pdf [file ja2c04522_si_001.pdf]

Supporting Information

Bottom-up Approach to Understand Chirality Transfer across Scales in Cellulose Assemblies

Giulio Fittolani,[1,2] Denisa Vargová,[1] Peter H. Seeberger, [1,2] Yu Ogawa,[3*] and Martina Delbianco[1*]

[1]Department of Biomolecular Systems, Max Planck Institute of Colloids and Interfaces, Am Mühlenberg 1, 14476 Potsdam, Germany

[2]Department of Chemistry and Biochemistry, Freie Universität Berlin, Arnimallee 22, 14195 Berlin, Germany

[3]Univ. Grenoble Alpes, CNRS, CERMAV, 38000 Grenoble, France

Table of Contents

1 General Materials and Methods **4**

2 Synthesis of Building Blocks **5**

3 Automated Glycan Assembly **18**

3.1 General materials and methods 18

3.2 Preparation of stock solutions 18

3.3 Modules for automated synthesis 18

3.4 Post-AGA manipulations 21

3.5 Oligosaccharides synthesis 23

3.5.1 D_5 24

3.5.2 D_6 27

3.5.3 D_7 29

3.5.4 D_8 32

3.5.5 D_9 36

3.5.6 L_6 40

3.5.7 L_3D_3 43

3.5.8 L_2D_4 46

3.5.9 LD_6L 49

3.5.10 LD_5L 52

3.5.11 LD_6 55

4 Oligosaccharides self-assembly **58**

4.1 Solubility measurement 58

4.2 Recrystallization 58

1.1. XRD analysis 58

4.3 TEM imaging 60

4.3.1 D_6 60

4.3.2 L_6 63

4.3.3 D_7 67

4.3.4 D_8 67

4.3.5 Mechanism of formation of twisted bundles 69

4.3.6 D_6+L_6 70

4.3.7 LD_6 71

4.3.8 LD_6L 73

4.3.9 LD_5L 76

4.4 AFM and SEM imaging 77
4.5 Chirality analysis 80
5 References 81

1 General Materials and Methods

All chemicals used were reagent grade and used as supplied unless otherwise noted. The automated syntheses were performed on a home-built synthesizer developed at the Max Planck Institute of Colloids and Interfaces.[1] Analytical thin-layer chromatography (TLC) was performed on Merck silica gel 60 F254 plates (0.25 mm). Compounds were visualized by UV irradiation or dipping the plate in a staining solution (sugar stain: 10% H_2SO_4 in EtOH; CAM: 48 g/L ammonium molybdate, 60 g/L ceric ammonium molybdate in 6% H_2SO_4 aqueous solution). Flash column chromatography was carried out by using forced flow of the indicated solvent on Fluka Kieselgel 60 M (0.04 – 0.063 mm). Analysis and purification was performed by reverse phase HPLC was performed by using an Agilent 1200 series. Products were lyophilized using a Christ Alpha 2-4 LD plus freeze dryer. ^{1}H, ^{13}C and HSQC NMR spectra were recorded on a Varian 400-MR (400 MHz), Varian 600-MR (600 MHz), or Bruker Biospin AVANCE700 (700 MHz) spectrometer. Spectra were recorded in $CDCl_3$ by using the solvent residual peak chemical shift as the internal standard ($CDCl_3$: 7.26 ppm ^{1}H, 77.0 ppm ^{13}C) or in D_2O using the solvent as the internal standard in ^{1}H NMR (D_2O: 4.79 ppm ^{1}H). ^{1}H NMR integrals of the resonances corresponding to residues at the reducing end are reported as non-integer numbers and the sum of the integrals of α and β anomers is set to 1. High resolution mass spectra were obtained using a 6210 ESI-TOF mass spectrometer (Agilent) and a MALDI-TOF autoflex™ (Bruker). MALDI and ESI mass spectra were run on IonSpec Ultima instruments. IR spectra were recorded on a Perkin-Elmer 1600 FTIR spectrometer. Optical rotations were measured by using a Perkin-Elmer 241 and Unipol L1000 polarimeter.

2 Synthesis of Building Blocks

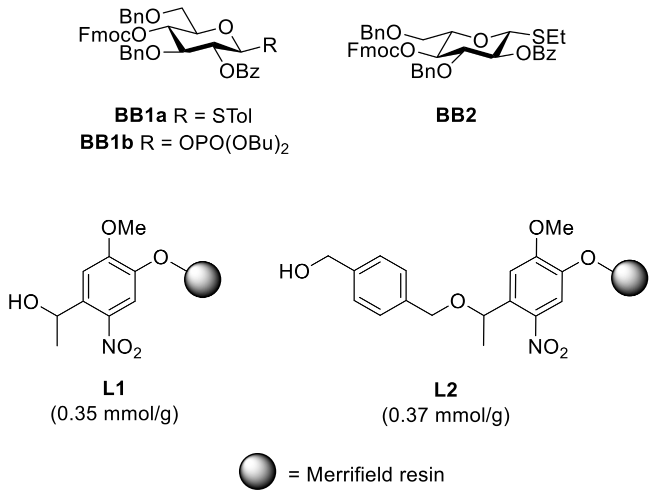

Figure S1 BBs and solid supports used in this work.

Building block **BB1a** was purchased from GlycoUniverse (Germany). Building block **BB1b** was synthesized according to previously reported procedures.[2] Merrifield resin equipped with a photocleavable linker (**L1**, loading 0.35 mmol/g) was prepared according to previous literature.[3] The synthesis of **BB2** is reported herein (Scheme S1).

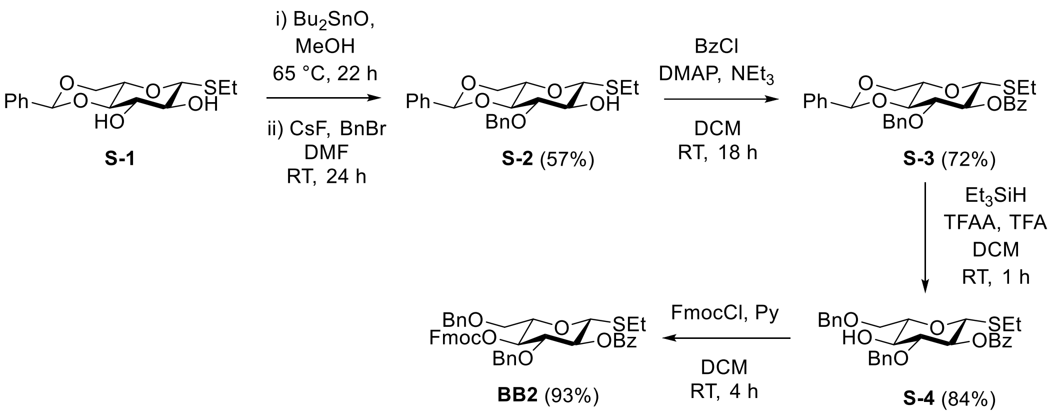

Scheme S1 Synthetic route to **BB2**.

Synthesis of **S-2**

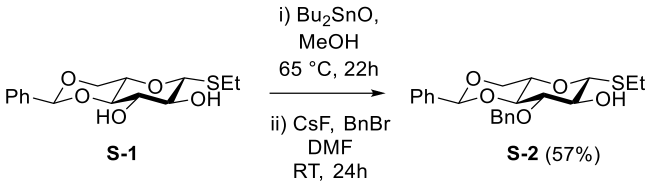

S-1 was synthesized according to previously reported procedures.[4]

S-1 (1.06 g, 3.40 mmol) was dissolved in MeOH (40 mL), di-*n*-butyltin oxide (Bu_2SnO) (1.02 g, 4.10 mmol) was added and the reaction mixture (white suspension) heated at reflux (65 °C) under vigorous stirring for 22 h. The reaction mixture (clear solution) was then cooled, concentrated under reduced pressure and the crude product was used in the next step without further purification. The crude was dissolved in DMF (20 mL). Benzyl bromide (0.49 mL, 4.13 mmol) and cesium (I) fluoride (CsF) (670 g, 4.41 mmol) were added and the clear solution stirred at RT for 24 h under Ar atmosphere. The cloudy reaction mixture was diluted with EtOAc and the organic layer was passed through a short plug of silica gel and concentrated under reduced pressure. The crude product was diluted with EtOAc and the organic layer washed once with an aqueous solution of KF (1 M), once with water, once with brine, dried over Na_2SO_4, and concentrated under reduced pressure. The crude product was purified by silica gel flash column chromatography (Hexane : Acetone = 3:1→2:1→1:1) to yield **S-2** as a white solid (0.78 g, 57%).

^{1}H NMR (400 MHz, Chloroform-*d*) δ 7.52 – 7.46 (m, 2H), 7.43 – 7.27 (m, 8H), 5.58 (s, 1H), 4.98 (d, *J* = 11.6 Hz, 1H), 4.82 (d, *J* = 11.6 Hz, 1H), 4.47 (d, *J* = 9.6 Hz, 1H), 4.36 (dd, *J* = 10.5, 5.0 Hz, 1H), 3.79 (t, *J* = 10.3 Hz, 1H), 3.75 – 3.64 (m, 2H), 3.58 (ddd, *J* = 9.9, 8.1, 1.9 Hz, 1H), 3.50 (ddd, *J* = 10.0, 8.7, 4.9 Hz, 1H), 2.75 (qd, *J* = 7.4, 2.8 Hz, 2H), 2.53 (d, *J* = 2.0 Hz, 1H), 1.32 (t, *J* = 7.4 Hz, 3H). ^{13}C NMR (101 MHz, Chloroform-*d*) δ 138.38, 137.30, 129.16, 128.62, 128.42, 128.21, 128.01, 126.13, 101.38, 86.69, 81.65, 81.35, 74.87, 73.09, 70.88, 68.77, 24.73, 15.38. $[\alpha]_D^{20}$ +39.51 (c 0.63 g/100 mL, $CHCl_3$). IR ν = 3362, 1367, 1088, 1063, 1009, 746, 697 cm^{-1}. R_f = 0.31 (Hexane : EtOAc 3:1). (ESI-HRMS) m/z 425.139 $[M+Na]^+$ ($C_{22}H_{26}O_5SNa$ requires 425.139).

^{1}H NMR of S-2 (400 MHz, $CDCl_3$)

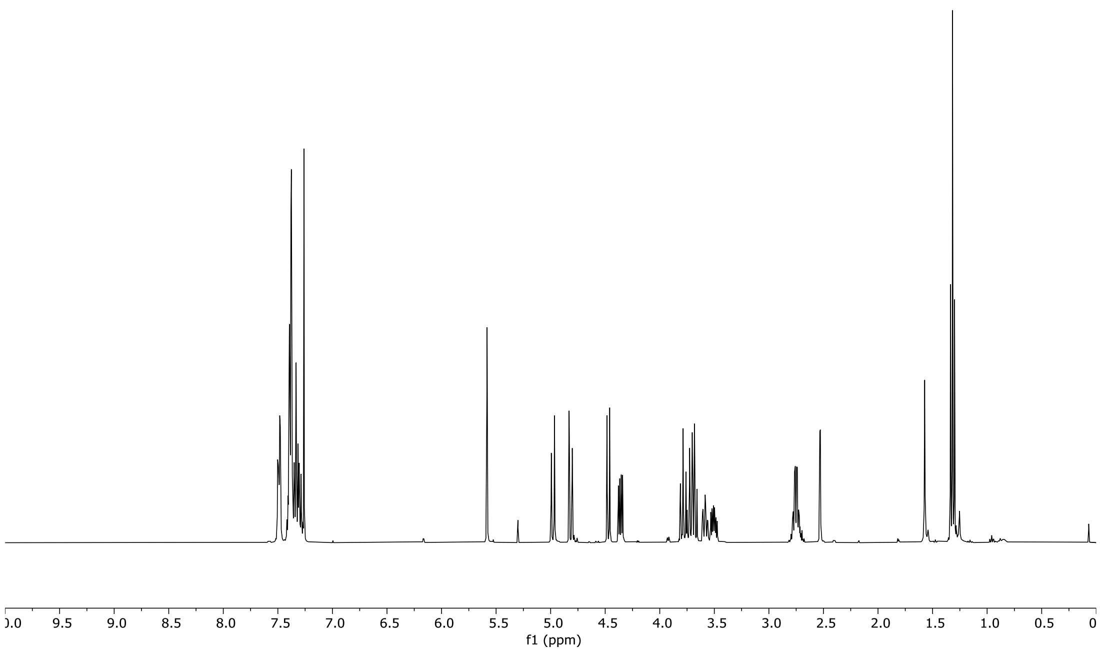

^{13}C NMR of S-2 (101 MHz, $CDCl_3$)

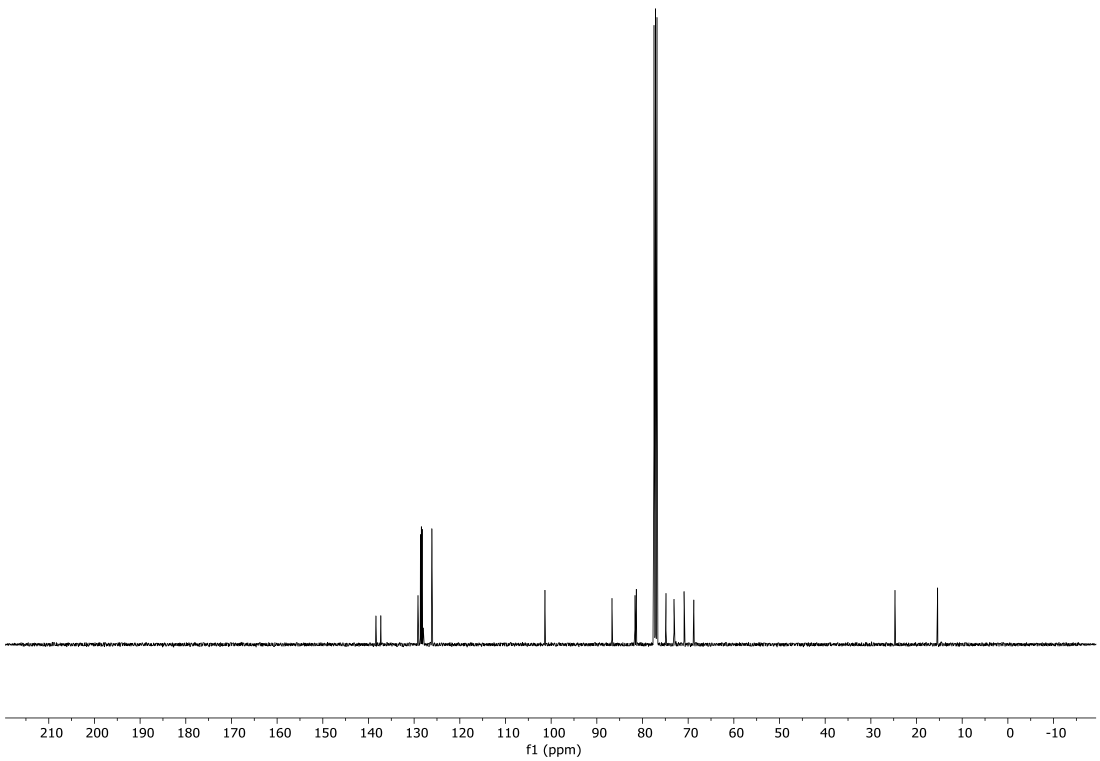

HSQC NMR of S-2 ($CDCl_3$)

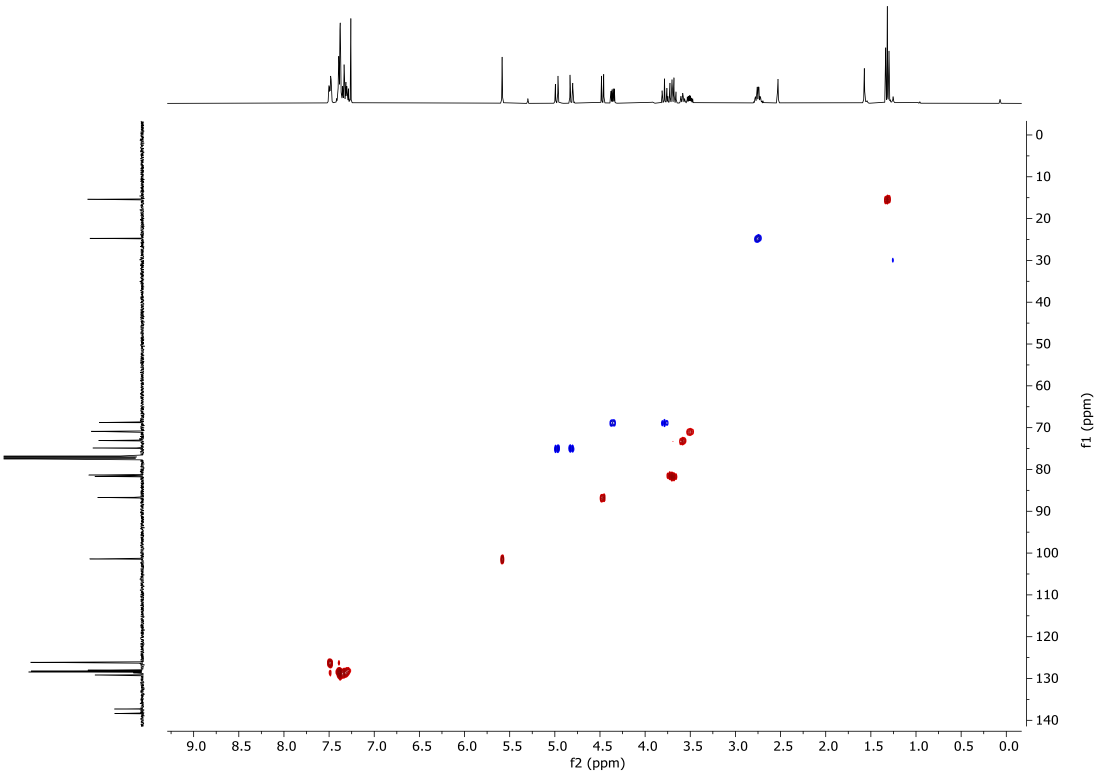

COSY NMR of S-2 ($CDCl_3$)

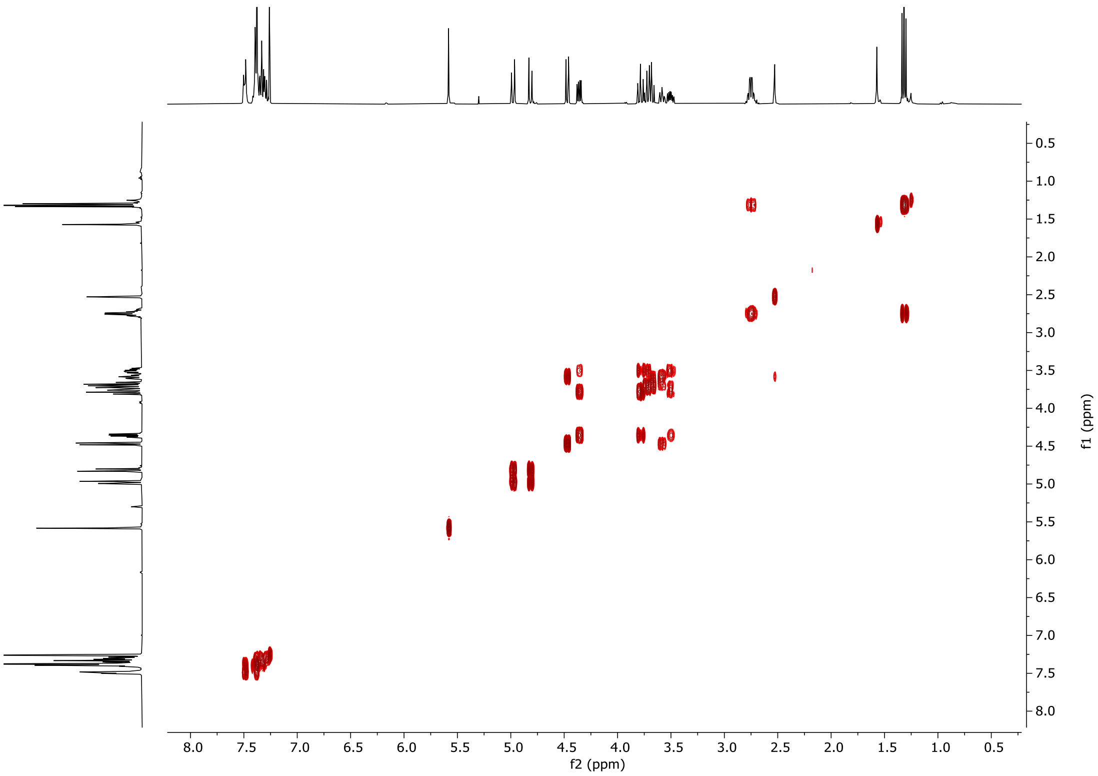

Synthesis of **S-3**

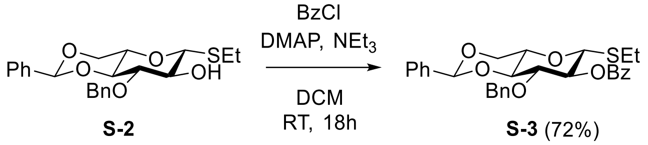

S-2 (775 mg, 1.93 mmol) was dissolved in anhydrous DCM (40 mL) under Ar atmosphere. Triethylamine (NEt_3) (2.86 mL, 0.58 mmol) and 4-dimethylaminopyridine (DMAP) (70 mg, 0.58 mmol) were added to the solution, while stirring. Benzoyl chloride (BzCl) (350 µL, 3.03 mmol) was slowly added at 0°C and the reaction allowed to RT. After 18 h the reaction was diluted with DCM and quenched with a saturated aqueous solution of $NaHCO_3$. The organic layer was washed three times with a saturated aqueous solution of $NaHCO_3$ and once with brine. The organic layer was dried over Na_2SO_4 and concentrated under reduced pressure. The crude product was purified through a short plug of silica (EtOAc isocratic) and recrystallized from Hexane : EtOAc to yield **S-3** as a white solid (708 mg, 72%).

^{1}H NMR (400 MHz, Chloroform-*d*) δ 8.06 – 7.96 (m, 2H), 7.66 – 7.33 (m, 8H), 7.19 – 7.04 (m, 5H), 5.63 (s, 1H), 5.35 (dd, *J* = 10.0, 8.3 Hz, 1H), 4.84 (d, *J* = 11.9 Hz, 1H), 4.71 (d, *J* = 11.9 Hz, 1H), 4.63 (d, *J* = 10.1 Hz, 1H), 4.42 (dd, *J* = 10.5, 5.0 Hz, 1H), 3.96 – 3.79 (m, 3H), 3.57 (ddd, *J* = 10.0, 8.8, 4.9 Hz, 1H), 2.73 (qd, *J* = 7.5, 3.6 Hz, 2H), 1.23 (t, *J* = 7.5 Hz, 3H). ^{13}C NMR (101 MHz, Chloroform-*d*) δ 165.29, 137.88, 137.30, 133.33, 130.05, 129.85, 129.18, 128.50, 128.42, 128.29, 128.18, 127.70, 126.13, 101.39, 84.42, 81.79, 79.31, 74.34, 71.97, 70.84, 68.77, 24.15, 14.94. $[\alpha]_D^{20}$ -16.49 (c 1.77 g/100 mL, $CHCl_3$). IR ν = 1728, 1269, 1094, 1070, 699 cm^{-1}. R_f = 0.47 (Hexane : EtOAc = 3:1). (ESI-HRMS) m/z 529.166 [M+Na]$^+$ ($C_{29}H_{30}O_6SNa$ requires 529.166).

^{1}H NMR of S-3 (400 MHz, CDCl$_3$)

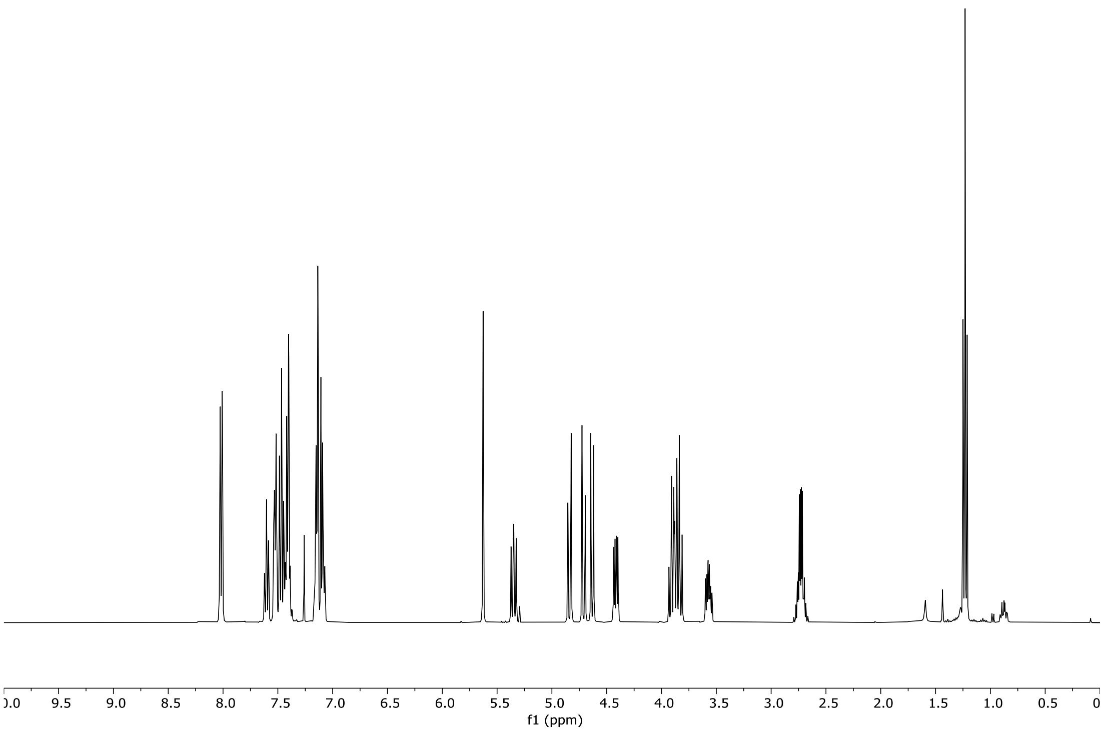

^{13}C NMR of S-3 (101 MHz, CDCl$_3$)

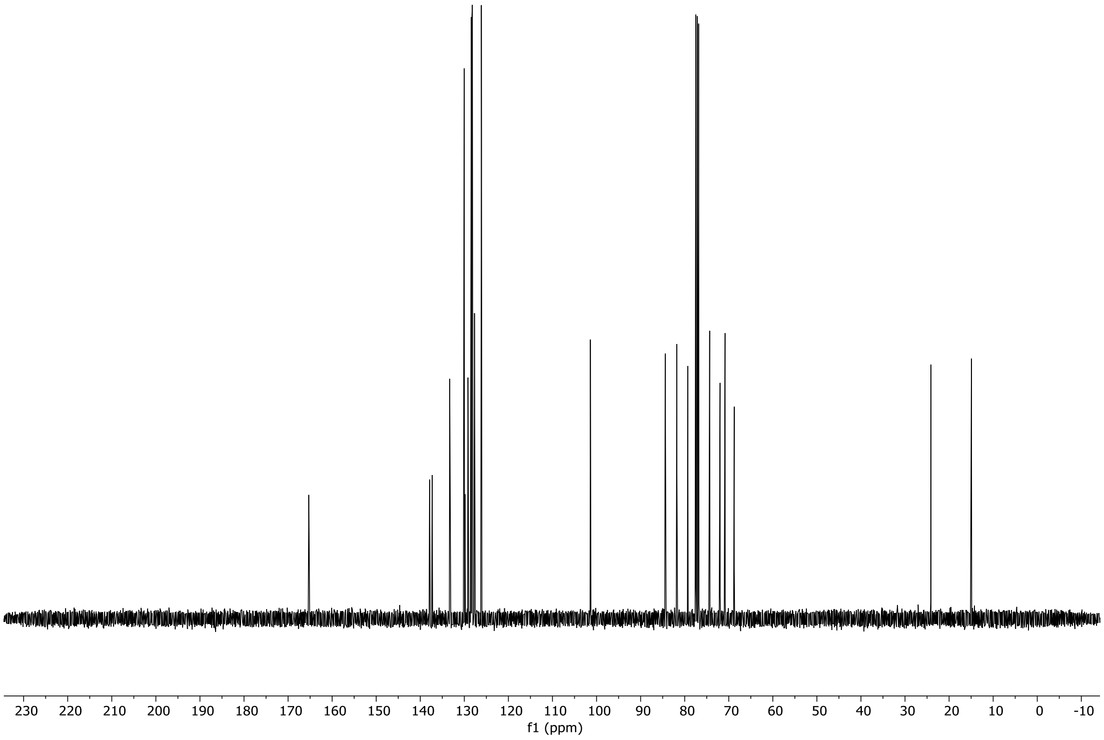

HSQC NMR of S-3 ($CDCl_3$)

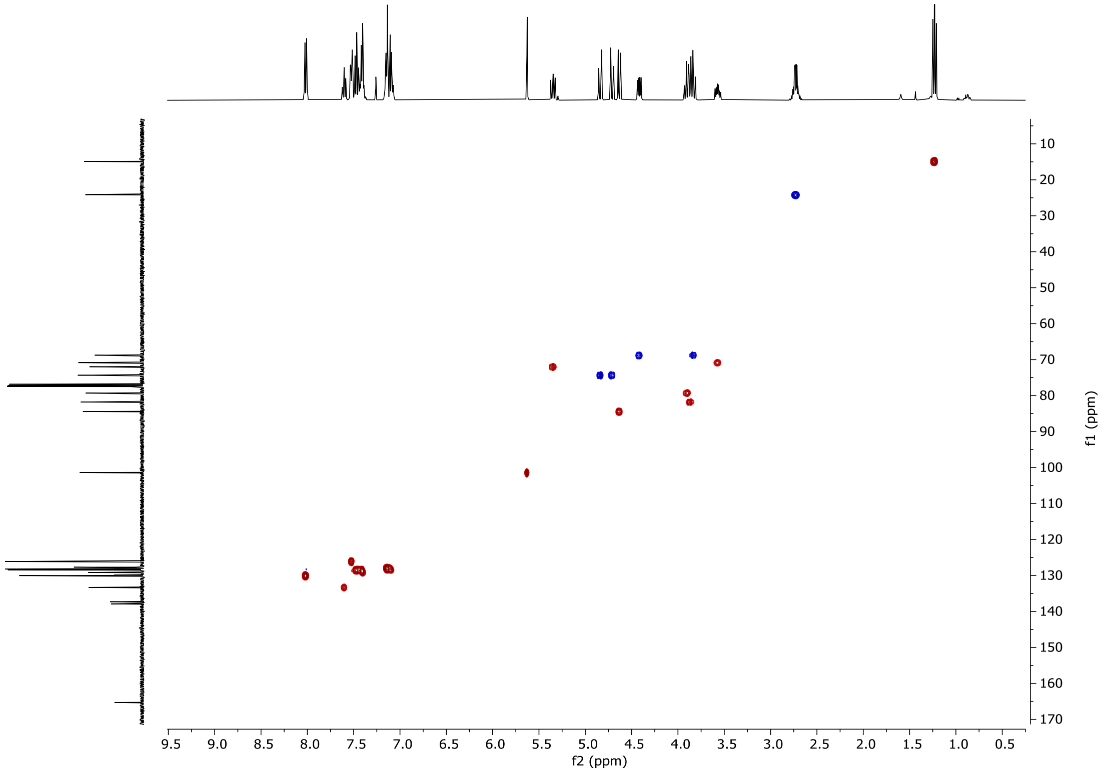

COSY NMR of S-3 ($CDCl_3$)

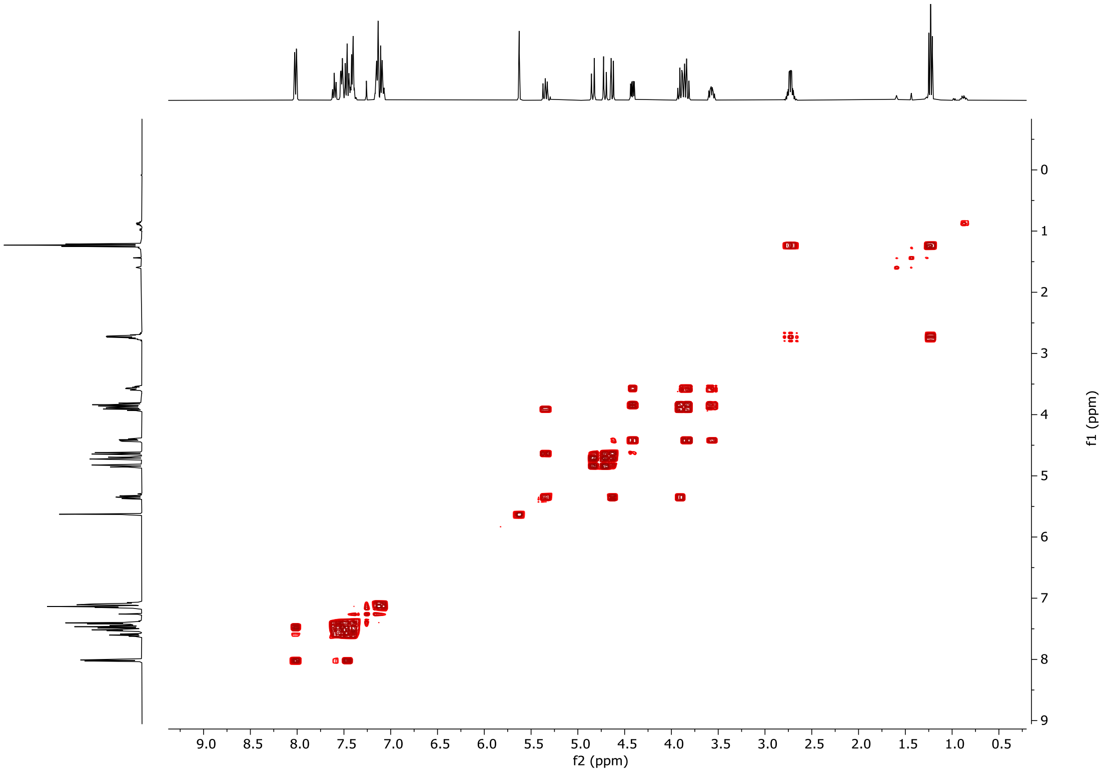

Synthesis of **S-4**

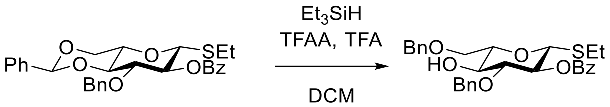

S-3 → **S-4** (84%)

S-3 (914 mg, 1.81 mmol) was dissolved in anhydrous DCM (30 mL) under Ar atmosphere. Triethylsilane (Et_3SiH) (1.72 mL, 10.8 mmol) and trifluoroacetic anhydride (TFAA) (250 µL, 1.80 mmol) were sequentially added to the stirred solution at 0°C. After 20 min, trifluoroacetic acid (TFA) (830 µL, 11.2 mmol) was added dropwise at 0°C. The reaction was allowed to RT and after 1 h quenched with a saturated aqueous solution of $NaHCO_3$. The organic layer was washed twice with a saturated aqueous solution of $NaHCO_3$, and once with brine. The crude product was purified by silica gel flash column chromatography (Hexane : EtOAc = 5:1→2:1) to give **S-4** as a white solid (0.77 g, 84%).

^{1}H NMR (600 MHz, Chloroform-*d*) δ 8.08 – 8.02 (m, 2H), 7.58 (ddt, *J* = 8.6, 7.2, 1.3 Hz, 1H), 7.50 – 7.43 (m, 2H), 7.39 – 7.28 (m, 5H), 7.18 (s, 5H), 5.28 (dd, *J* = 10.0, 9.1 Hz, 1H), 4.73 (d, *J* = 11.5 Hz, 1H), 4.69 (d, *J* = 11.5 Hz, 1H), 4.62 (d, *J* = 12.0 Hz, 1H), 4.58 (d, *J* = 12.0 Hz, 1H), 4.56 (d, *J* = 10.0 Hz, 1H), 3.85 – 3.75 (m, 4H), 3.70 (t, *J* = 9.0 Hz, 1H), 3.57 (dt, *J* = 9.6, 4.8 Hz, 1H), 2.76 – 2.63 (m, 2H), 1.23 (t, *J* = 7.4 Hz, 3H). ^{13}C NMR (151 MHz, Chloroform-*d*) δ 165.41, 138.08, 137.84, 133.33, 130.00, 128.63, 128.56, 128.54, 128.13, 128.00, 127.93, 127.91, 83.78, 83.68, 78.31, 74.80, 73.92, 72.48, 72.15, 70.69, 24.10, 15.02. $[\alpha]_D^{20}$ -3.03 (c 1.45 g/100 mL, $CHCl_3$). IR ν = 3479, 1725, 1269, 1068, 710, 698 cm^{-1}. R_f = 0.39 (Hexane : EtOAc = 3:1). (ESI-HRMS) m/z 531.181 $[M+Na]^+$ ($C_{29}H_{32}O_6SNa$ requires 531.181).

^{1}H NMR of S-4 (600 MHz, $CDCl_3$)

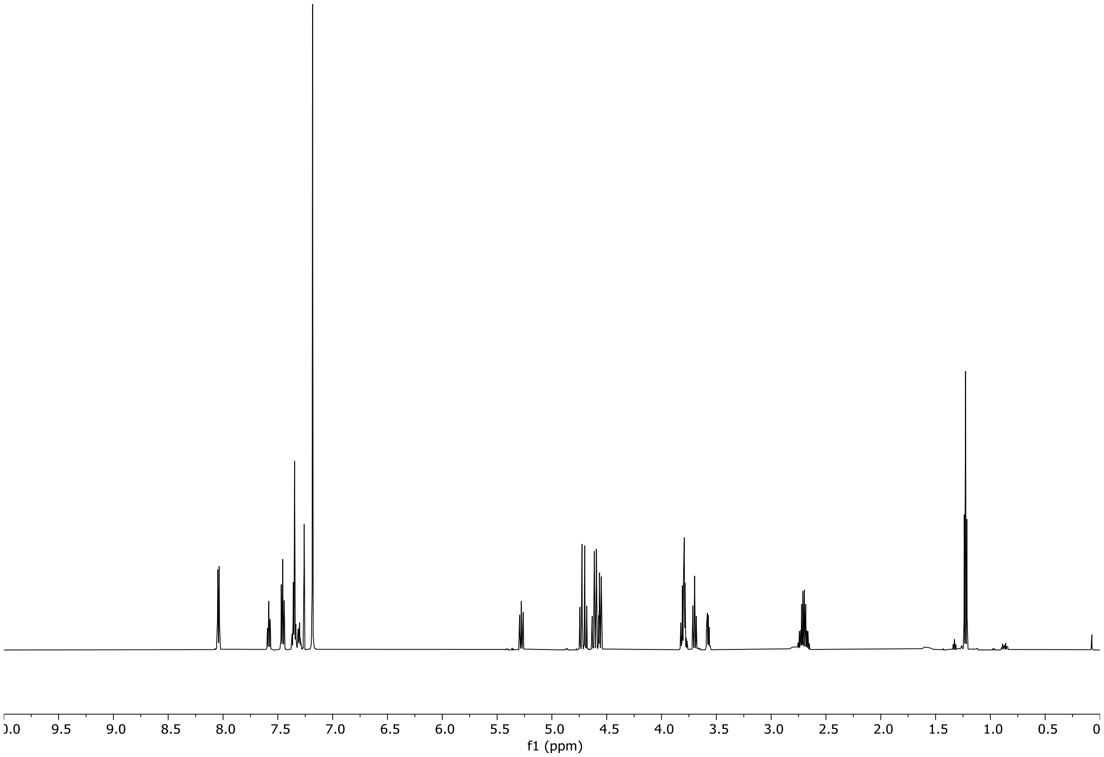

^{13}C NMR of S-4 (151 MHz, $CDCl_3$)

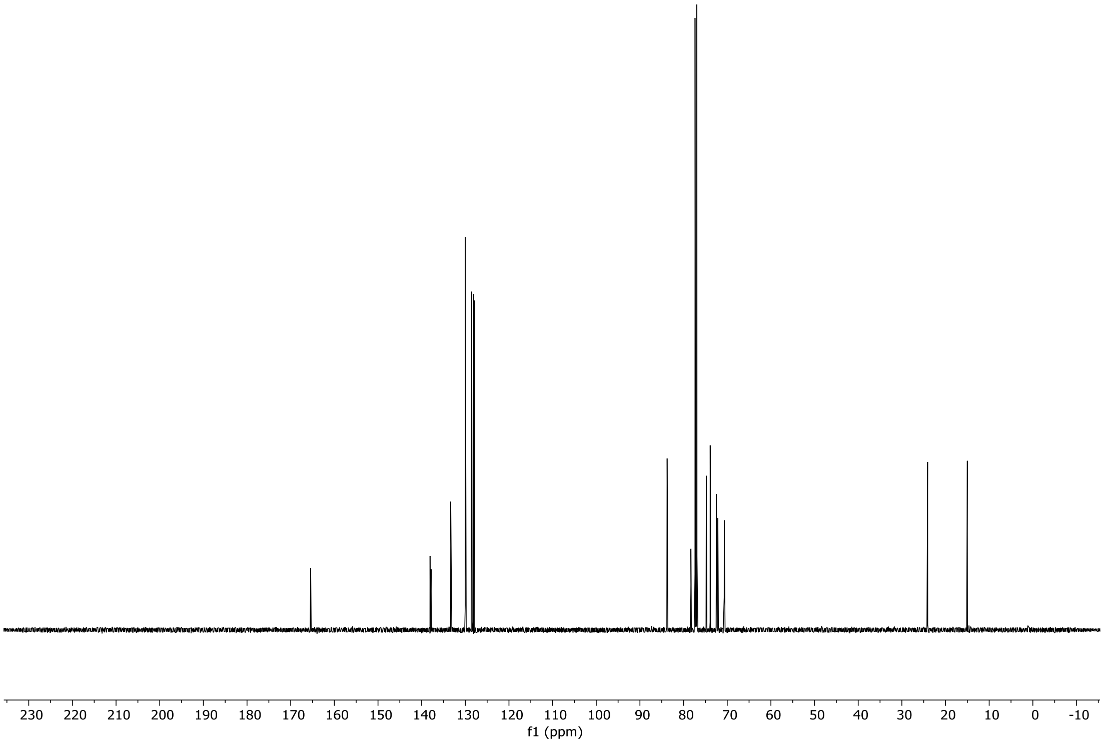

HSQC NMR of S-4 ($CDCl_3$)

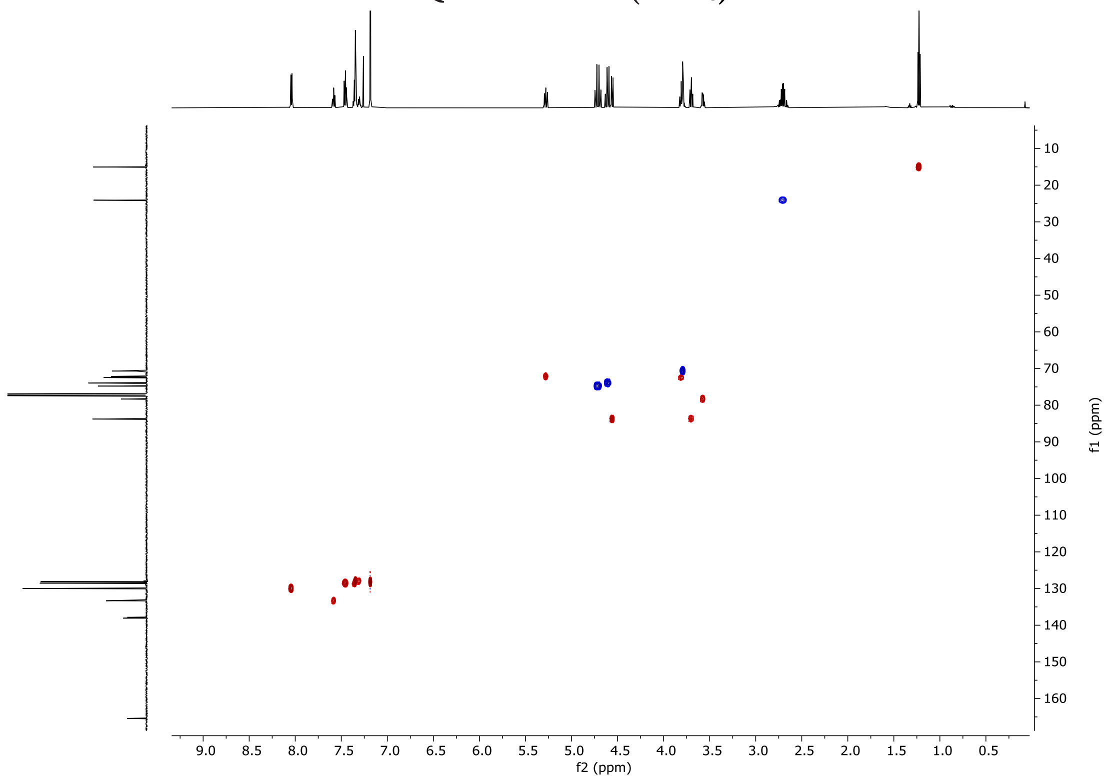

COSY NMR of S-4 ($CDCl_3$)

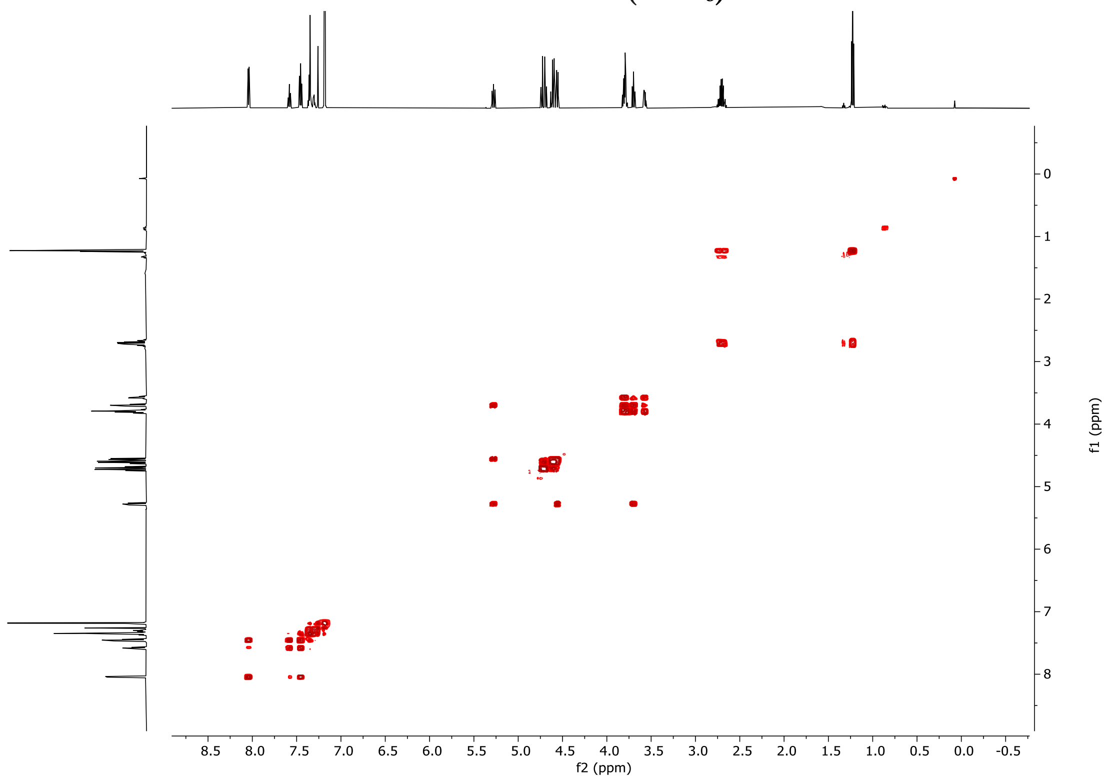

Synthesis of **BB2**

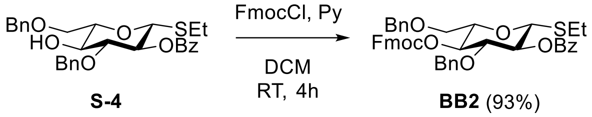

S-4 (769 mg, 1.52 mmol) was dissolved in anhydrous DCM (15 mL) and pyridine (Py) was added (370 μL, 4.6 mmol) to the stirred solution under Ar atmosphere. 9-Fluorenylmethyl chloroformate (FmocCl) (798 mg, 3.1 mmol) was dissolved in anhydrous DCM (5 mL) and added to the reaction mixture. The yellow solution was stirred for 4 h then quenched with an aqueous solution of HCl (1 M). The organic layer was washed three times with an aqueous solution of HCl (1 M), and once with brine. The crude product was purified by silica gel flash column chromatography (Toluene : DCM = 4:1→3:1 then Toluene : EtOAc = 4:1) to give **BB2** as a white solid (1.04 g, 93%).

^{1}H NMR (400 MHz, Chloroform-*d*) δ 8.06 – 7.97 (m, 2H), 7.80 – 7.72 (m, 2H), 7.63 – 7.51 (m, 3H), 7.49 – 7.20 (m, 11H), 7.05 (d, *J* = 3.1 Hz, 5H), 5.34 (t, *J* = 9.6 Hz, 1H), 5.00 (t, *J* = 9.6 Hz, 1H), 4.64 – 4.52 (m, 5H), 4.33 (d, *J* = 7.2 Hz, 2H), 4.13 (t, *J* = 7.1 Hz, 1H), 3.91 (t, *J* = 9.2 Hz, 1H), 3.76 (ddd, *J* = 9.6, 5.0, 3.9 Hz, 1H), 3.67 (d, *J* = 4.2 Hz, 2H), 2.81 – 2.63 (m, 2H), 1.24 (t, *J* = 7.5 Hz, 2H). ^{13}C NMR (101 MHz, Chloroform-*d*) δ 165.17, 154.37, 143.42, 143.24, 141.44, 141.41, 138.00, 137.50, 133.40, 130.02, 129.79, 128.56, 128.47, 128.29, 128.05, 127.97, 127.78, 127.76, 127.31, 125.24, 125.15, 120.22, 83.82, 81.22, 77.53, 75.69, 74.52, 73.73, 72.02, 70.17, 69.83, 46.82, 24.20, 15.02. $[\alpha]_D^{20}$ -26.84 (c 1.01 g/100 mL, $CHCl_3$). IR ν = 1754, 1729, 1248, 1070, 739, 710 cm^{-1}. R_f = 0.53 (Hexane : EtOAc = 3:1). (ESI-HRMS) m/z 753.254 [M+Na]$^+$ ($C_{44}H_{42}O_8SNa$ requires 753.249).

^{1}H NMR of BB2 (400 MHz, $CDCl_3$)

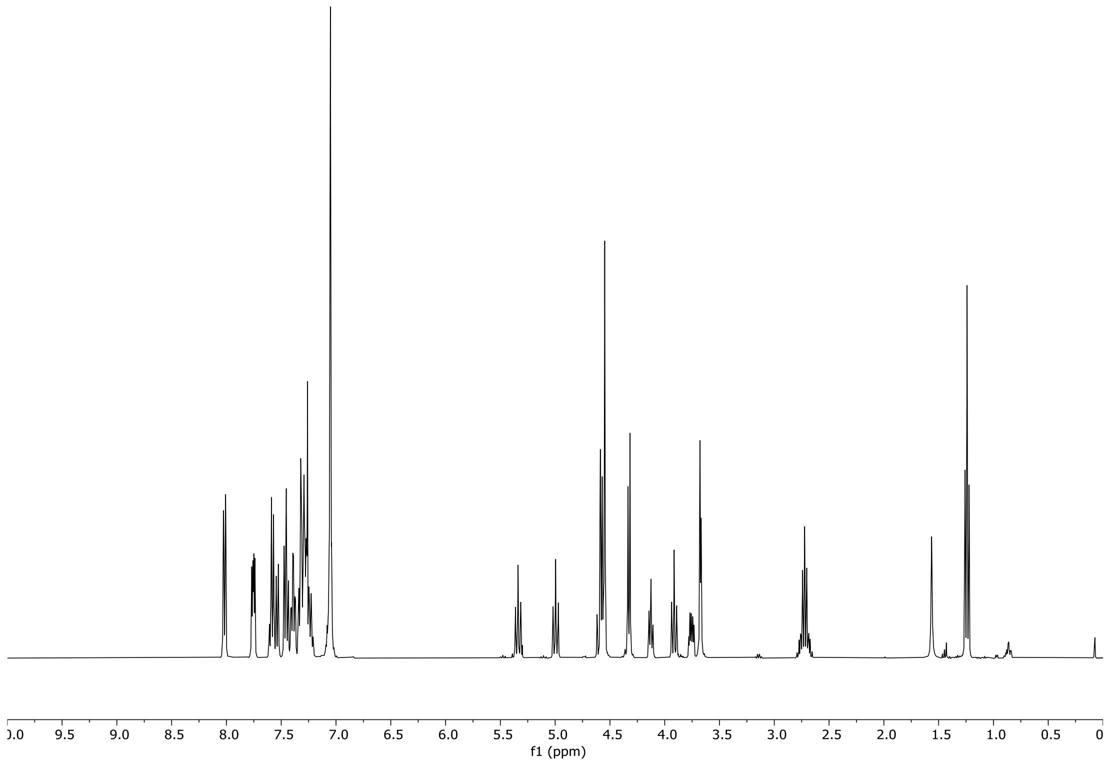

^{13}C NMR of BB2 (101 MHz, $CDCl_3$)

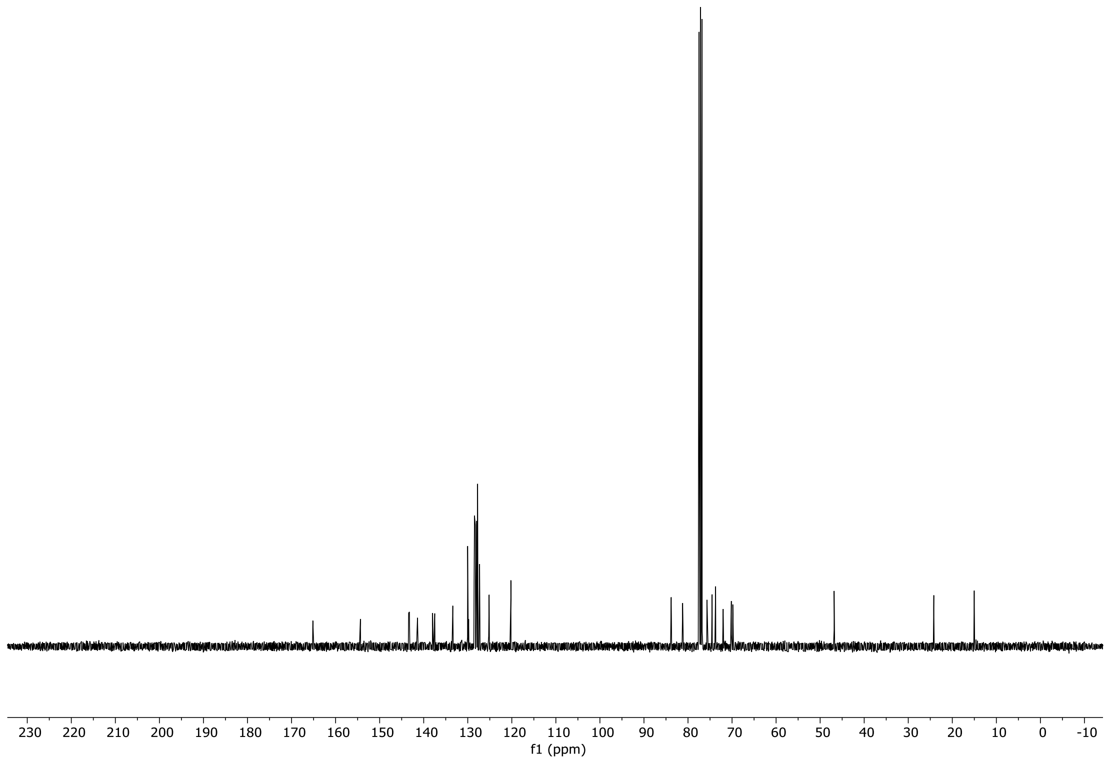

HSQC NMR of BB2 ($CDCl_3$)

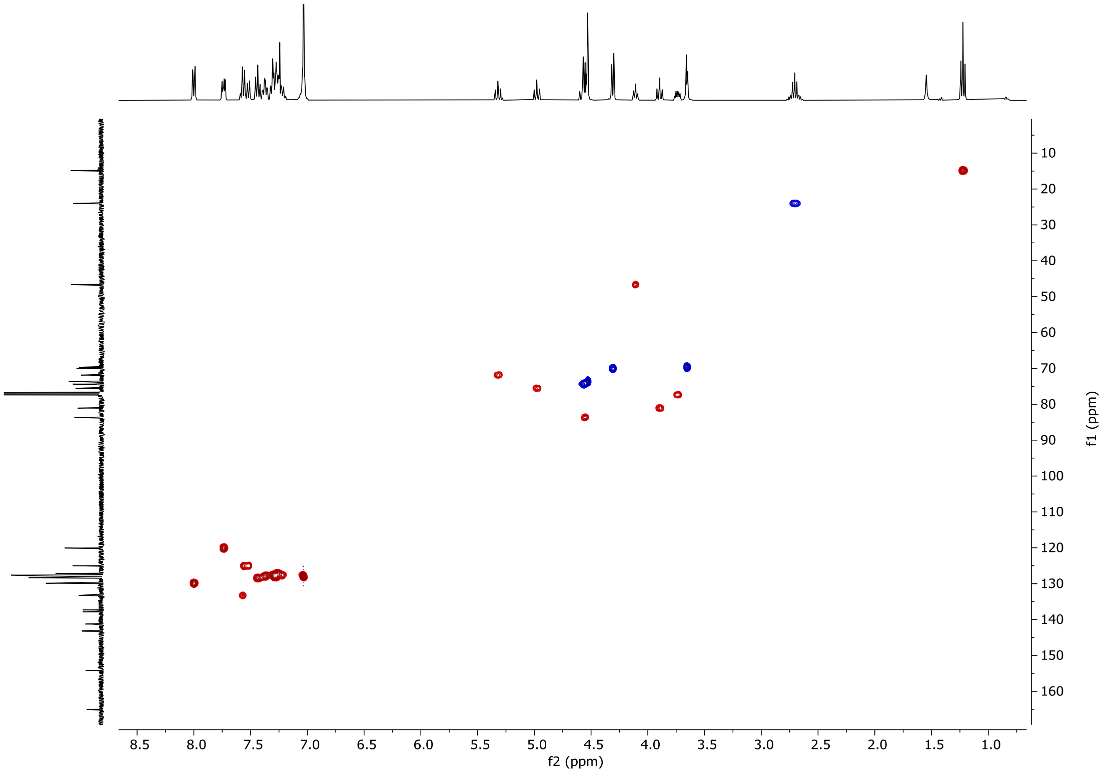

COSY NMR of BB2 ($CDCl_3$)

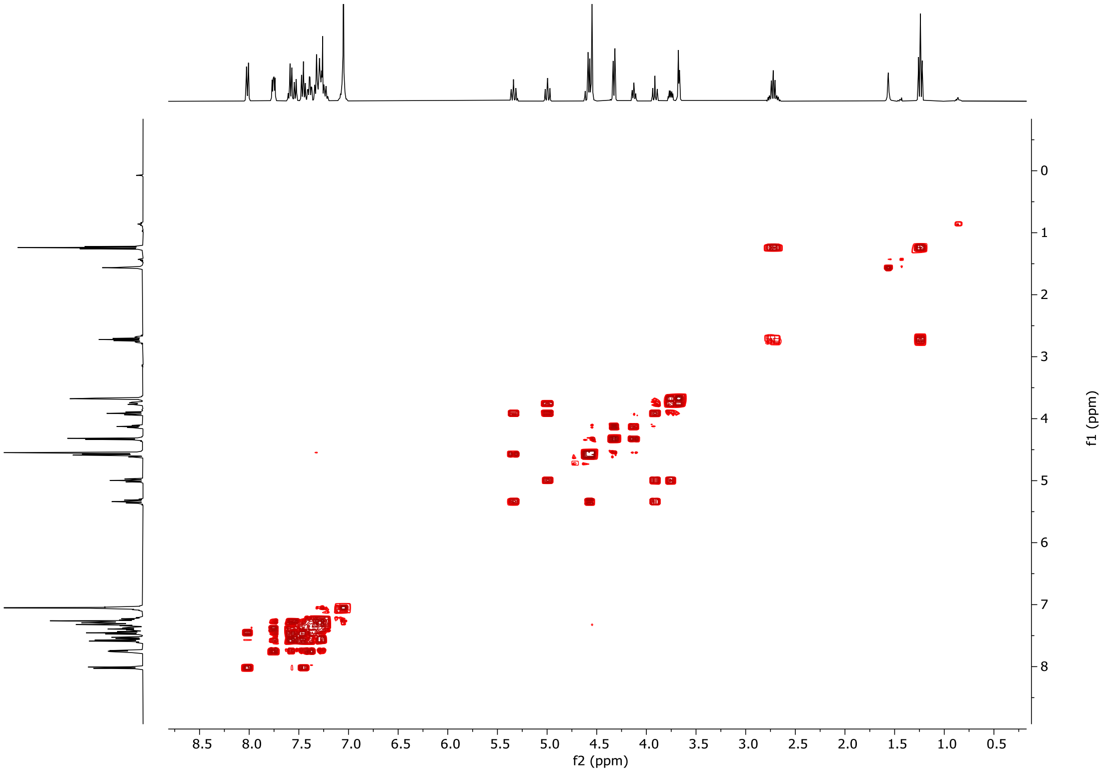

3 Automated Glycan Assembly

3.1 General materials and methods

The automated syntheses were performed on a home-built synthesizer developed at the Max Planck Institute of Colloids and Interfaces.[1] All solvents used were HPLC-grade. The solvents used for the building block, activator, TMSOTf and capping solutions were taken from an anhydrous solvent system (J.C. Meyer) for moisture-sensitive solutions. The building blocks were co-evaporated three times with toluene and dried for 1 h under high vacuum before use. Oven-heated, argon-flushed flasks were used to prepare all moisture-sensitive solutions. Activator, capping, deprotection, acidic wash and building block solutions were freshly prepared and kept under argon during the automation run. All yields of products obtained by AGA were calculated on the basis of resin loading. Resin loading was determined following previously established procedures.[5]

3.2 Preparation of stock solutions

- **Building block solution**: Between 0.06 and 0.10 mmol of building block (depending on the BB, see Module C1 and C2) was dissolved in DCM (1 mL).

- **NIS/TfOH activator solution**: 1.35 g (6.0 mmol) of recrystallized NIS was dissolved in 40 mL of a 2:1 v/v mixture of anhydrous DCM and anhydrous dioxane. Then triflic acid (55 µL, 0.6 mmol) was added. The solution is kept at 0 °C (ice bath) for the duration of the automation run.

- **Fmoc deprotection solution**: A solution of 20% piperidine in DMF (v/v) was prepared.

- **Lev deprotection solution**: Hydrazine acetate (550 mg, 5.97 mmol) was dissolved in pyridine/AcOH/H_2O (40mL, v/v, 32:8:2) and sonicated for 10 min.

- **TMSOTf solution**: TMSOTf (0.45 mL, 2.49 mmol) was added to DCM (40 mL).

- **Capping solution**: A solution of 10% acetic anhydride and 2% methanesulfunic acid in DCM (v/v) was prepared.

3.3 Modules for automated synthesis

Module A: Resin Preparation for Synthesis (20 min)

All automated syntheses were performed on 0.0125 mmol scale. Resin (**L1**, 36 mg or **L2**, 34 mg) was placed in the reaction vessel and swollen in DCM for 20 min at room temperature prior to synthesis. During this time, all reagent lines needed for the synthesis were washed and primed. After the swelling, the resin was washed with DMF, THF, and DCM (three times each with 2 mL for 25 s).

Module B: Acidic Wash with TMSOTf Solution (20 min)

The resin was swollen in 2 mL DCM and the temperature of the reaction vessel was adjusted to -20 °C. Upon reaching the low temperature, TMSOTf solution (1 mL) was added drop wise to the reaction vessel. After bubbling for 3 min, the acidic solution was drained and the resin was washed with 2 mL DCM for 25 s.

Action	Cycles	Solution	Amount	T (°C)	Incubation time
**Cooling**	-	-	-	-20	(15 min)*
**Deliver**	1	DCM	2 mL	-20	-
**Deliver**	1	TMSOTf solution	1 mL	-20	3 min
**Wash**	1	DCM	2 mL	-20	25 sec

*Time required to reach the desired temperature.

Module C1: Thioglycoside Glycosylation (35 min)

The building block solution (0.10 mmol of BB in 1 mL of DCM per glycosylation) was delivered to the reaction vessel. After the set temperature was reached, the reaction was started by drop wise addition of the NIS/TfOH activator solution (1.0 mL, excess). The glycosylation conditions (T_1, T_2, t_1, and t_2) are building block dependent and are reported in a table below. After completion of the reaction, the solution was drained and the resin was washed with DCM, DCM:dioxane (1:2, 3 mL for 20 s) and DCM (two times, each with 2 mL for 25 s). The temperature of the reaction vessel was increased to 25 °C for the next module.

Action	Cycles	Solution	Amount	T (°C)	Incubation time
**Cooling**	-	-	-	T_1	-
**Deliver**	1	BB solution	1 mL	T_1	-
**Deliver**	1	NIS/TfOH activator solution	1 mL	T_1	-
**Reaction time (BB dependent)**	1	-	-	T_1 to T_2	t_1 t_2
**Wash**	1	DCM	2 mL	T_2	5 sec
**Wash**	1	DCM : Dioxane (1:2)	2 mL	T_2	20 sec
**Heating**	-	-	-	25	-
**Wash**	2	DCM	2 mL	> 0	25 sec

The AGA glycosylation conditions employed for thioglycoside BBs were previously reported.[6,7]

BB	Equiv.	t_1 (min)	T_1 (°C)	t_2 (min)	T_2 (°C)
**BB1a**	6.5	5	-20	20	0
**BB2**	6.5	5	-20	20	0

Module C2: Phosphate Glycosylation (45 min)

The building block solution (0.06 mmol of BB in 1 mL of DCM per glycosylation) was delivered to the reaction vessel. After the set temperature was reached, the reaction was started by drop wise addition of the TMSOTf solution (1.0 mL, stoichiometric). After completion of the reaction, the solution was drained and the resin washed with DCM (six times, each with 2 mL for 25 s). The temperature of the reaction vessel was increased to 25 °C for the next module.

Action	Cycles	Solution	Amount	T (°C)	Incubation time
**Cooling**	-	-	-	T_1	-
**Deliver**	1	BB solution	1 mL	T_1	-
**Deliver**	1	TMSOTf solution	1 mL	T_1	-
**Reaction time (BB dependent)**	1			T_1 to T_2	t_1 t_2 min
**Wash**	1	DCM	2 mL	T_2	5 sec
**Heating**	-	-	-	25	-
**Wash**	6	DCM	2 mL	> 0	25 sec

The AGA glycosylation conditions employed for the phosphate BB were previously reported.[7]

BB	Equiv.	t_1 (min)	T_1 (°C)	t_2 (min)	T_2 (°C)
**BB1b**	5	5	-35	40	-15

Module D: Capping (30 min)

The resin was washed with DMF (two times with 2 mL for 25 s) and the temperature of the reaction vessel was adjusted to 25 °C. 2 mL of Pyridine solution (10% in DMF) was delivered into the reaction vessel. After 1 min, the reaction solution was drained and the resin washed with DCM (three times with 3 mL for 25 s). 4 mL of capping solution was delivered into the reaction vessel. After 20 min, the reaction solution was drained and the resin washed with DCM (three times with 3 mL for 25 s).

Action	Cycles	Solution	Amount	T (°C)	Incubation time
**Heating**	-	-	-	25	(5 min)*
**Wash**	2	DMF	2 mL	25	25 sec
**Deliver**	1	10% Pyridine in DMF	2 mL	25	1 min
**Wash**	3	DCM	2 mL	25	25 sec
**Deliver**	1	Capping Solution	4 mL	25	20 min
**Wash**	3	DCM	2 mL	25	25 sec

*Time required to reach the desired temperature.

Module E: Fmoc Deprotection (9 min)

The resin was washed with DMF (three times with 2 mL for 25 s) and the temperature of the reaction vessel was adjusted to 25 °C. 2 mL of Fmoc deprotection solution was delivered to the reaction vessel and kept under Ar bubbling. After 5 min, the reaction solution was drained and the resin washed with DMF (three times with 3 mL for 25 s) and DCM (five times each with 2 mL for 25 s). The temperature of the reaction vessel was decreased to -20 °C for the next module.

Action	Cycles	Solution	Amount	T (°C)	Incubation time
**Wash**	3	DMF	2 mL	25	25 sec
**Deliver**	1	Fmoc depr. Solution 1	2 mL	25	5 min
**Wash**	1	DMF	2 mL		
**Cooling**	-	-	-	-20	-
**Wash**	3	DMF	2 mL	< 25	25 sec
**Wash**	5	DCM	2 mL	< 25	25 sec

3.4 Post-AGA manipulations

Module F: On-resin Methanolysis

The resin was suspended THF (4 mL). MeONa in MeOH (0.5 M, 0.4 mL) was added and the suspension was gently shaken at room temperature. After micro-cleavage (see Module G1) indicated the complete removal of benzoyl groups, the resin was repeatedly washed with MeOH (2mL x 3) and DCM (2mL x 3). For $\mathbf{D_7}$, $\mathbf{D_8}$ and $\mathbf{D_9}$ a higher amount of MeONa in MeOH (0.5 M, 0.8 mL) was used.

Module G: Cleavage from Solid Support

The oligosaccharides were cleaved from the solid support using a continuous-flow photoreactor as described previously.[8]

Module G1: Micro-cleavage from Solid Support

Trace amount of resin (around 20 beads) was dispersed in DCM (0.1 mL) and irradiated with a UV lamp (6 W, 356 nm) for 10 min. ACN (10 µL) was then added to the resin and the resulting solution analyzed by MALDI.

Module H1: Hydrogenolysis

The crude compound obtained from *Module G* was dissolved in 2 mL of EtOAc:*t*BuOH:H_2O (2:1:1). 100% by weight Pd/C (10%) was added and the reaction was stirred in a pressurized reactor under 4 bar pressure of H_2. The reaction progress was monitored to avoid undesired side products formation (*i.e.* degradation of reducing end).[9] Upon completion, the reaction was filtered and washed with EtOAc, *t*BuOH and H_2O (4 mL each). The filtrates were concentrated *in vacuo*. For $\mathbf{D_8}$ and $\mathbf{D_9}$, the products were kept in solution to prevent aggregation. The organic phase was removed from the reaction mixture and the aqueous phase was washed with EtOAc

two times. The remaining aqueous solution was concentrated to about half the volume to remove traces of EtOAc and *t*BuOH.

Module H2: Hydrogenolysis at ambient pressure

The crude compound obtained from *Module G* was dissolved in 2 mL of EtOAc:*t*BuOH:H_2O (2:1:1). 100% by weight Pd/C (10%) was added and the reaction was stirred in a flask equipped with a H_2 balloon. The reaction progress was monitored to avoid undesired side products formation. Upon completion, the reaction was filtered and washed with EtOAc, *t*BuOH, ACN and H_2O (4 mL each). The filtrates were concentrated *in vacuo*.

Module I: Purification

The purification of the crudes was conducted using a C_{18} silica column or reverse phase HPLC (Agilent 1200 Series, **Method B** and **Method C**). The pure compound was analyzed using analytical HPLC (Agilent 1200 Series, **Method A**).

- **Method A1:** (Synergi Hydro RP18 column, Phenomenex, 250 x 4.6 mm), flow rate of 1.0 mL/min with H_2O (0.1% formic acid) and ACN as eluents [isocratic (5 min), linear gradient to 30% ACN (30 min), linear gradient to 100% ACN (5 min), isocratic 100% ACN (5 min)].
- **Method A2 (Prep):** (Synergi Hydro RP18 column, Phenomenex, 250 x 10 mm) flow rate of 4.0 mL/min with H_2O (0.1% formic acid) and ACN as eluents [isocratic (5 min), linear gradient to 30% ACN (30 min), linear gradient to 100% ACN (5 min), isocratic 100% ACN (5 min)].
- **Method C:** (Manual reverse phase C_{18} silica gel column chromatography): H_2O (0.1% formic acid, 10 mL), 3% MeOH (10 mL), 6% MeOH (10 mL), 9% MeOH (10 mL), 15% MeOH (10 mL).
- **Method D1:** (YMC-Diol-300 column, 150 x 4.6 mm) flow rate of 1.0 mL/min with EtOAc and Hexane as eluents [isocratic 20% EtOAc (5 min), linear gradient to 50% EtOAc (10 min), linear gradient to 80% EtOAc (20 min), linear gradient to 100% EtOAc (10 min)].
- **Method D2 (Prep):** (YMC-Diol-300 column, 150 x 20 mm) flow rate of 15.0 mL/min with EtOAc and Hexane as eluents [isocratic 20% EtOAc (5 min), linear gradient to 50% EtOAc (10 min), linear gradient to 80% EtOAc (20 min), linear gradient to 100% EtOAc (10 min)].

Following final purification, all deprotected products were lyophilized on a Christ Alpha 2-4 LD plus freeze dryer prior to characterization.

3.5 Oligosaccharides synthesis

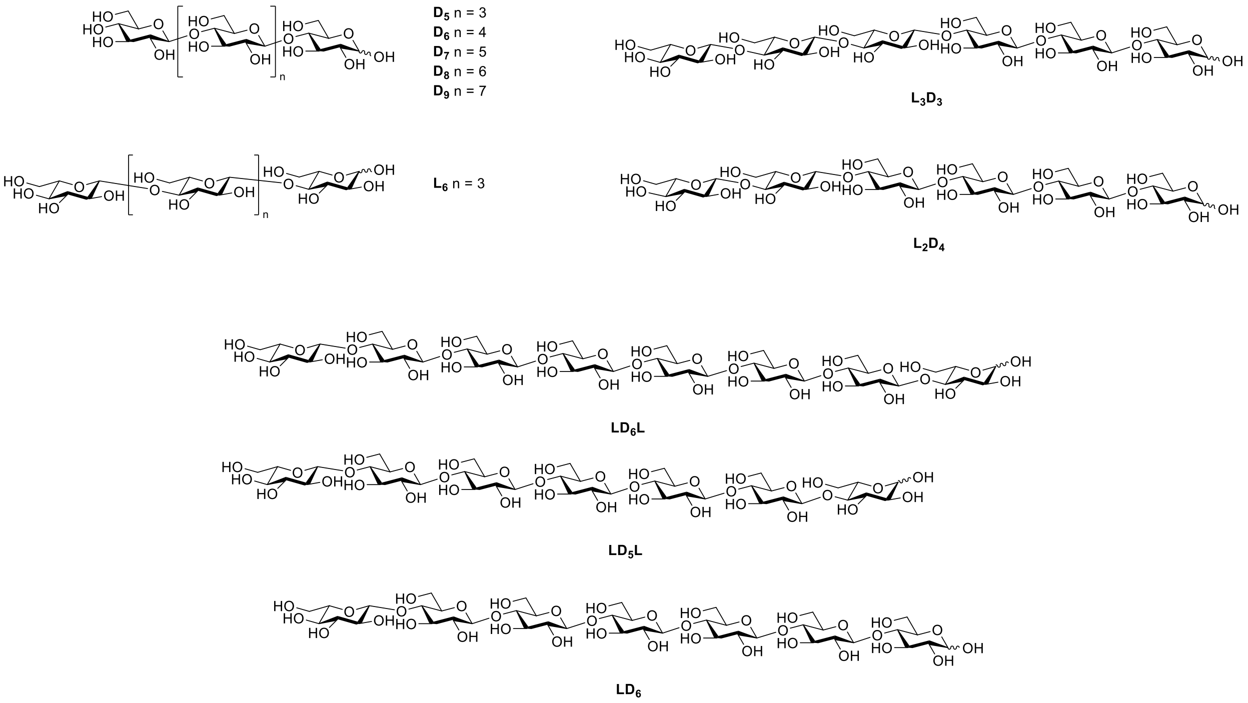

Figure S2 Collection of cellulose analogues synthesized by AGA.

3.5.1 D_5

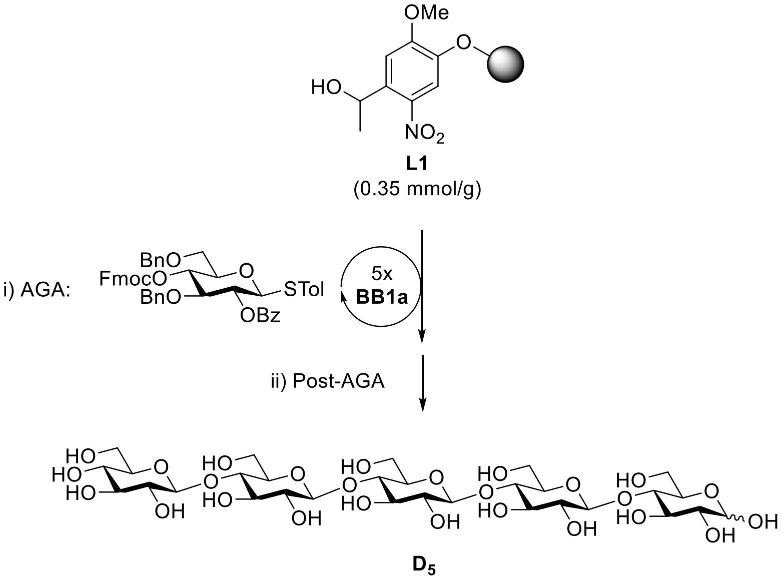

Step	Modules		Notes
AGA	**L1**	**A**	**L1** swelling
	(**BB1a**)x5	(**B**, **C1**, **D**, **E**)x5	**C1**: (**BB1a**, -20 °C for 5 min, 0 °C for 20 min)
Post-AGA			**F**: (16 h)
		**F**, **G**, **H2**, **I**	**H2**: (4 h)
			**I**: (Method C)

Automated synthesis, global deprotection, and purification afforded compound $\mathbf{D_5}$ as a white solid (4.0 mg, 40% overall yield).

Analytical data for $\mathbf{D_5}$:
^{1}H NMR (400 MHz, D_2O) δ 5.09 (d, J = 3.8 Hz, 0.4 H, H1^{I}-α), 4.53 (d, J = 7.9 Hz, 0.6 H, H1^{I}-β), 4.44-4.33 (m, 4H, H1$^{II, III, IV, V}$), 3.89 – 3.77 (m, 4H), 3.78 – 3.65 (m, 5H), 3.64 – 3.56 (m, 2H), 3.56 – 3.41 (m, 11H), 3.41 – 3.32 (m, 2H), 3.29 (d, J = 9.1 Hz, 1H), 3.26 – 3.11 (m, 5H). ^{13}C NMR (176 MHz, D_2O) δ 102.56, 102.35, 95.76, 91.83, 78.65, 78.50, 78.39, 78.30, 78.27, 75.99, 75.49, 74.82, 74.80, 74.26, 74.04, 74.02, 74.00, 73.90, 73.15, 72.97, 72.96, 72.93, 71.31, 71.23, 70.13, 69.46, 60.58, 60.02, 59.90, 59.86. (ESI-HRMS) m/z 851.266 [M+Na]$^+$ ($C_{30}H_{52}O_{26}Na$ requires 851.264).

RP-HPLC of D_5 (ELSD trace, Method A1, t_R = 13.4 min)

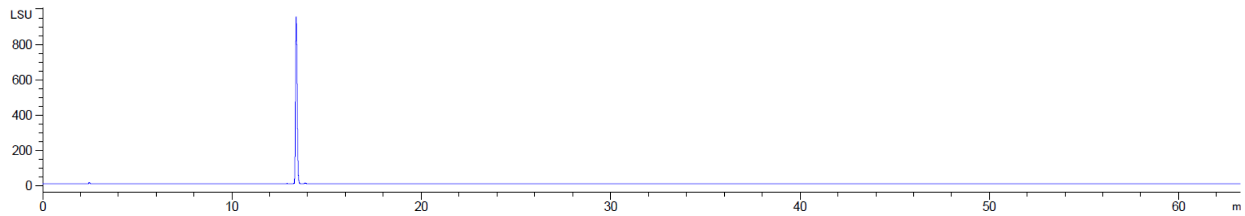

^{1}H NMR of D_5 (400 MHz, D_2O)

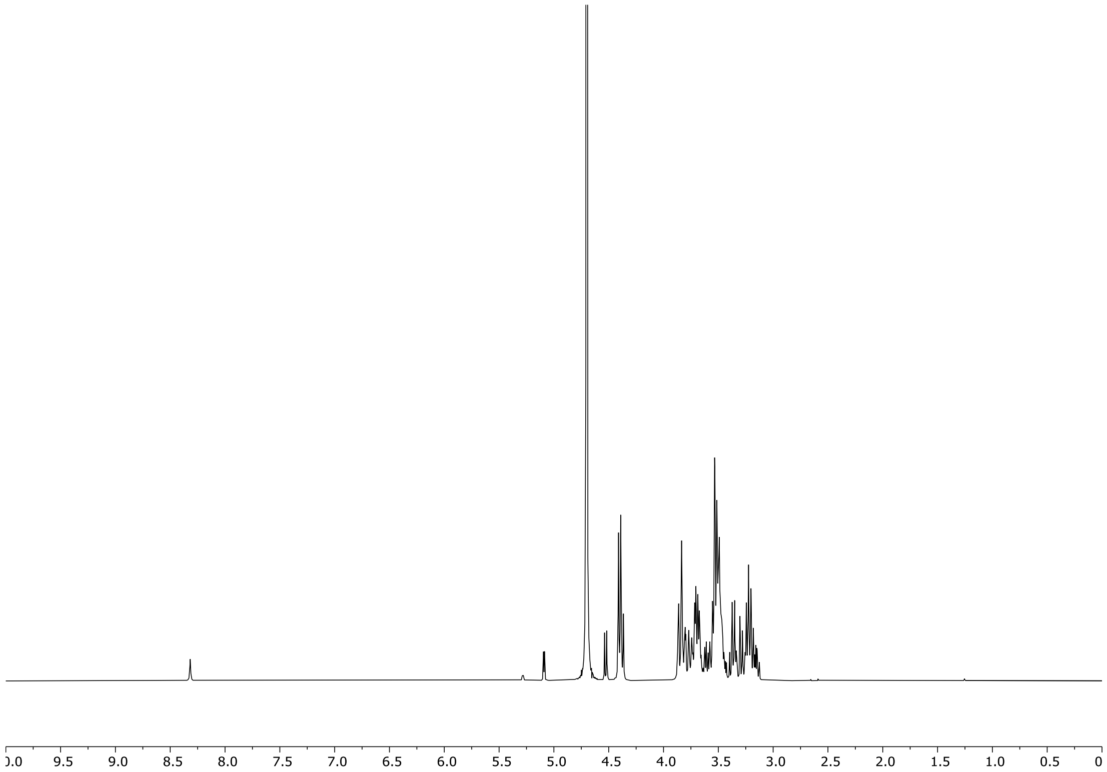

^{13}C NMR of D_5 (176 MHz, D_2O)

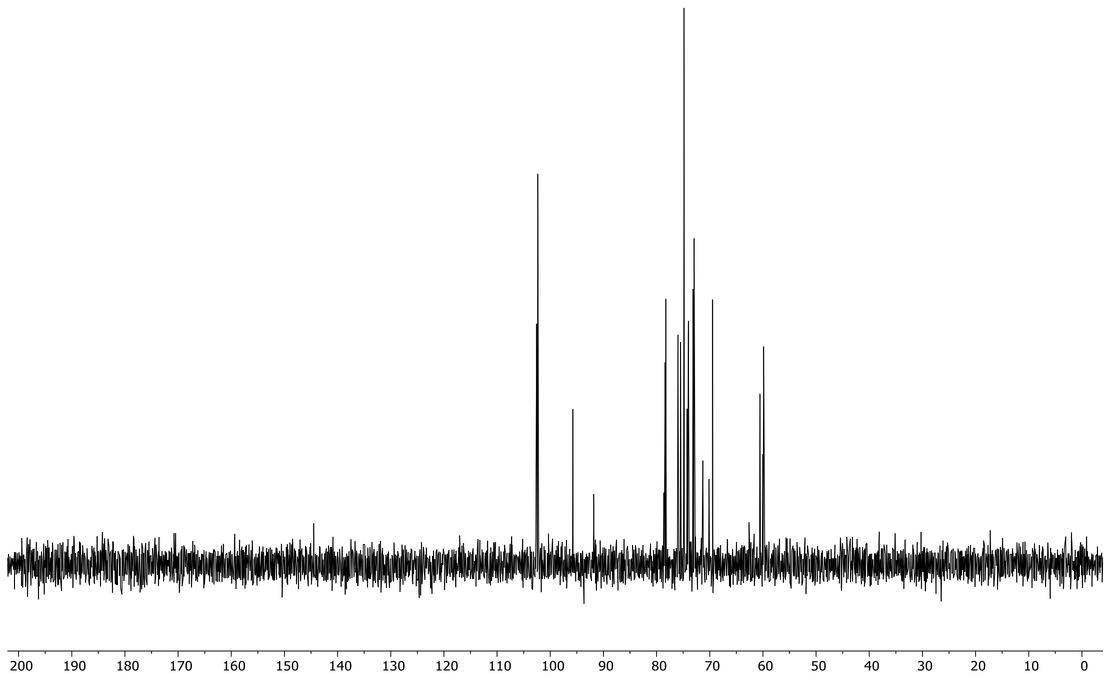

HSQC NMR of D_5 (D_2O)

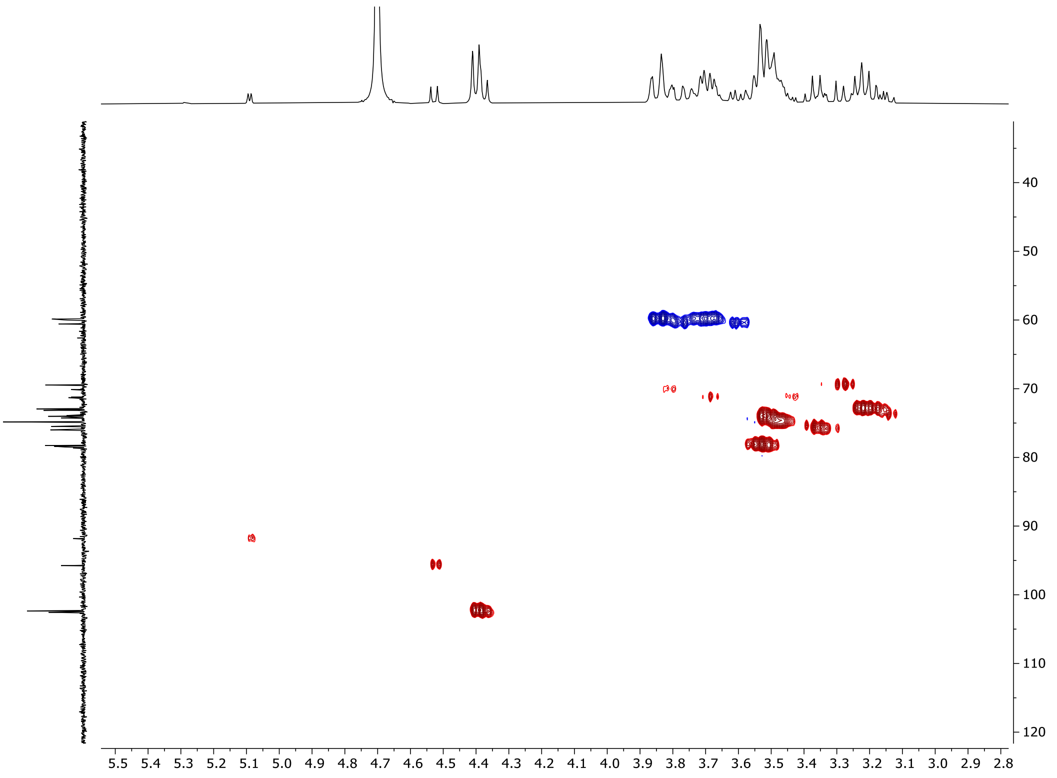

3.5.2 D_6

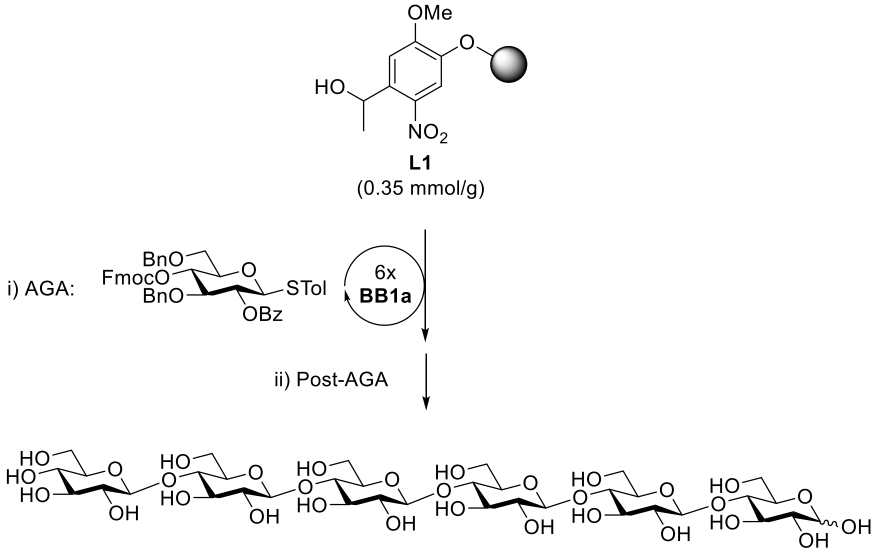

D_6

Step	Modules		Notes
AGA	**L1**	**A**	**L1** swelling
	(**BB1a**)x6	(**B**, **C1**, **D**, **E**)x6	**C1**: (**BB1a**, -20 °C for 5 min, 0 °C for 20 min)
Post-AGA		**F**, **G**, **H2**, **I**	**F**: (3 d)<br>**H2**: (4 h)<br>**I**: (Method C)

Automated synthesis, global deprotection, and purification afforded compound **D_6** as a white solid (8.0 mg, 57% overall yield).

Analytical data for **D_6** were in good agreement with previously reported data.[6]
^{1}H NMR (600 MHz, D_2O) δ 5.24 (d, J = 3.7 Hz, 0.4H, H1^{I}-α), 4.68 (d, J = 7.9 Hz, 0.6H, H1^{I}-β), 4.58 – 4.50 (m, 5H, H1$^{II, III, IV, V, VI}$), 4.05 – 3.90 (m, 6H), 3.90 – 3.80 (m, 6H), 3.79 – 3.57 (m, 16H), 3.55 – 3.48 (m, 2H), 3.46 – 3.41 (m, 1H), 3.41 – 3.27 (m, 5H).

^{1}H NMR of D_6 (400 MHz, D_2O)

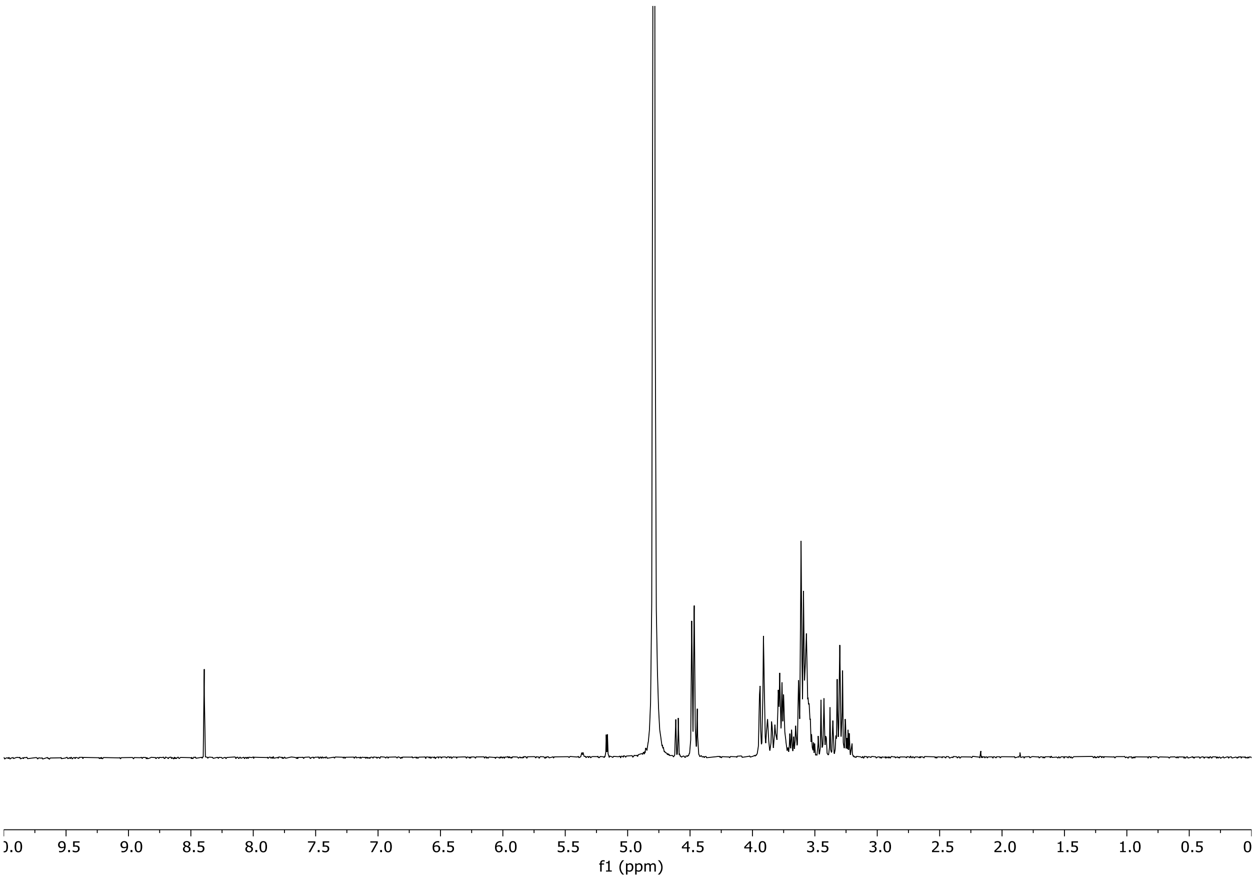

3.5.3 D_7

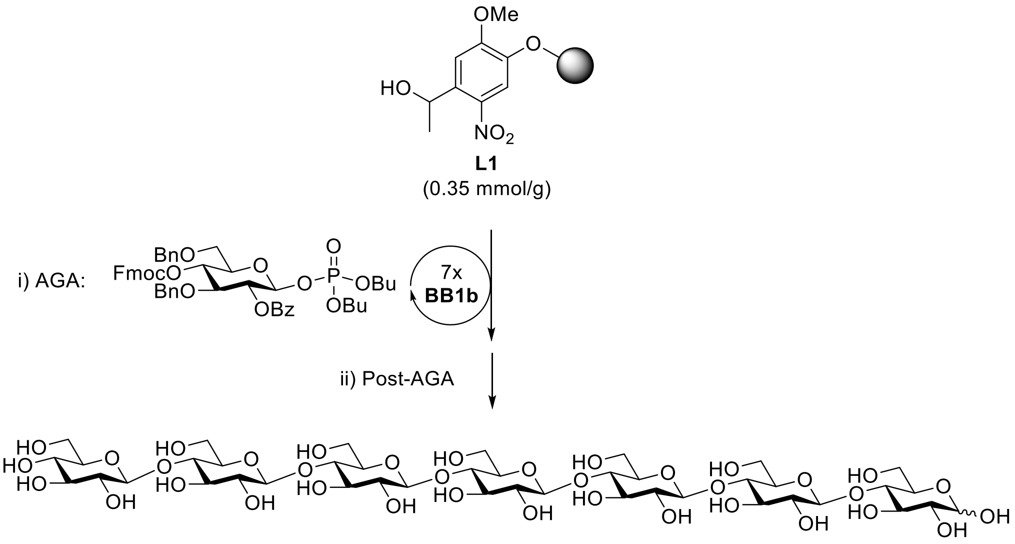

D_7

Step	Modules		Notes
AGA	**L1**<br>(**BB1b**)x7	**A**<br>(**B**, **C2**, **D**, **E**)x7	**L1** swelling<br>**C1**: (**BB1b**, -35 °C for 5 min, -15 °C for 40 min)
Post-AGA		**F**, **G**, **H2**, **I**	**F**: (24 h)<br>**H2**: (4 h)<br>**I**: (Method C)

Automated synthesis, global deprotection, and purification afforded compound **D_7** as a white solid (0.9 mg, 6% overall yield).

Analytical data for **D_7**:
^{1}H NMR (400 MHz, D_2O) δ 5.07 (d, *J* = 3.8 Hz, 0.4H, H1^{I}-α), 4.51 (d, *J* = 8.0 Hz, 0.6H, H1^{I}-β), 4.42 – 4.31 (m, 6H, H1$^{II, III, IV, V, VI, VII}$), 3.87-3.76 (m, 6H), 3.75 – 3.62 (m, 7H), 3.61 – 3.39 (m, 19H), 3.38 – 3.09 (m, 10H). ^{13}C NMR (176 MHz, D_2O) δ 102.53, 102.32, 78.34, 78.22, 75.95, 74.79, 73.96, 72.90, 69.42, 60.53, 59.82. (ESI-HRMS) m/z 1175.370 [M+Na]$^+$ ($C_{42}H_{72}O_{36}Na$ requires 1175.370).

RP-HPLC of D_7 (ELSD trace, Method A1, t_R = 14.2 min)

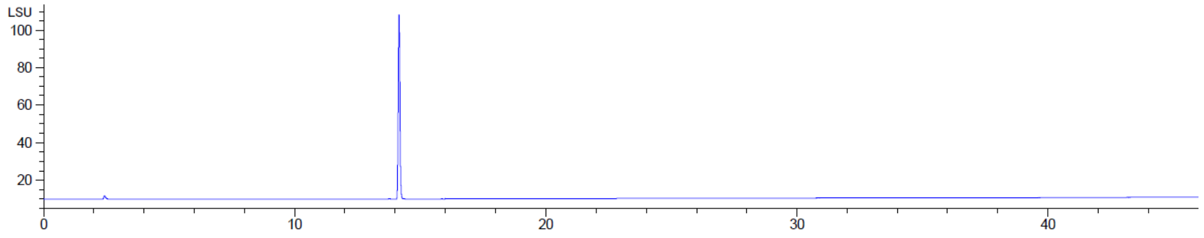

^{1}H NMR of D_7 (400 MHz, D_2O)

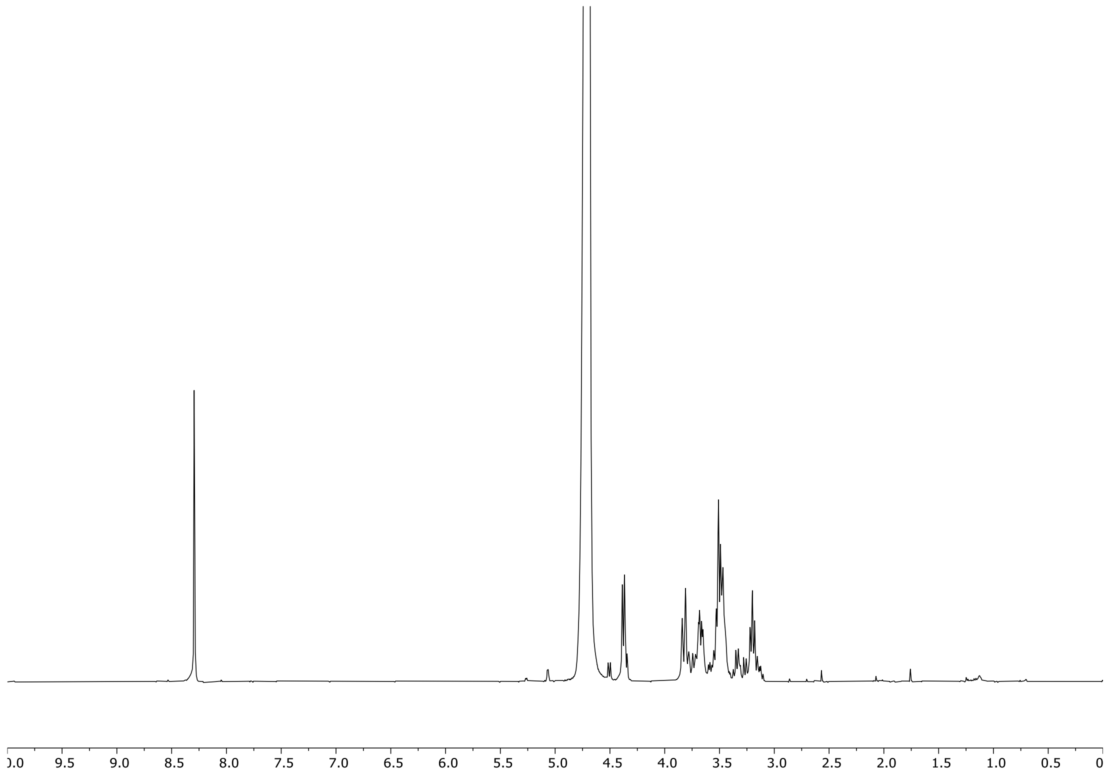

^{13}C NMR of D_7 (176 MHz, D_2O)

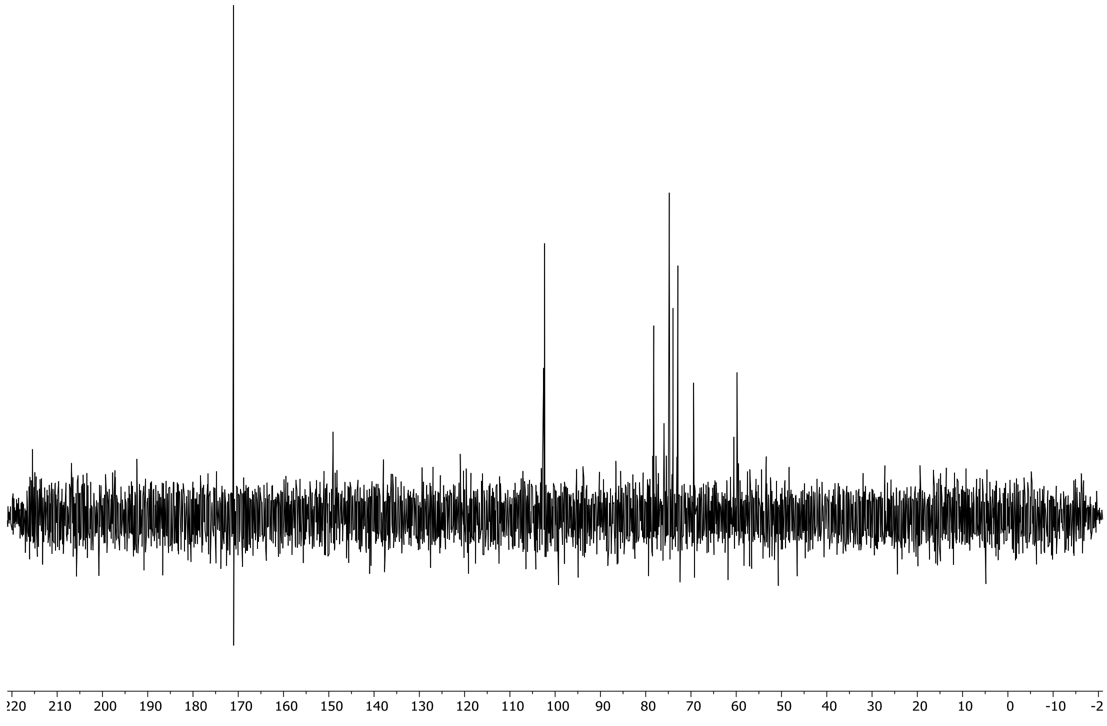

HSQC NMR of D_7 (D_2O)

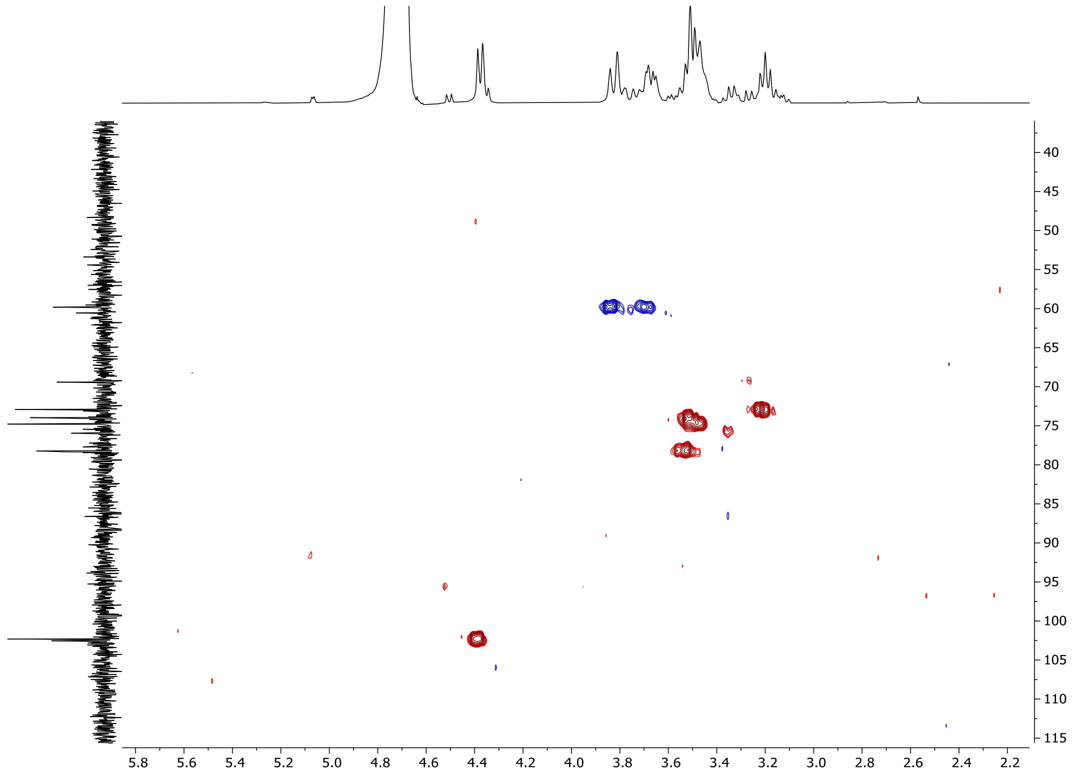

3.5.4 D_8

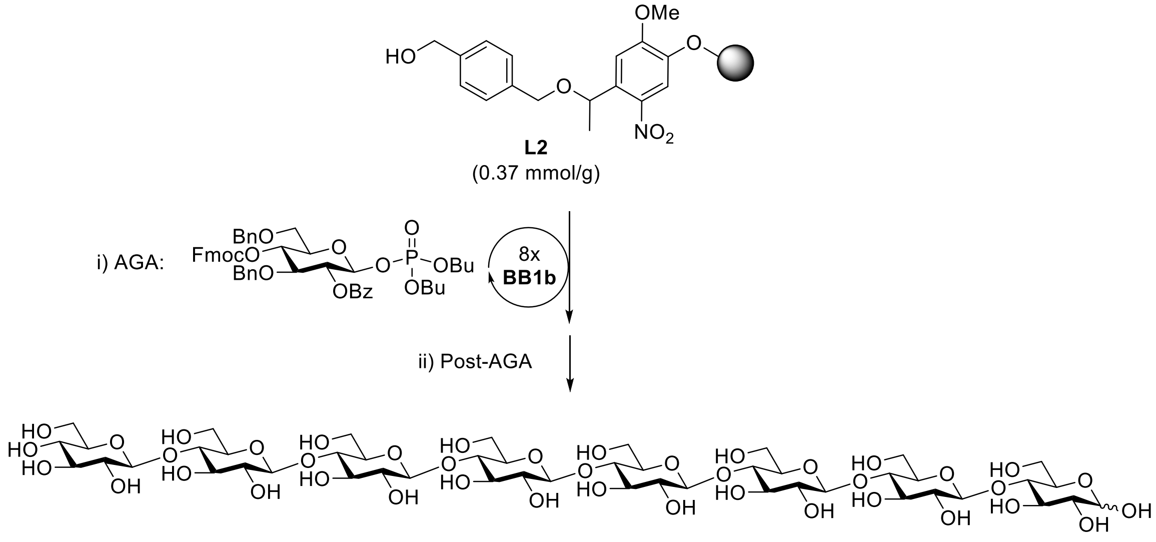

D_8

Step	Modules		Notes
AGA	**L1**	**A**	**L1** swelling
	(**BB1b**)x8	(**B**, **C2**, **D**, **E**)x8	**C1**: (**BB1b**, -35 °C for 5 min, -15 °C for 40 min)
Post-AGA		**F**, **G**, **I**[1], **H2**, **I**[2]	**F**: (24 h)
			**H2**: (18 h)
			**I**[1]: (Method D2, t_R = 32.5 min)
			**I**[2]: (Method C)

Automated synthesis, solid phase methanolysis, photocleavage and purification afforded the semiprotected compound **P-D_8** as a white solid (10 mg, 29% yield). Due to severe insolubility, **D_8** was isolated in traces amount after hydrogenolysis and purification.

Analytical data for **P-D$_8$**:

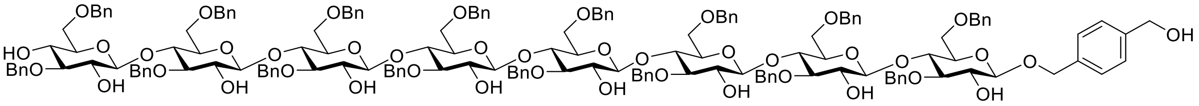

P-D$_8$

^{1}H NMR (600 MHz, $CDCl_3$) δ 7.41 – 7.22 (m, 84H), 4.96 – 4.79 (m, 15H), 4.76 (d, *J* = 11.5 Hz, 1H), 4.71 (s, 2H), 4.69 (d, *J* = 12.1 Hz, 1H), 4.64 (d, *J* = 11.8 Hz, 1H), 4.61 – 4.56 (m, 4H), 4.55 – 4.45 (m, 9H), 4.42 (s, 2H), 4.40 – 4.34 (m, 6H), 4.02 (dd, *J* = 9.7, 8.5 Hz, 1H), 3.98 – 3.85 (m, 7H), 3.81 (dd, *J* = 11.5, 2.3 Hz, 2H), 3.72 – 3.54 (m, 10H), 3.51 – 3.31 (m, 24H), 3.25 (t, *J* = 9.0 Hz, 1H), 3.21 (dt, *J* = 9.7, 2.9 Hz, 1H), 3.19 – 3.09 (m, 6H). ^{13}C NMR (151 MHz, $CDCl_3$) δ 140.69, 139.24, 139.21, 139.16, 138.99, 138.80, 137.78, 137.57, 137.35, 137.31, 137.29, 136.54, 129.50, 128.47, 128.43, 128.37, 128.35, 128.30, 128.27, 128.26, 128.19, 128.13, 128.05, 128.03, 128.01, 127.90, 127.85, 127.73, 127.69, 127.39, 127.31, 127.23, 127.19, 127.14, 126.94, 126.80, 126.70, 126.66, 126.64, 103.70, 103.66, 103.56, 103.43, 103.30, 101.75, 83.53, 83.49, 83.47, 83.37, 76.67, 75.71, 75.66, 75.58, 75.40, 75.13, 74.62, 74.56, 74.47, 74.45, 74.38, 74.28, 74.23, 74.16, 73.71, 73.65, 73.61, 73.57, 73.52, 71.70, 70.77, 70.48, 68.65, 67.79, 65.09. (MALDI-TOF) m/z 2898.5 [M+Na]$^+$ ($C_{168}H_{186}O_{42}Na$ requires 2898.2).

^{1}H NMR of P-D$_8$ (600 MHz, $CDCl_3$)

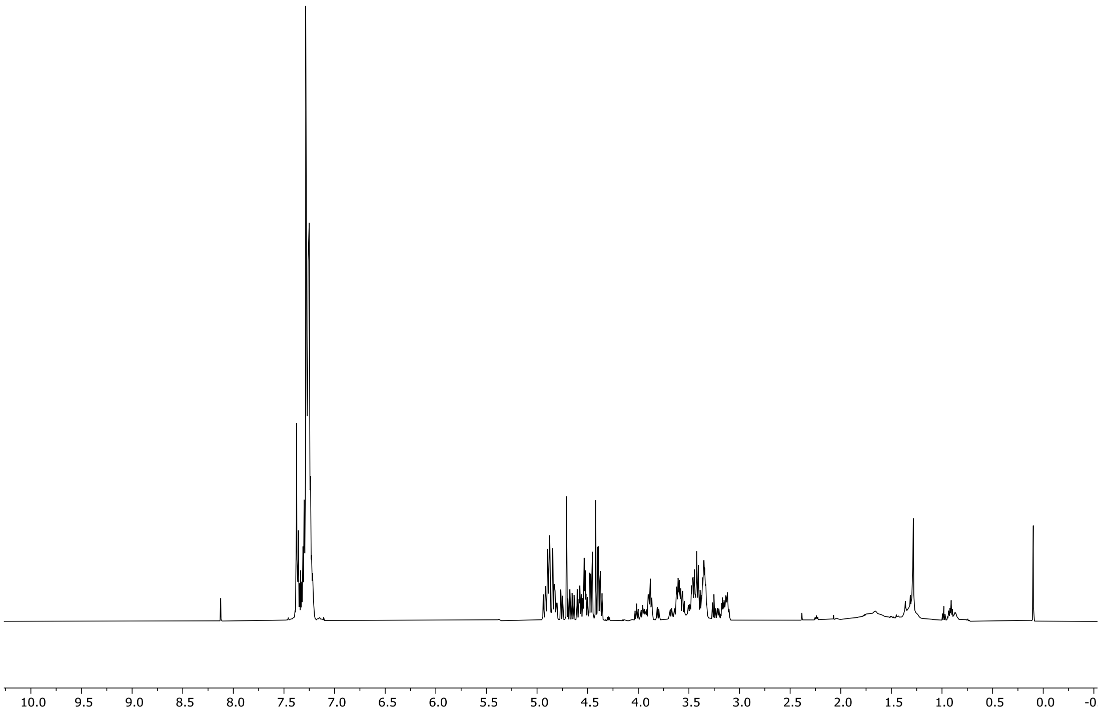

^{13}C NMR of P-D_8 (151 MHz, $CDCl_3$)

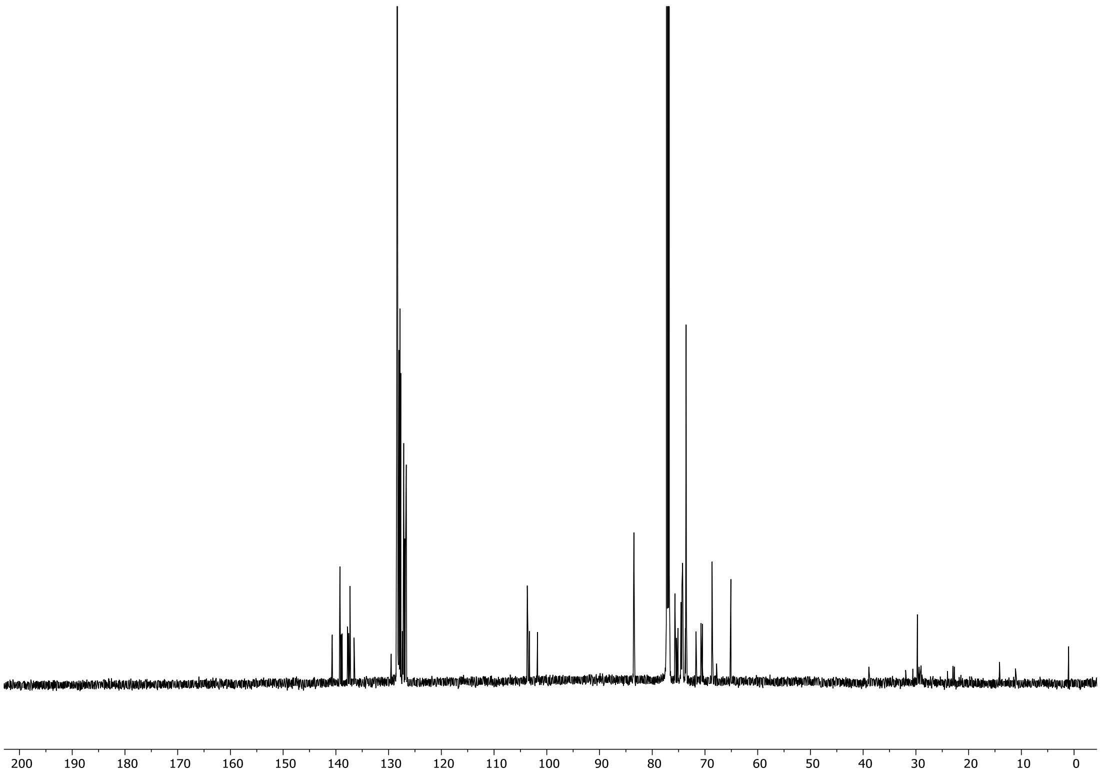

HSQC NMR of P-D_8 ($CDCl_3$)

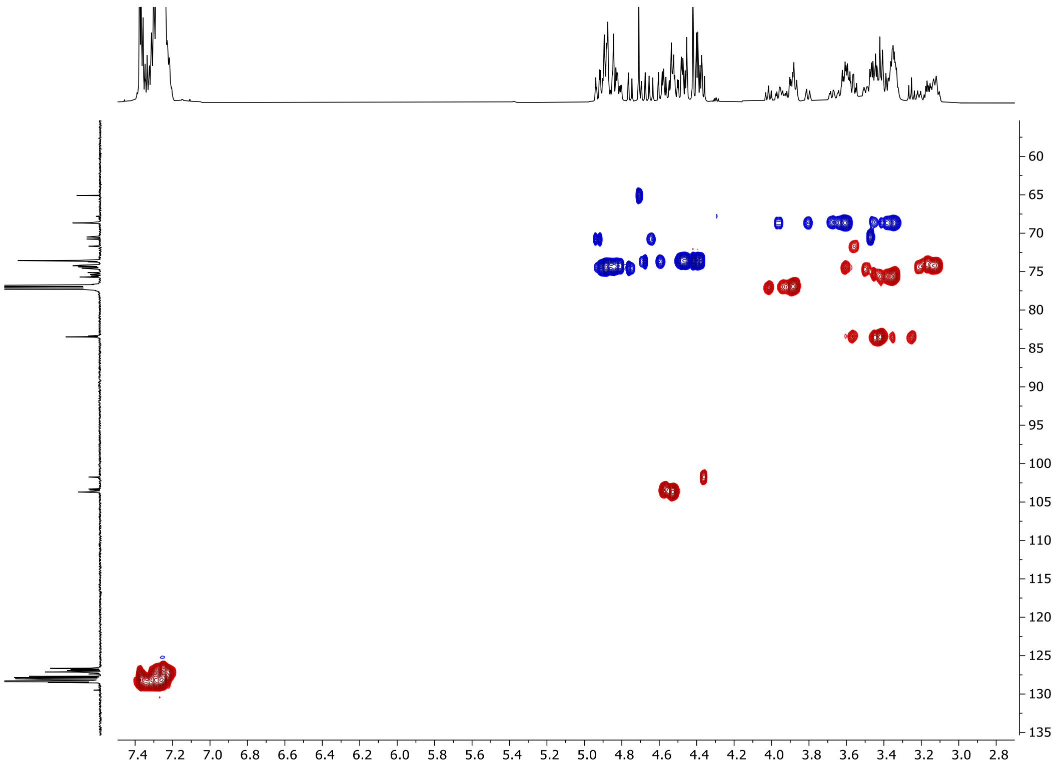

Analytical data for **D_8**:
^{1}H NMR (600 MHz, D_2O) δ 5.11 (d, J = 3.7 Hz, 0.4H), 4.55 (d, J = 7.1 Hz, 0.6H), 4.42-4.37 (m, 7H), 3.91 – 3.78 (m, 10H), 3.77 – 3.67 (m, 9H), 3.62 (dd, J = 12.4, 5.8 Hz, 1H), 3.59 – 3.45 (m, 18H), 3.42 – 3.35 (m, 2H), 3.30 (t, J = 9.4 Hz, 1H), 3.27 – 3.14 (m, 7H). (ESI-HRMS) m/z 1337.429 $[M+Na]^+$ ($C_{48}H_{82}O_{41}Na$ requires 1337.422).

RP-HPLC of D_8 (ELSD trace, Method A1, t_R = 14.4 min)

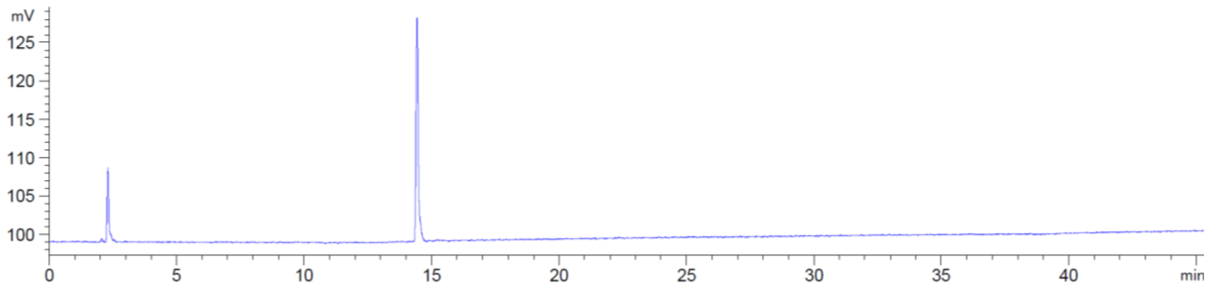

^{1}H NMR of D_8 (600 MHz, D_2O)

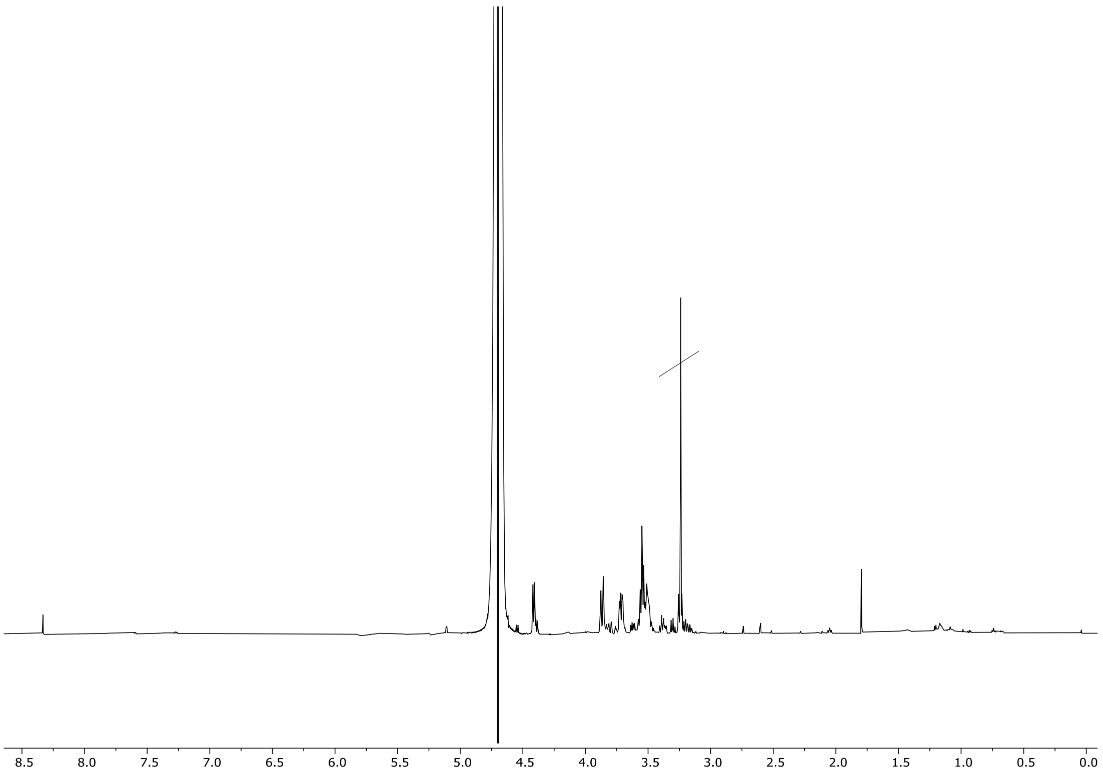

3.5.5 D_9

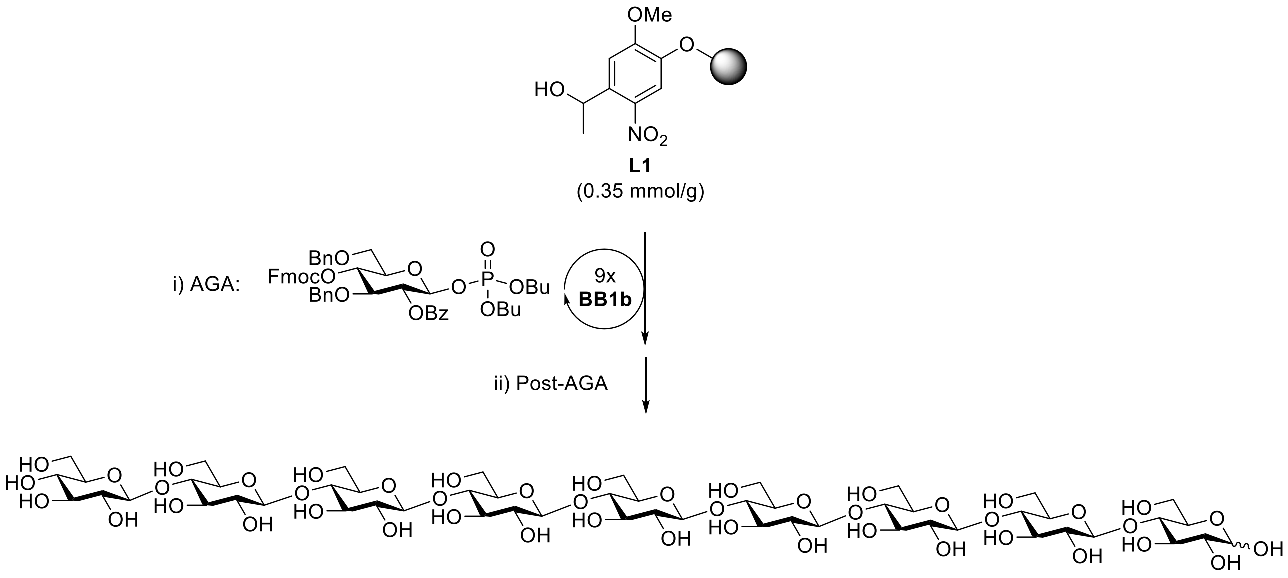

D_9

Step	Modules		Notes
AGA	**L1**<br>(**BB1b**)x9	**A**<br>(**B**, **C2**, **D**, **E**)x9	**L1** swelling<br>**C1**: (**BB1b**, -35 °C for 5 min, -15 °C for 40 min)
Post-AGA		**F**, **G**, **I**[1], **H2**, **I**[2]	**F**: (24 h)<br>**H2**: (18 h)<br>**I**[1]: (Method D2, t_R = 35.0, 36.2 min)<br>**I**[2]: (Method C)

Automated synthesis, solid phase methanolysis, photocleavage and purification afforded the semiprotected compound **P-D₉** as a white solid (15 mg, 39% yield). Due to severe insolubility, **D_9** was isolated in traces amount after hydrogenolysis and purification.

Analytical data for **P-D$_9$**:

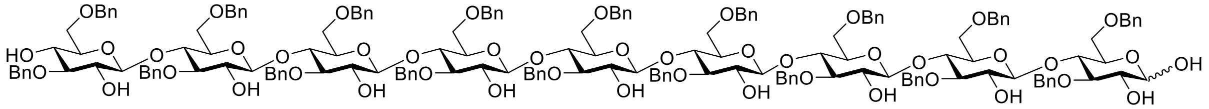

P-D$_9$

^{1}H NMR (600 MHz, $CDCl_3$) δ 7.40 – 7.18 (m, 90H), 5.28 (d, *J* = 3.7 Hz, 0.6H), 5.03 – 4.95 (m, 1H), 4.95 – 4.72 (m, 16H), 4.65 (d, *J* = 12.0 Hz, 1H), 4.61 – 4.52 (m, 8H), 4.51 – 4.44 (m, 6H), 4.42 – 4.36 (m, 8H), 4.08 (dt, *J* = 9.8, 2.8 Hz, 1H), 4.03 – 3.85 (m, 13H), 3.81 – 3.53 (m, 18H), 3.50 – 3.28 (m, 32H), 3.27 – 3.22 (m, 2H), 3.19 – 3.08 (m, 10H). ^{13}C NMR (151 MHz, $CDCl_3$) δ 139.23, 139.21, 139.17, 139.11, 139.08, 138.93, 138.86, 138.77, 137.76, 137.52, 137.46, 137.35, 137.32, 137.28, 137.25, 137.23, 137.20, 129.52, 128.49, 128.47, 128.41, 128.37, 128.33, 128.30, 128.27, 128.20, 128.16, 128.11, 128.09, 128.07, 128.04, 128.00, 127.99, 127.96, 127.95, 127.89, 127.76, 127.70, 127.56, 127.38, 127.25, 127.23, 127.21, 127.17, 127.11, 126.84, 126.80, 126.66, 126.58, 126.55, 103.82, 103.72, 103.57, 103.36, 103.12, 96.82, 92.29, 83.51, 83.40, 83.08, 81.07, 77.27, 77.19, 77.06, 76.84, 76.71, 76.65, 75.81, 75.78, 75.71, 75.55, 75.41, 75.18, 74.75, 74.57, 74.41, 74.38, 74.31, 74.21, 74.17, 74.10, 74.06, 73.67, 73.61, 73.57, 73.55, 73.52, 72.33, 71.64, 70.43, 70.34, 68.59, 68.46, 67.78. (MALDI-TOF) m/z 3119.9 [M+Na]$^+$ ($C_{180}H_{200}O_{46}Na$ requires 3120.3).

^{1}H NMR of P-D$_9$ (600 MHz, $CDCl_3$)

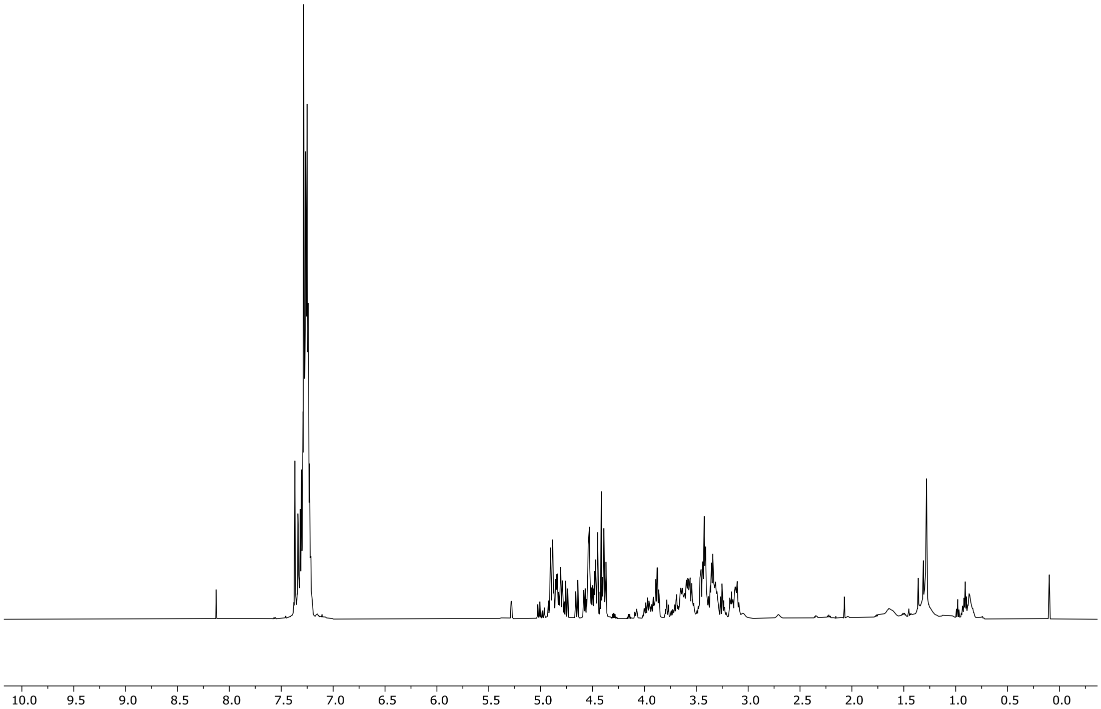

^{13}C NMR of P-D$_9$ (151 MHz, $CDCl_3$)

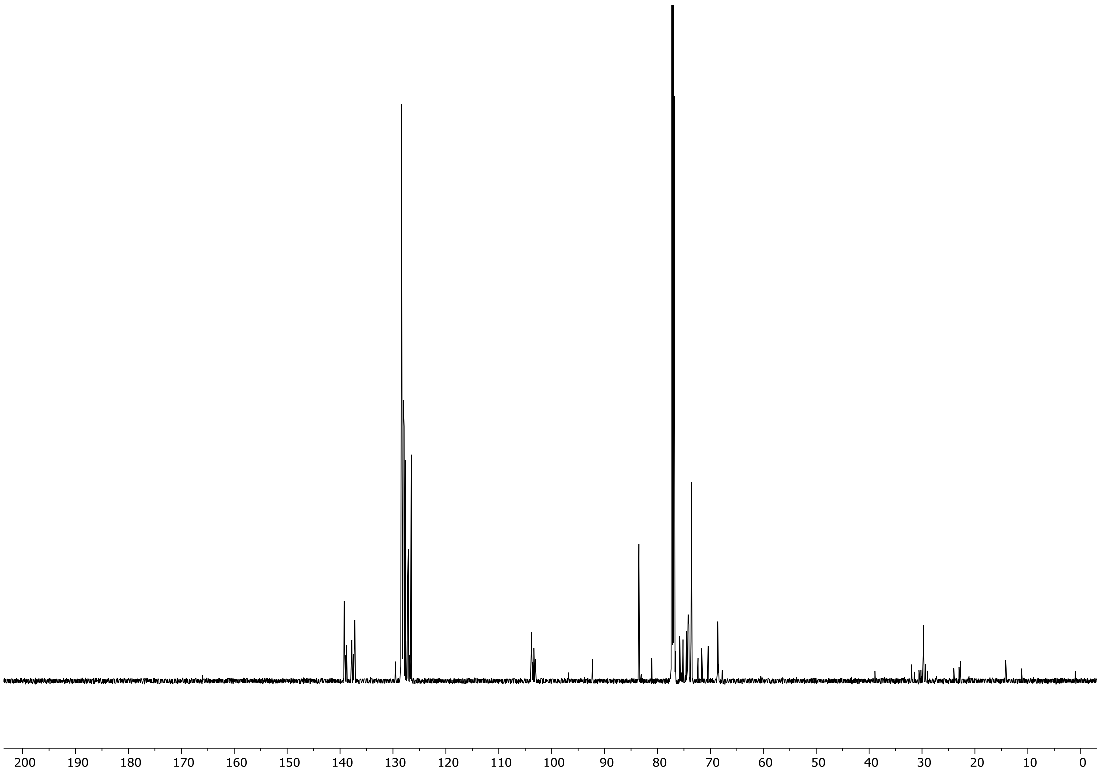

HSQC NMR of P-D$_9$ ($CDCl_3$)

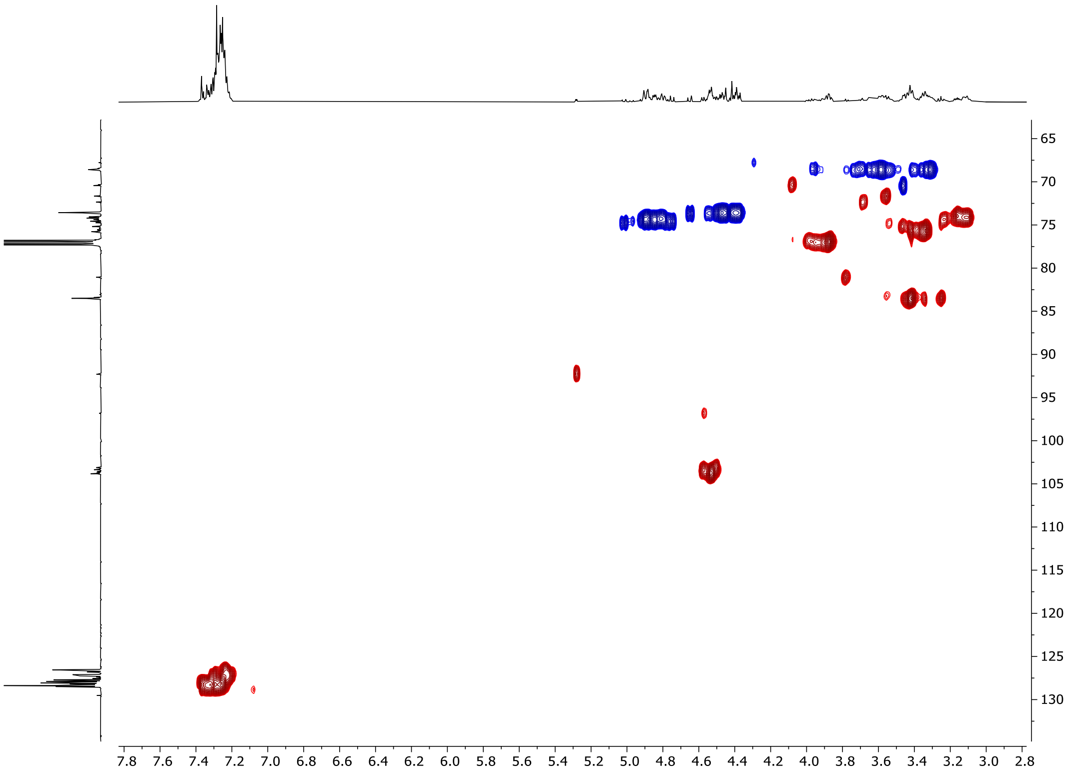

Analytical data for **D_9**:

^{1}H NMR (600 MHz, D_2O) δ 5.11 (d, *J* = 3.7 Hz, 0.4H), 4.55 (d, *J* = 7.1 Hz, 0.6H), 4.44 – 4.38 (m, 8H), 4.06 – 3.97 (m, 1H), 3.91 – 3.79 (m, 12H), 3.72 (dd, *J* = 12.3, 4.8 Hz, 10H), 3.65 – 3.45 (m, 20H), 3.41 – 3.35 (m, 3H), 3.30 (t, *J* = 9.4 Hz, 1H), 3.27-3.22 (m, 6H), 3.22 – 3.14 (m, 1H). (ESI-HRMS) m/z 1499.485 $[M+Na]^+$ ($C_{54}H_{92}O_{46}Na$ requires 1499.475).

^{1}H NMR of D_9 (400 MHz, D_2O)

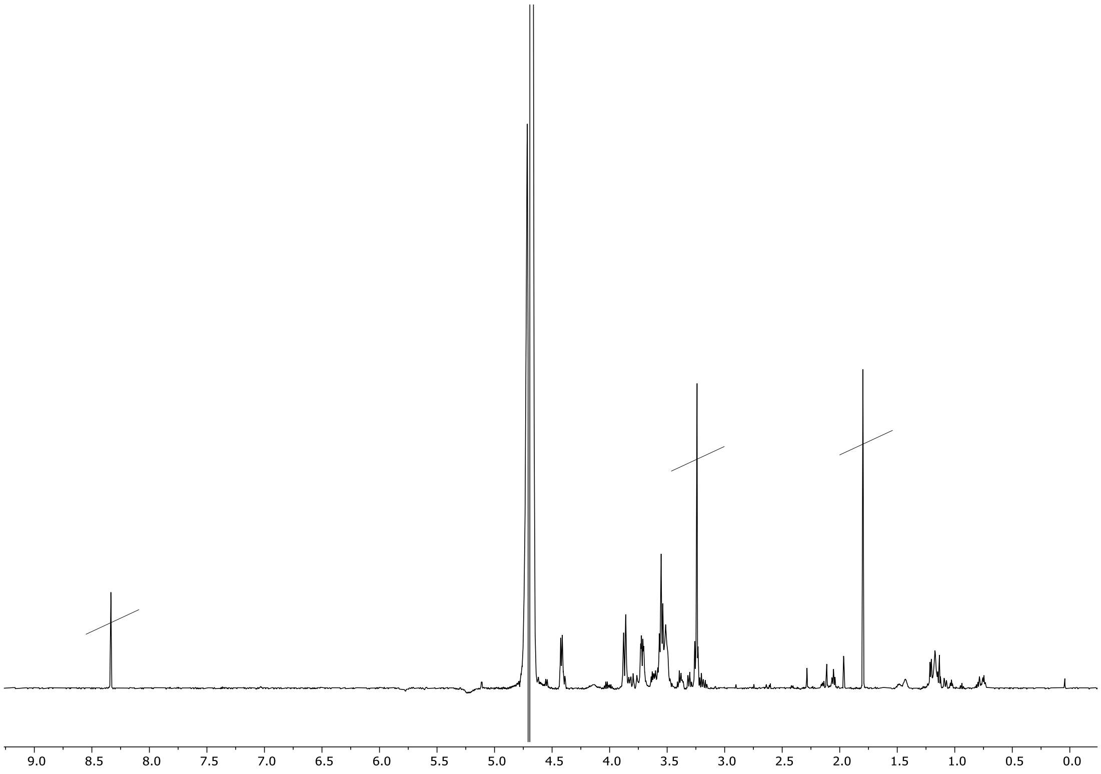

3.5.6 L_6

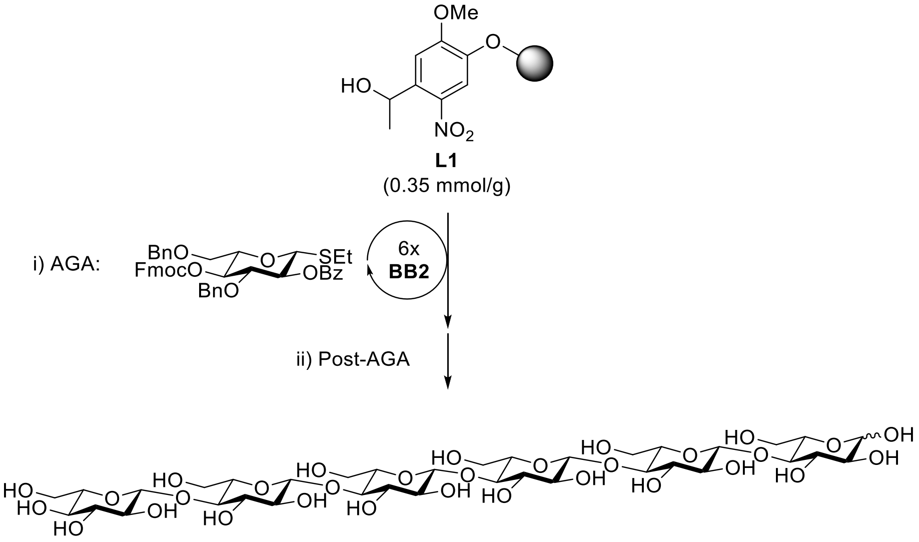

L_6

Step	Modules		Notes
AGA	**L1**	**A**	**L1** swelling
	(**BB2**)x6	(**B**, **C1**, **D**, **E**)x6	**C1**: (**BB2**, -20 °C for 5 min, 0 °C for 20 min)
Post-AGA		**F**, **G**, **H2**, **I**	**F**: (3 d)<br>**H2**: (6 h)<br>**I**: (Method C)

Automated synthesis, global deprotection, and purification afforded compound **L_6** as a white solid (8.4 mg, 60% overall yield).

Analytical data for **L_6**:
^{1}H NMR (600 MHz, D_2O) δ 5.24 (d, J = 3.8 Hz, 0.4H, $H1^{I}$-α), 4.68 (d, J = 7.9 Hz, 0.6H, $H1^{I}$-β), 4.59 – 4.50 (m, 5H, $H1^{II, III, IV, V, VI}$), 4.05 – 3.91 (m, 6H), 3.90 – 3.78 (m, 6H), 3.78 – 3.57 (m, 16H), 3.55 – 3.47 (m, 2H), 3.47 – 3.41 (m, 1H), 3.40 – 3.35 (m, 4H), 3.35 – 3.28 (m, 1H). ^{13}C NMR (151 MHz, D_2O) δ 102.48, 102.27, 95.68 (s, β-C1), 91.75 (s, α-C1), 78.56, 78.41, 78.31, 78.18, 75.91, 75.41, 74.75, 74.18, 73.96, 73.92, 73.82, 73.07, 72.85, 71.23, 71.16, 70.05, 69.37, 60.49, 59.94, 59.78. (ESI-HRMS) m/z 1013.322 $[M+Na]^+$ ($C_{36}H_{62}O_{31}Na$ requires 1013.317).

RP-HPLC of L_6 (ELSD trace, Method A1, t_R = 15.0 min)

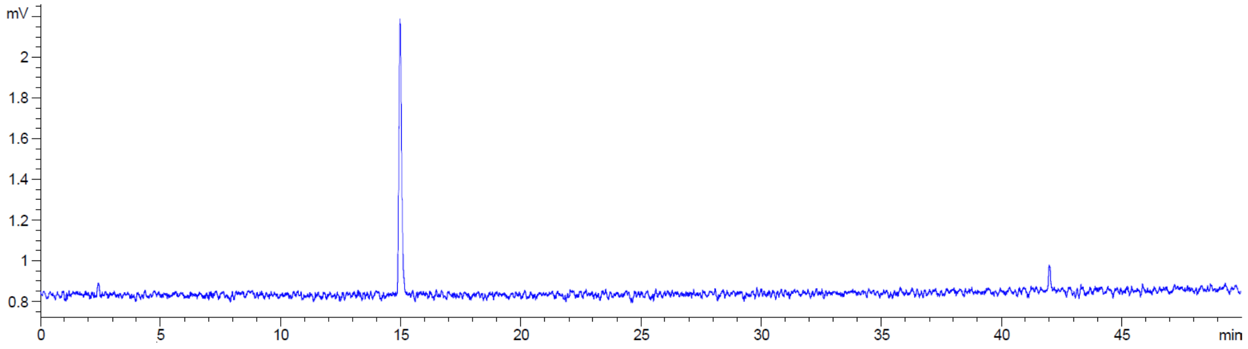

^{1}H NMR of L_6 (600 MHz, D_2O)

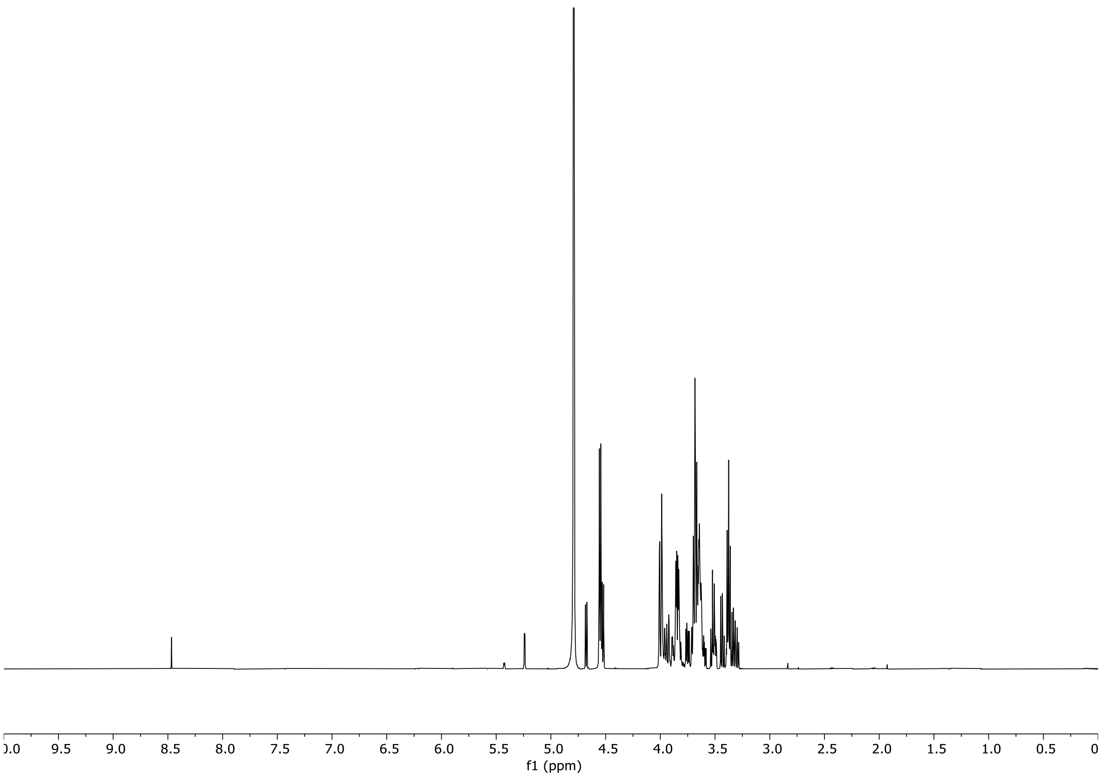

^{13}C NMR of L_6 (151 MHz, D_2O)

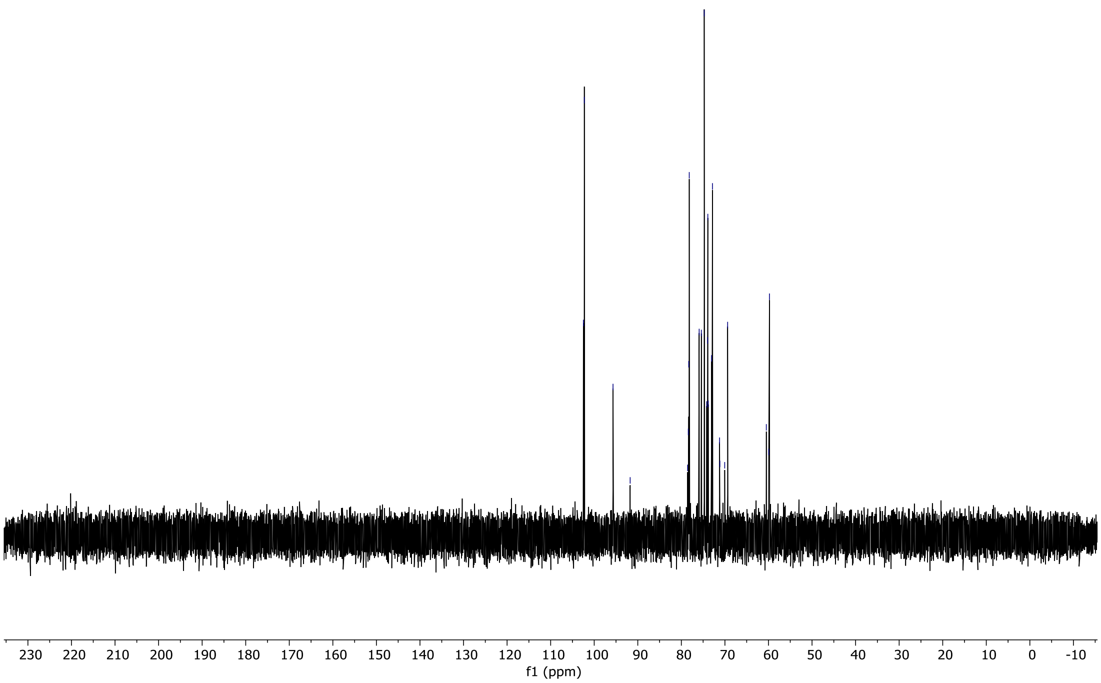

HSQC NMR of L_6 (D_2O)

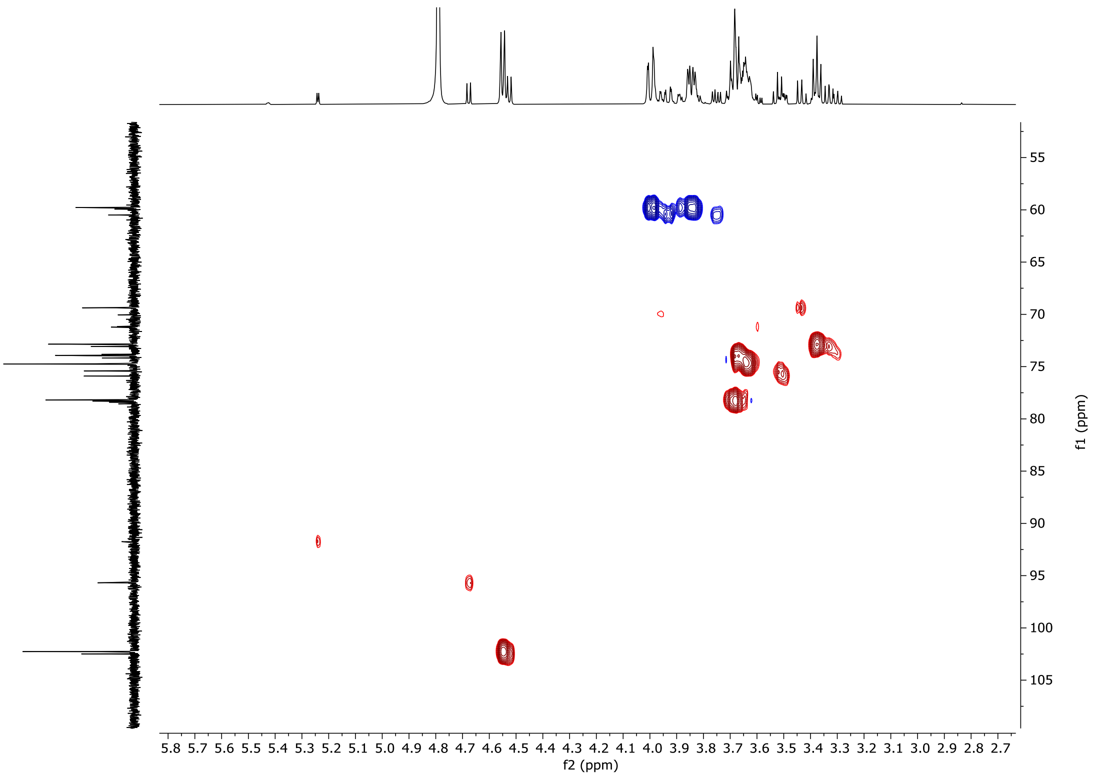

3.5.7 L_3D_3

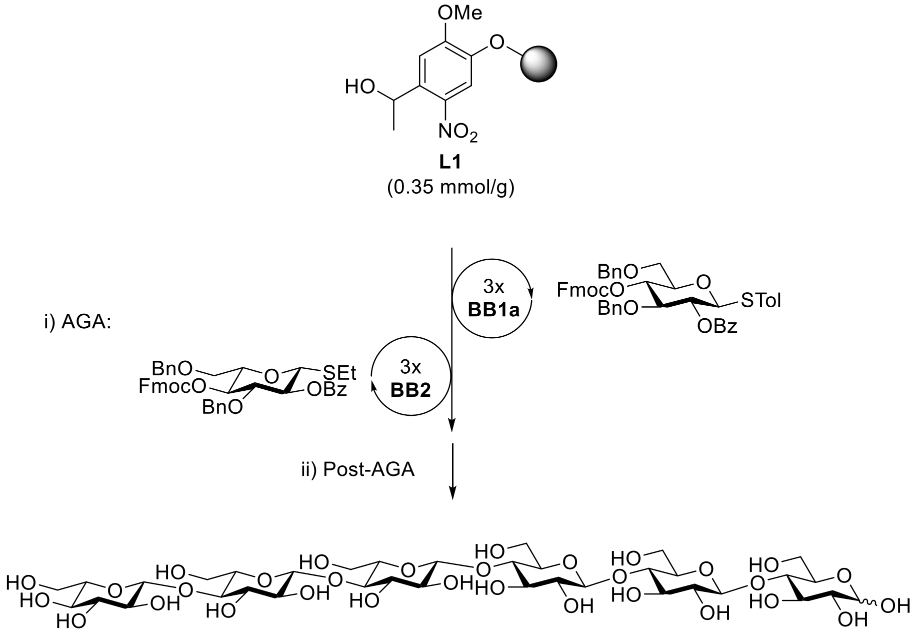

L_3D_3

Step	Modules		Notes
AGA	**L1**	**A**	**L1** swelling
	(**BB1a**)x3	(**B**, **C1**, **D**, **E**)x3	**C1**: (**BB1a**, -20 °C for 5 min, 0 °C for 20 min)
	(**BB2**)x3	(**B**, **C1**, **D**, **E**)x3	**C1**: (**BB2**, -20 °C for 5 min, 0 °C for 20 min)
Post-AGA		**F**, **G**, **H2**, **I**	**F**: (16 h)<br>**H2**: (4 h)<br>**I**: (Method C)

Automated synthesis, global deprotection, and purification afforded compound **L_3D_3** as a white solid (5.3 mg, 37% overall yield).

Analytical data for **L_3D_3**:
^{1}H NMR (400 MHz, D_2O) δ 5.15 (d, *J* = 3.7 Hz, 0.4H, H1^{I}-α), 4.70 (d, *J* = 7.9 Hz, 1H, H1^{IV}), 4.59 (d, *J* = 8.0 Hz, 0.6H, H1^{I}-β), 4.50 – 4.40 (m, 4H, H1$^{II, III, V, VI}$), 3.97 – 3.71 (m, 12H), 3.71 – 3.63 (m, 3H), 3.63 – 3.45 (m, 12H), 3.45 – 3.38 (m, 2H), 3.35 (d, *J* = 9.1 Hz, 1H), 3.33 – 3.17 (m, 6H). ^{13}C NMR (101 MHz, D_2O) δ 102.67, 102.44, 102.30, 102.22, 95.63, 91.69, 76.43, 75.85, 75.32, 75.07, 74.69, 74.51, 74.33, 74.10, 74.03, 73.88, 73.85, 73.75, 73.17, 73.00, 72.93, 72.80, 71.16, 69.99, 69.30, 60.41, 60.13, 59.89, 59.82, 59.71. (ESI-HRMS) m/z 1013.324 $[M+Na]^+$ ($C_{36}H_{62}O_{31}Na$ requires 1013.317).

RP-HPLC of L_3D_3 (ELSD trace, Method A1, t_R = 15.4 min)

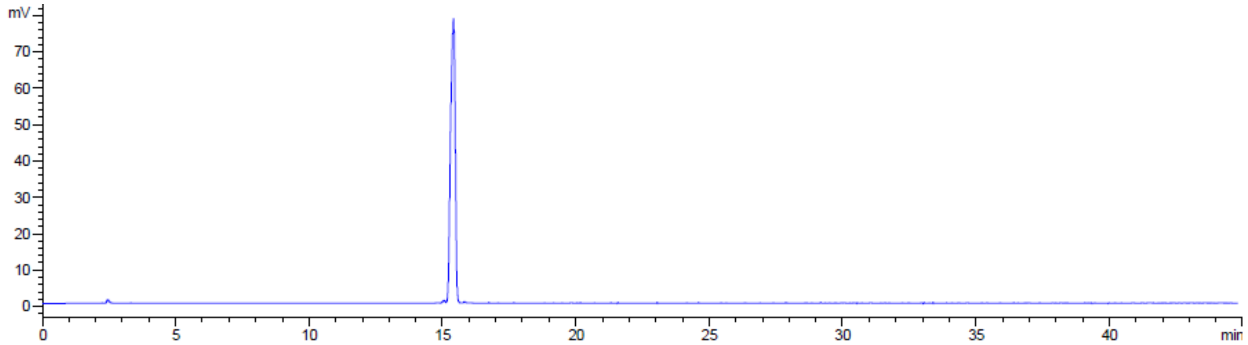

^{1}H NMR of L_3D_3 (400 MHz, D_2O)

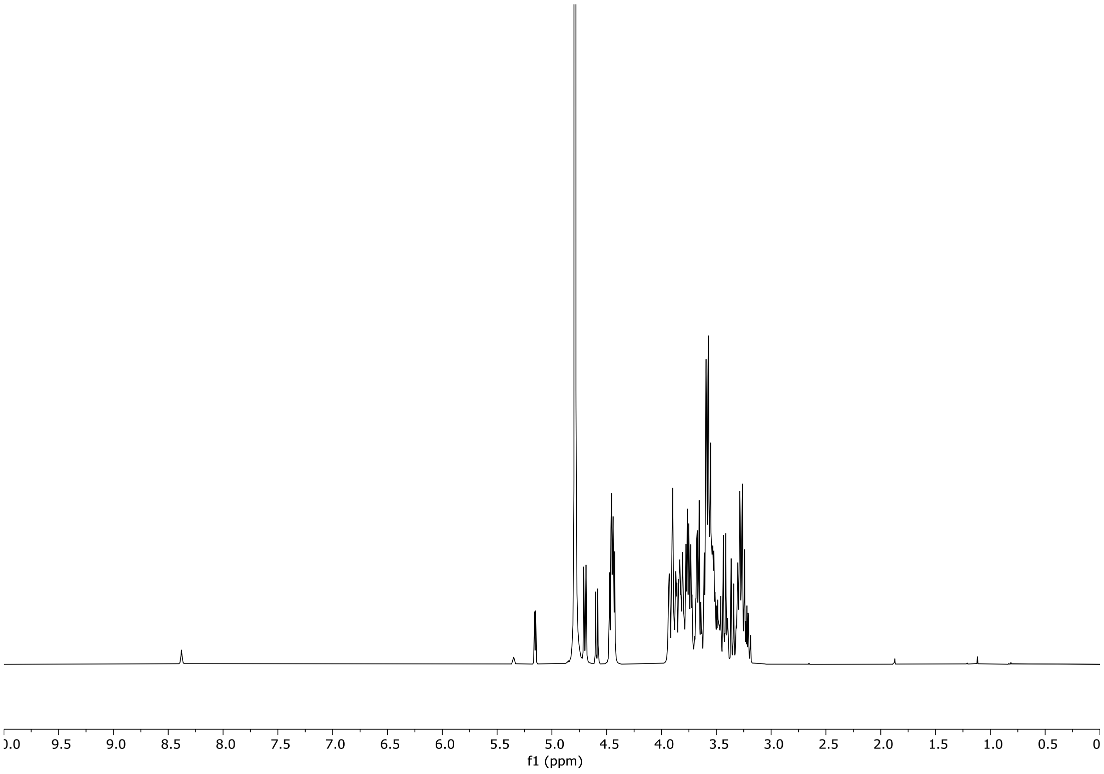

^{13}C NMR of L_3D_3 (101 MHz, D_2O)

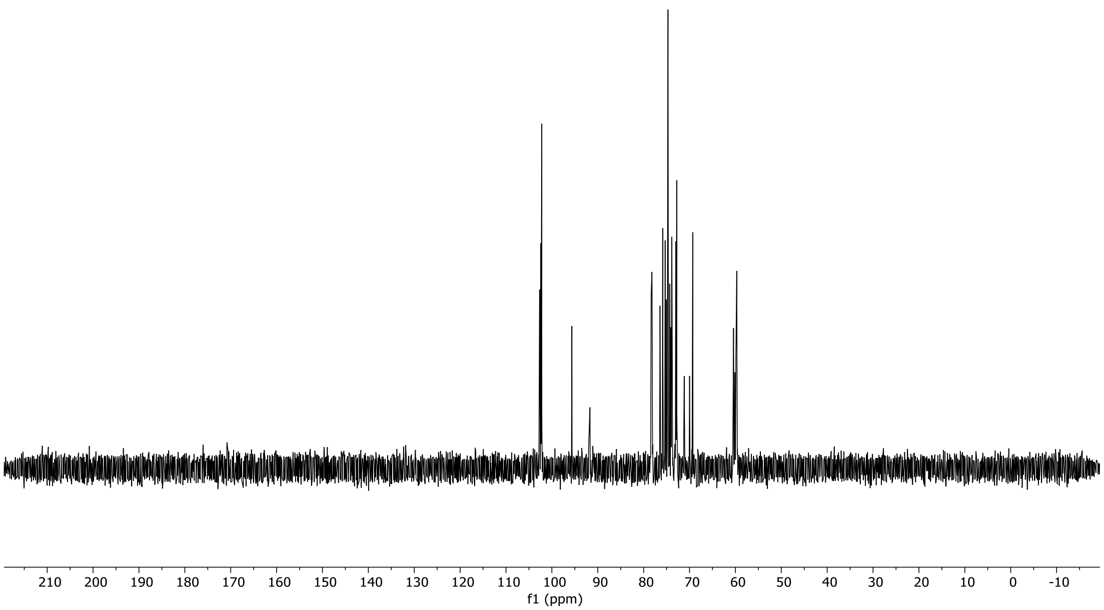

HSQC NMR of L_3D_3 (D_2O)

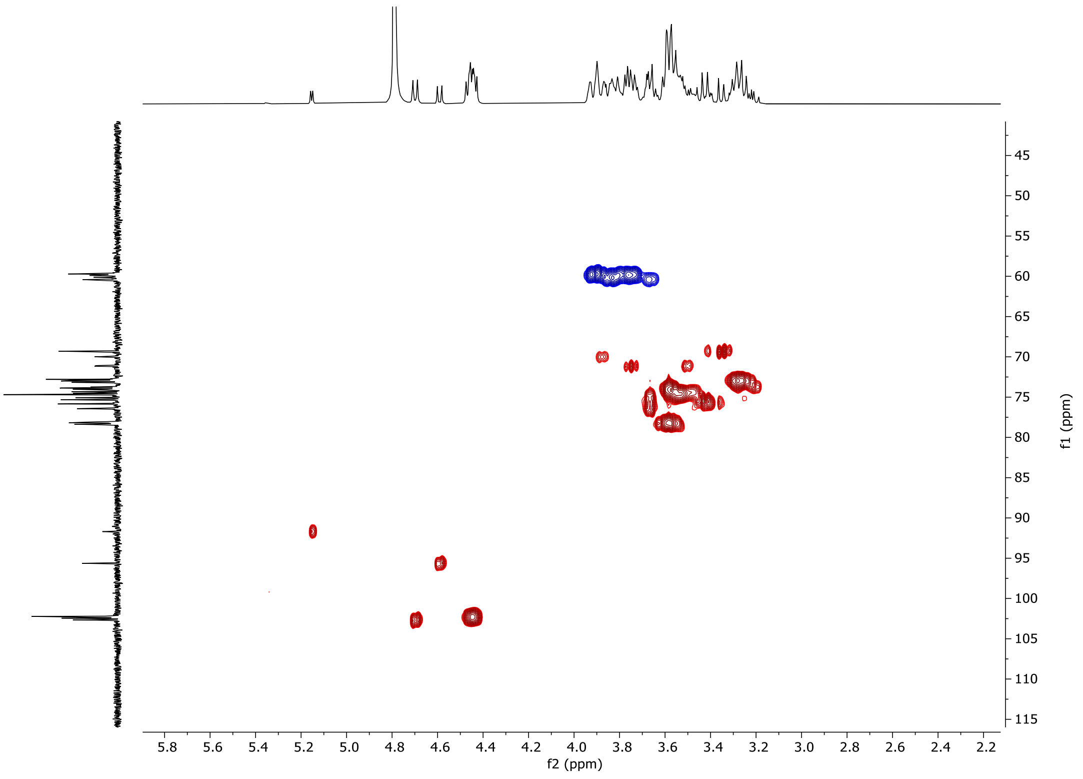

3.5.8 L_2D_4

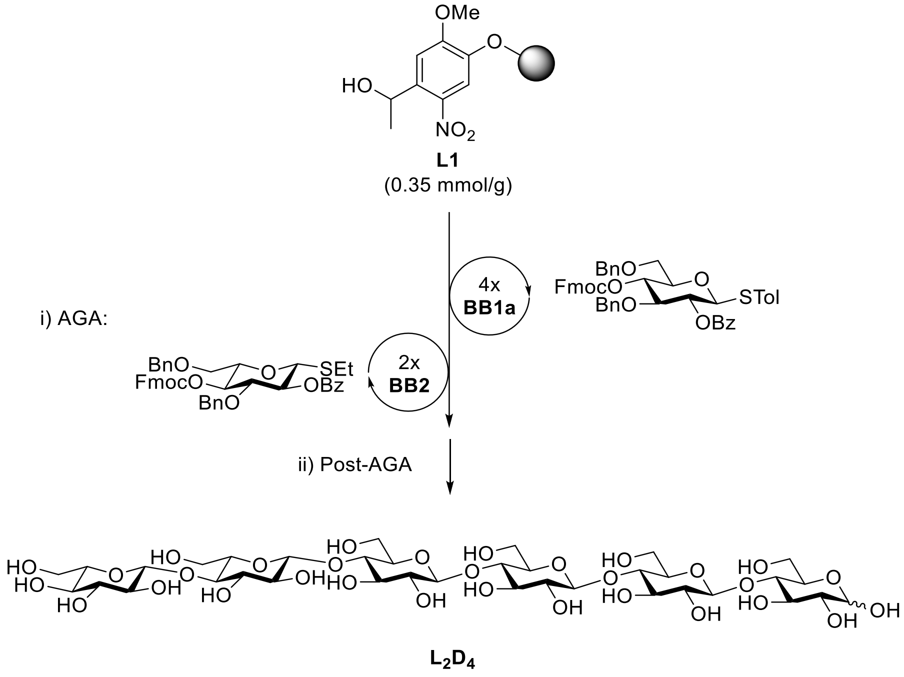

Step	Modules		Notes
AGA	**L1**	**A**	**L1** swelling
	(**BB1a**)x4	(**B**, **C1**, **D**, **E**)x4	**C1**: (**BB1a**, -20 °C for 5 min, 0 °C for 20 min)
	(**BB2**)x2	(**B**, **C1**, **D**, **E**)x2	**C1**: (**BB2**, -20 °C for 5 min, 0 °C for 20 min)
Post-AGA		**F**, **G**, **H2**, **I**	**F**: (16 h)<br>**H2**: (4 h)<br>**I**: (Method C)

Automated synthesis, global deprotection, and purification afforded compound $\mathbf{L_3D_3}$ as a white solid (2.8 mg, 23% overall yield).

Analytical data for $\mathbf{L_2D_4}$:
^{1}H NMR (600 MHz, D_2O) δ 5.18 (d, *J* = 3.7 Hz, 0.4H, $H1^{I}$-α), 4.72 (d, *J* = 7.9 Hz, 1H, $H1^{V}$), 4.62 (d, *J* = 8.0 Hz, 0.6H, $H1^{I}$-β), 4.52 – 4.44 (m, 4H, $H1^{II, III, IV, VI}$), 3.98 – 3.73 (m, 12H), 3.72 – 3.66 (m, 3H), 3.66 – 3.41 (m, 15H), 3.39 – 3.34 (m, 1H), 3.34 – 3.20 (m, 5H). ^{13}C NMR (151 MHz, D_2O) δ 102.73, 102.51, 102.36, 102.30, 95.70, 91.77, 78.61, 78.55, 78.40, 78.22, 78.17, 76.55, 75.93, 75.41, 75.16, 74.76, 74.58, 74.42, 74.19, 74.16, 73.94. 73.84, 73.25, 73.09, 73.00, 72.89, 72.87, 71.24, 71.17, 71.07, 69.39, 60.51, 60.22, 60.05, 59.94, 59.80. (ESI-HRMS) m/z 1013.323 [M+Na]$^+$ ($C_{36}H_{62}O_{31}Na$ requires 1013.317).

RP-HPLC of L_2D_4 (ELSD trace, Method A1, t_R = 14.6, 14.7 min)

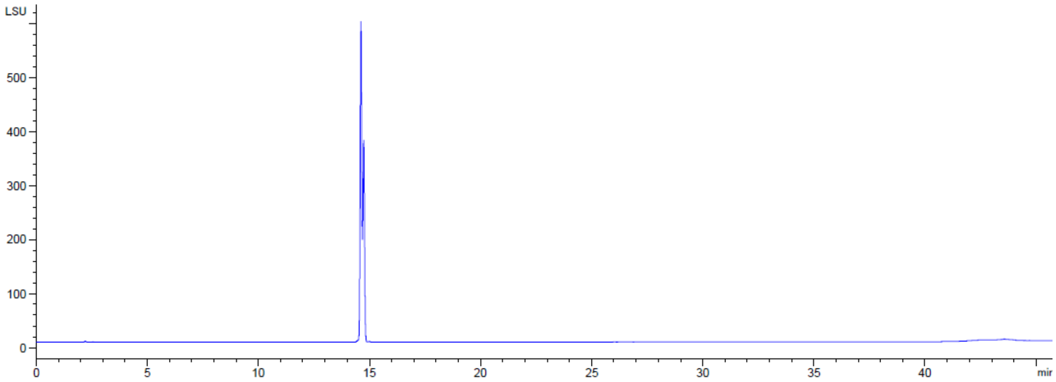

^{1}H NMR of L_2D_4 (400 MHz, D_2O)

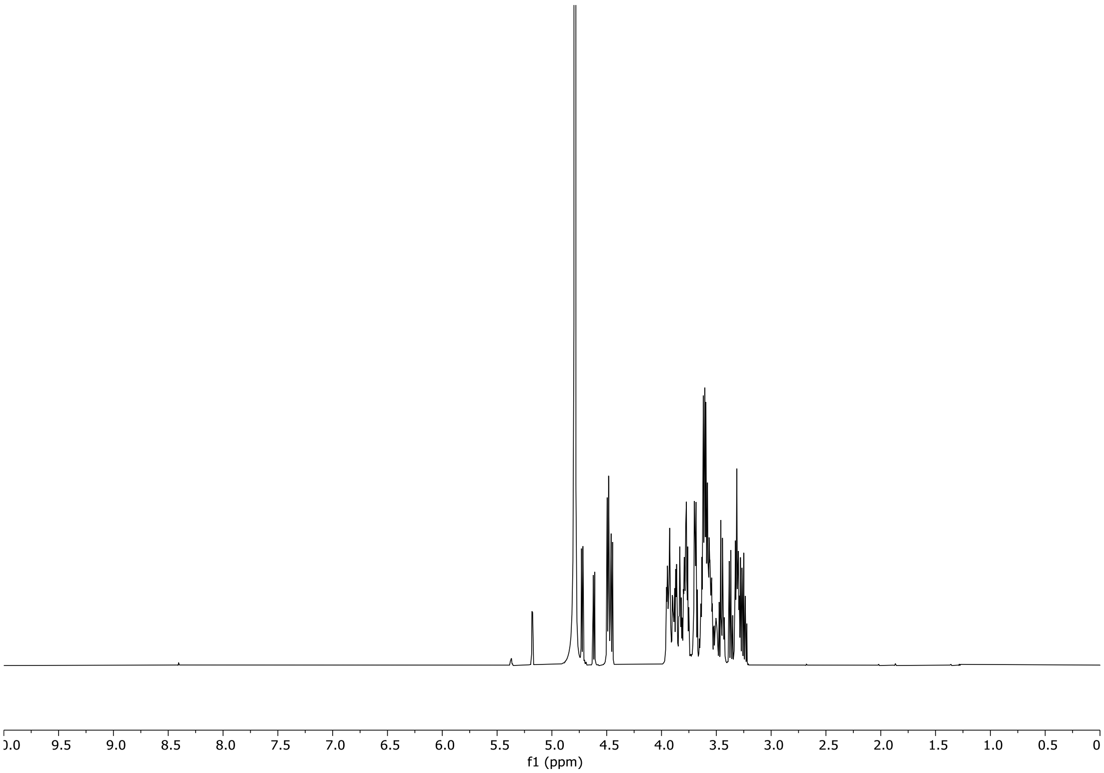

^{13}C NMR of L_2D_4 (101 MHz, D_2O)

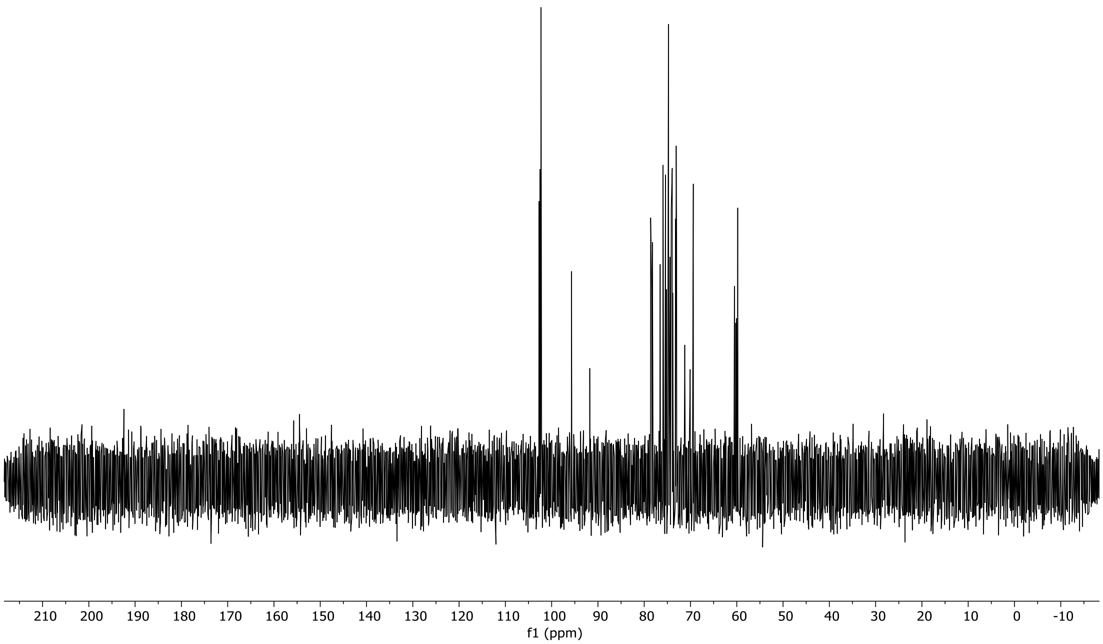

HSQC NMR of L_2D_4 (D_2O)

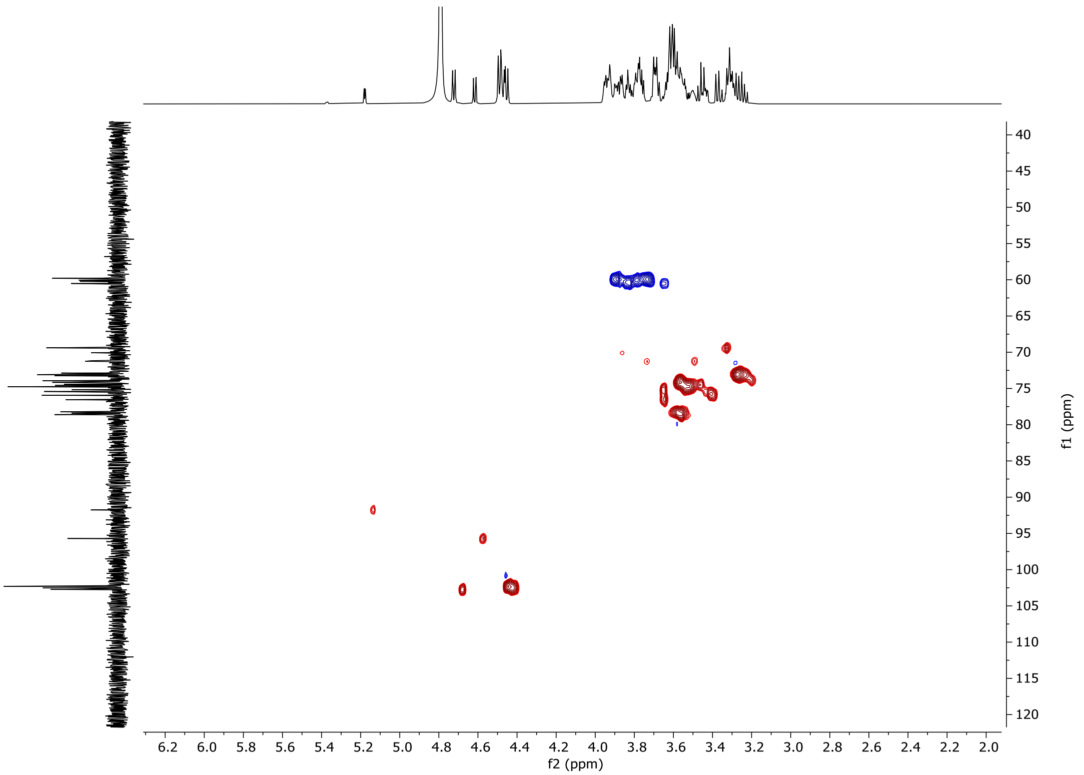

3.5.9 LD_6L

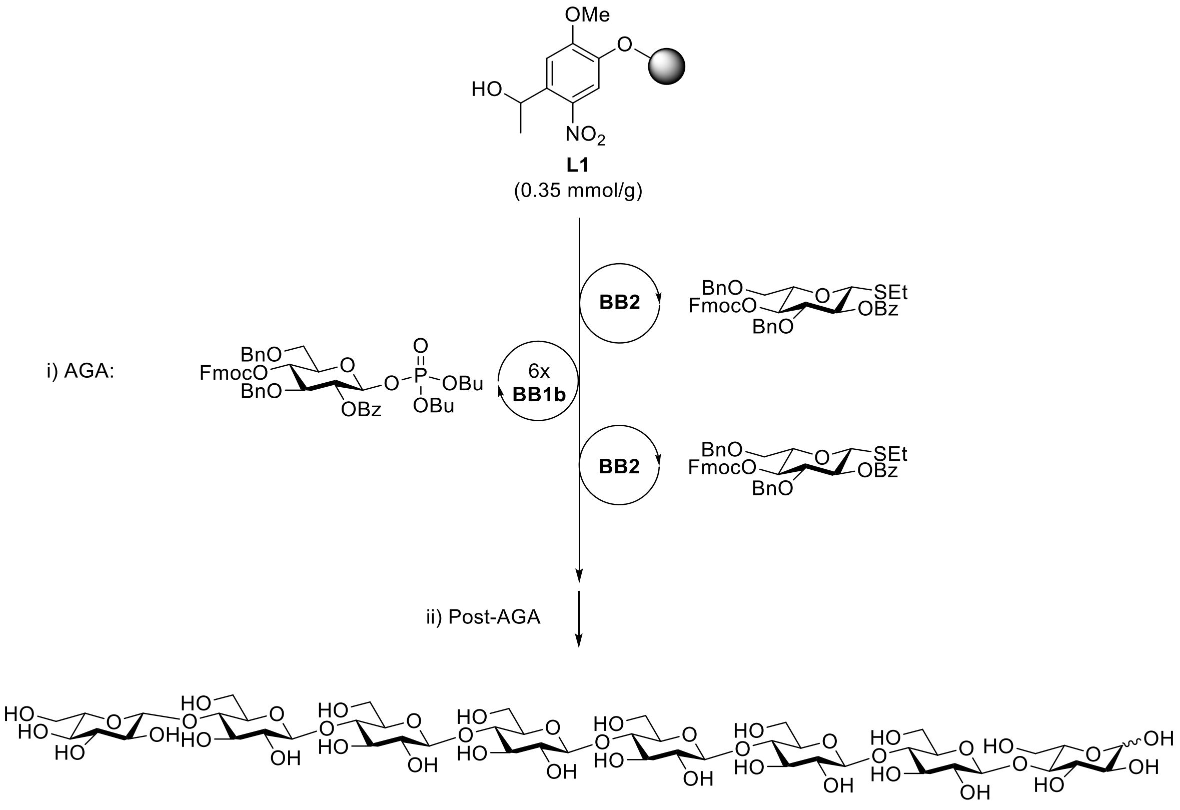

LD_6L

Step	Modules		Notes
AGA	**L1**	**A**	**L1** swelling
	**BB2**	**B**, **C1**, **D**, **E**	**C1**: (**BB2**, -20 °C for 5 min, 0 °C for 20 min)
	(**BB1b**)x6	(**B**, **C2**, **D**, **E**)x6	**C2**: (**BB1b**, -35 °C for 5 min, -15 °C for 20 min)
	**BB2**	**B**, **C1**, **D**, **E**	**C1**: (**BB2**, -20 °C for 5 min, 0 °C for 20 min)
Post-AGA		**F**, **G**, **H2**	**F**: (3 d)<br>**H2**: (4 h)<br>**I**: (Method C)

Automated synthesis, global deprotection afforded compound **LD_6L** as a white solid (8.5 mg, 50% overall yield).

Analytical data for **LD_6L**:
f^{1}H NMR (600 MHz, D_2O) δ 5.18 (d, *J* = 3.7 Hz, 0.4H, $H1^{I}$-α), 4.72 (d, *J* = 8.0 Hz, 1H, $H1^{II}$), 4.69 (d, *J* = 7.9 Hz, 1H, , $H1^{VIII}$), 4.59 (d, *J* = 8.0 Hz, 0.6H, $H1^{I}$-β), 4.51 – 4.43 (m, 5H, $H1^{III, IV, V, VI, VII}$), 3.93 (d, *J* = 10.8 Hz, 5H), 3.90 – 3.73 (m, 11H), 3.73 – 3.52 (m, 19H), 3.52 – 3.37 (m, 5H), 3.37 – 3.19 (m, 8H). ^{13}C NMR (151 MHz, D_2O) δ 102.93, 102.71, 102.33, 102.26, 95.73, 91.86, 78.11, 76.66, 76.51, 75.65, 75.55, 75.40, 75.11, 74.72, 74.55, 74.53, 74.38, 74.34, 74.07, 73.88, 73.42, 73.22, 72.95, 72.83, 69.62, 60.68, 59.72. (ESI-HRMS) m/z 1337.427 $[M+Na]^+$ ($C_{48}H_{82}O_{41}Na$ requires 1337.422).

RP-HPLC of LD_6L (ELSD trace, Method A1, t_R = 15.7 min)

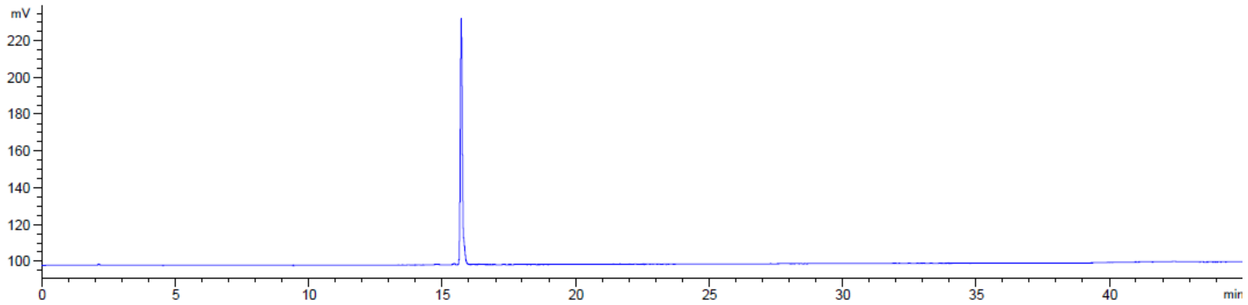

^1H NMR of LD_6L (400 MHz, D_2O)

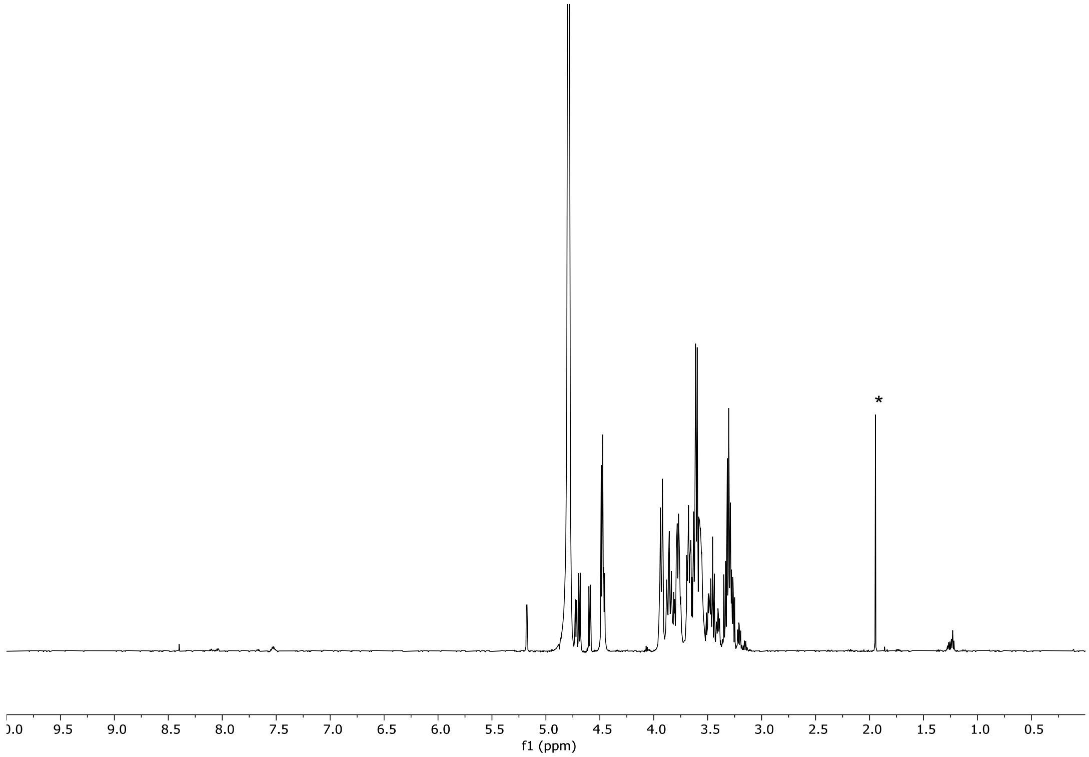

*Unidentified impurity

^{13}C NMR of LD_6L (101 MHz, D_2O)

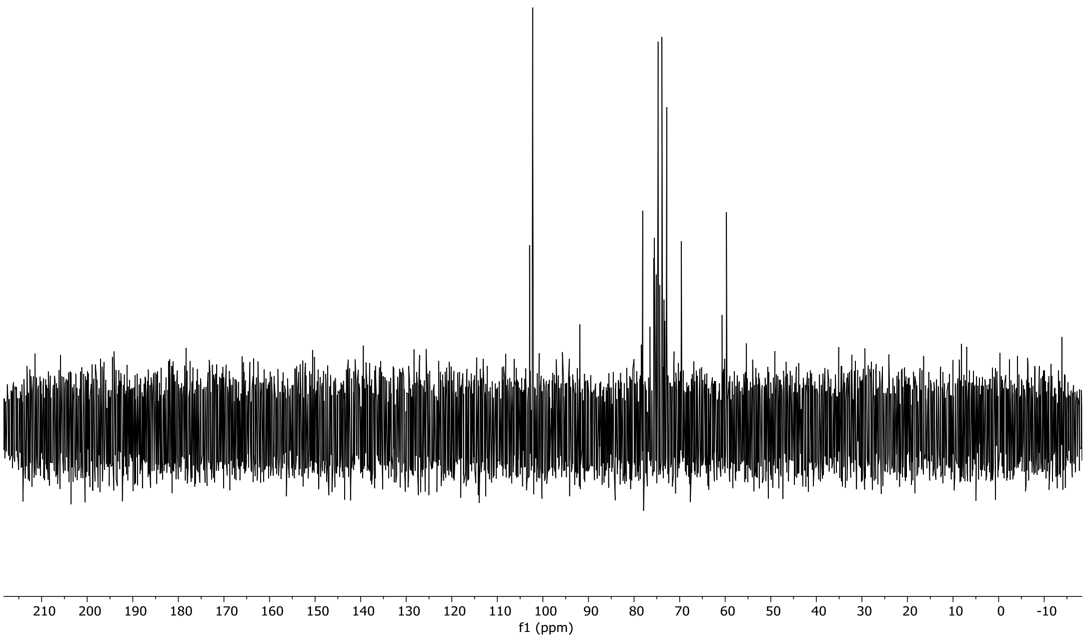

HSQC NMR of LD_6L (D_2O)

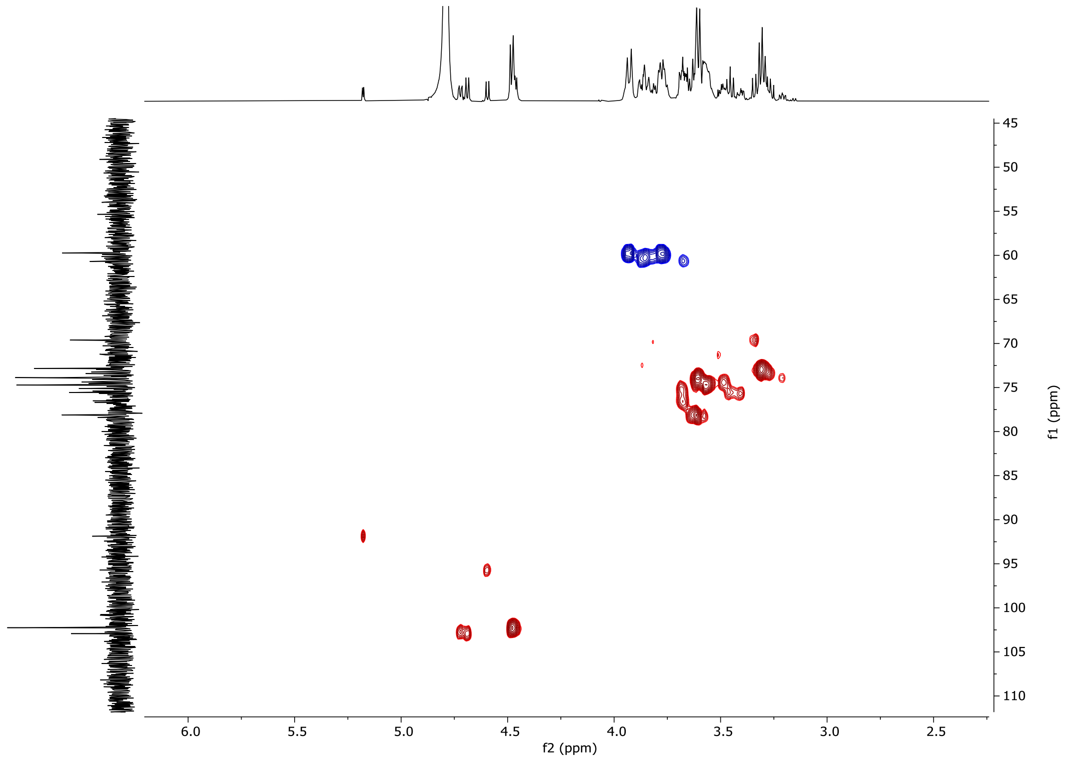

3.5.10 LD_5L

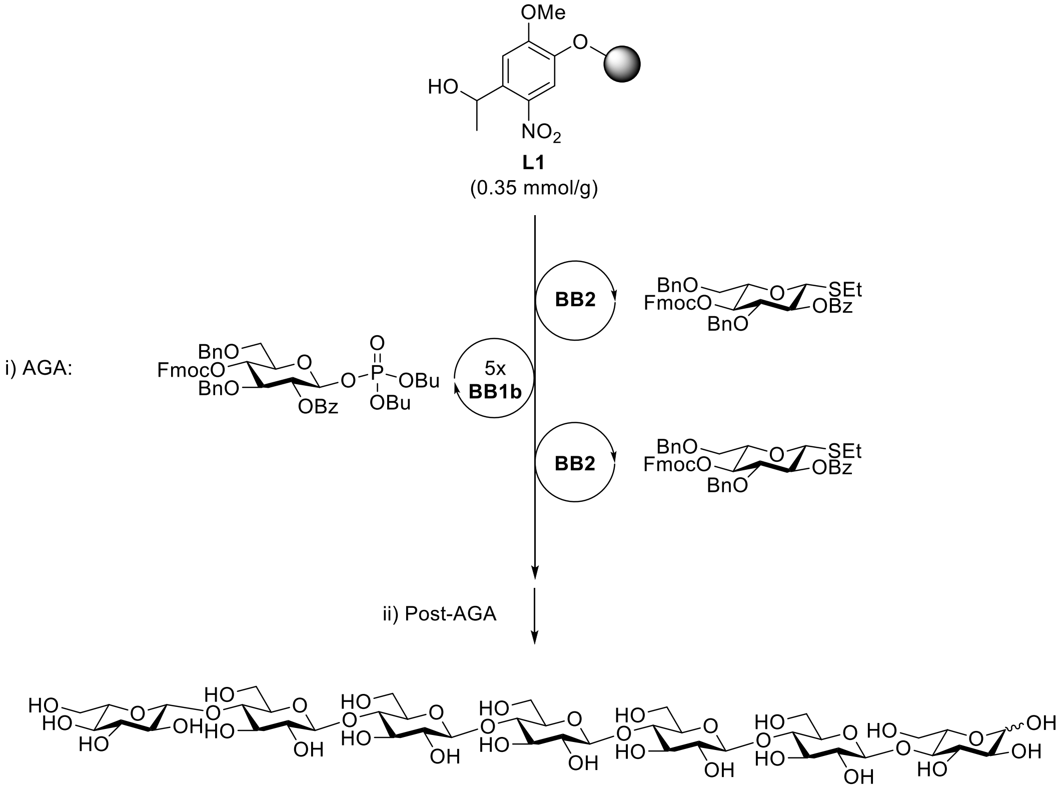

LD_5L

Step		Modules	Notes
AGA	**L1**	**A**	**L1** swelling
	**BB2**	**B**, **C1**, **D**, **E**	**C1**: (**BB2**, -20 °C for 5 min, 0 °C for 20 min)
	(**BB1b**)x5	(**B**, **C2**, **D**, **E**)x5	**C2**: (**BB1b**, -35 °C for 5 min, -15 °C for 20 min)
	**BB2**	**B**, **C1**, **D**, **E**	**C1**: (**BB2**, -20 °C for 5 min, 0 °C for 20 min)
Post-AGA		**F**, **G**, **H2**	**F**: (2 d)<br>**H2**: (5 h)<br>**I**: (Method C)

Automated synthesis, global deprotection afforded compound **LD_5L** as a white solid (5.1 mg, 35% overall yield).

Analytical data for **LD_5L**:
^{1}H NMR (600 MHz, D_2O) δ 5.21 (d, J = 3.7 Hz, 0.4H, $H1^{I}$-α), 4.75 (m, 1H, $H1^{II}$), 4.72 (d, J = 7.9 Hz, 1H, $H1^{VII}$), 4.63 (d, J = 8.0 Hz, 0.6H, $H1^{I}$-β), 4.54 – 4.47 (m, 4H, $H1^{III, IV, V, VI}$), 3.96 (d, J = 10.8 Hz, 4H), 3.93 – 3.76 (m, 10H), 3.76 – 3.55 (m, 16H), 3.55 – 3.40 (m, 4H), 3.40 – 3.27 (m, 7H), 3.27 – 3.20 (m, 1H). ^{13}C NMR (151 MHz, D_2O) δ 102.97, 102.76, 102.37, 102.30, 95.76, 91.84, 78.23, 78.18, 76.57, 75.70, 75.60, 75.45, 75.16, 74.76, 74.59, 74.43, 74.11, 73.93, 73.47, 73.27, 73.00, 72.87, 72.52, 71.27, 69.85, 69.66, 60.74, 60.38, 60.19, 59.98, 59.79. (ESI-HRMS) m/z 1175.381 $[M+Na]^+$ ($C_{42}H_{72}O_{36}Na$ requires 1175.370).

RP-HPLC of LD_5L (ELSD trace, Method A1, t_R = 15.5 min)

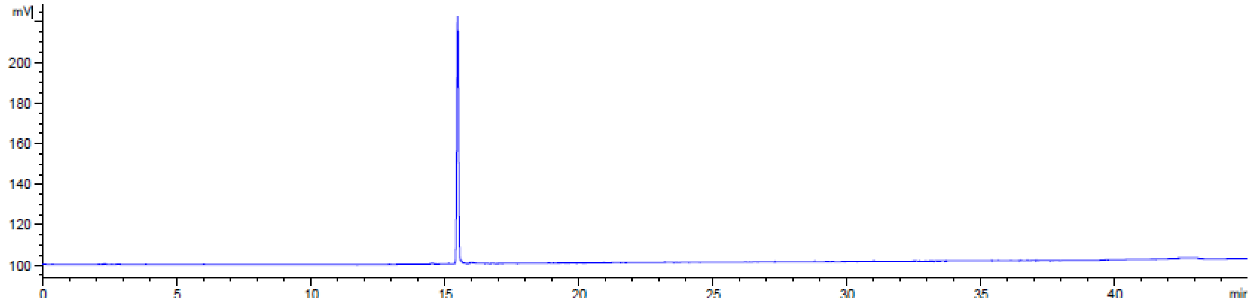

^1H NMR of LD_5L (400 MHz, D_2O)

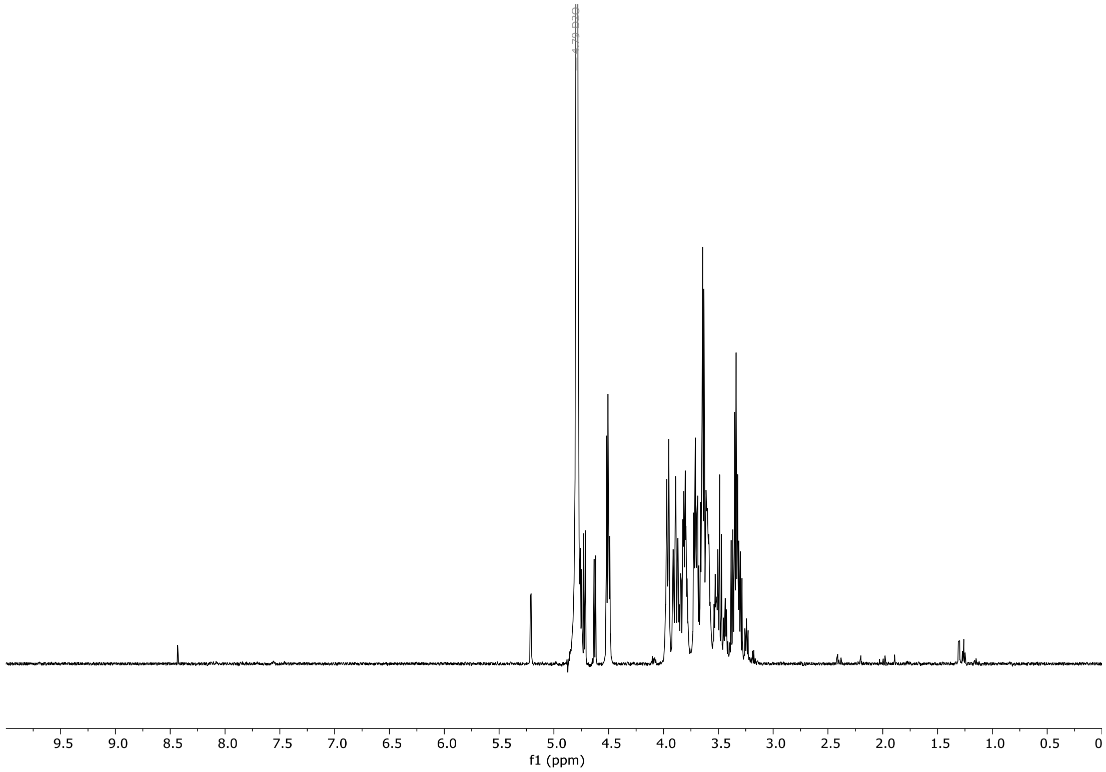

^{13}C NMR of LD_5L (101 MHz, D_2O)

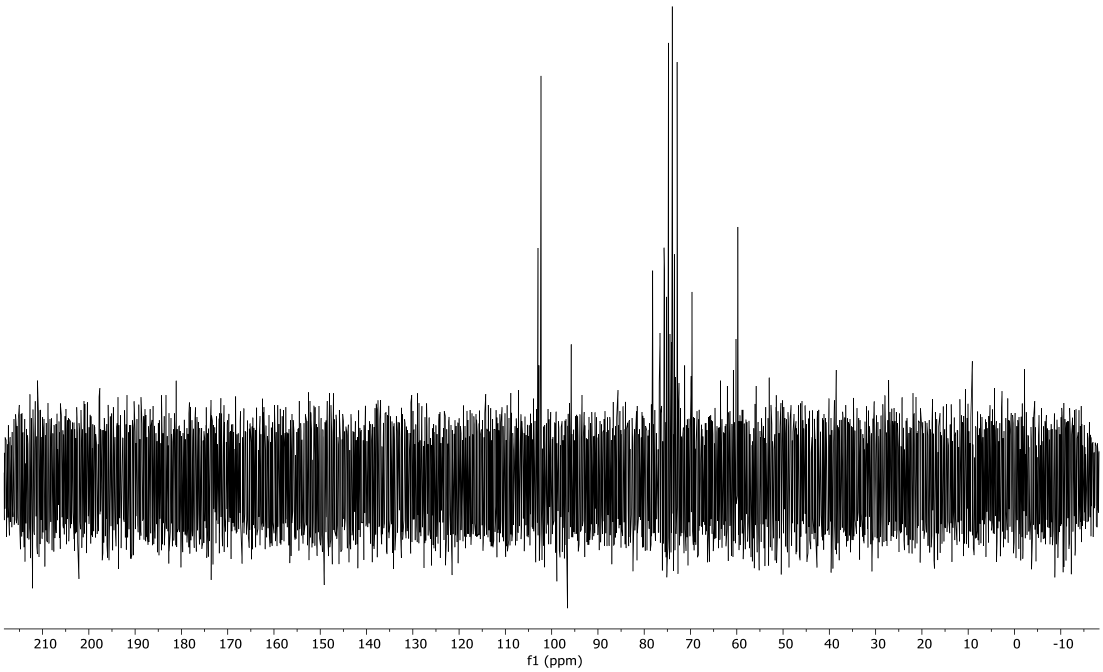

HSQC NMR of LD_5L (D_2O)

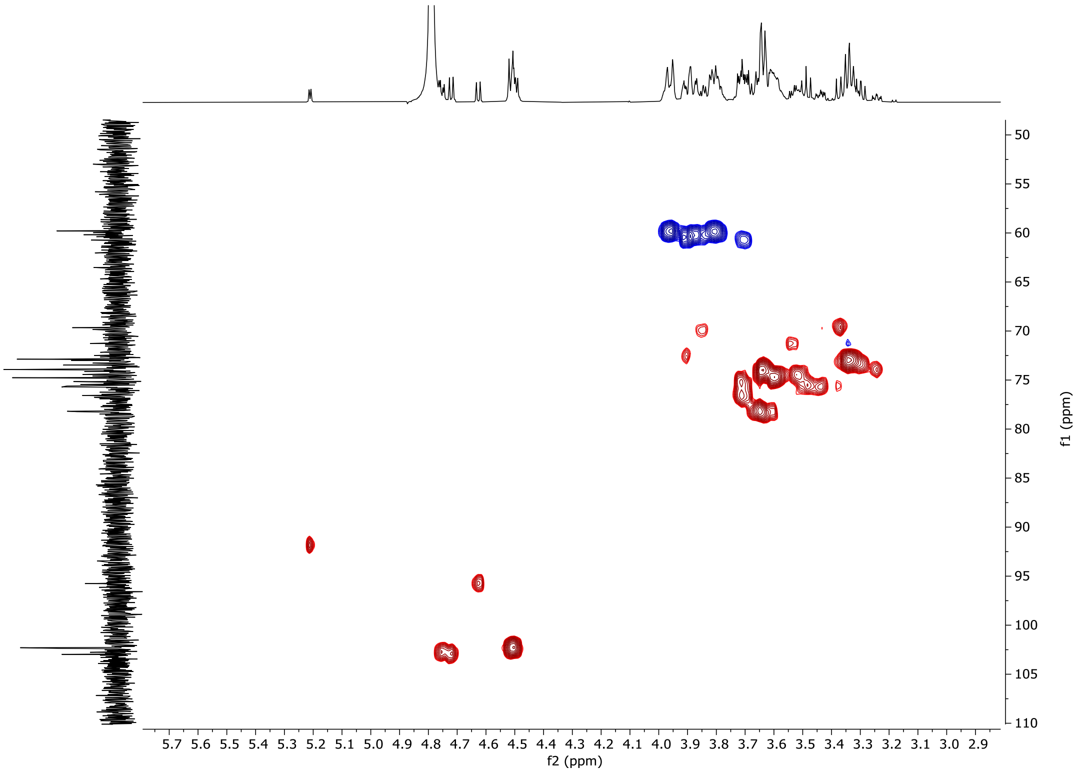

3.5.11 LD_6

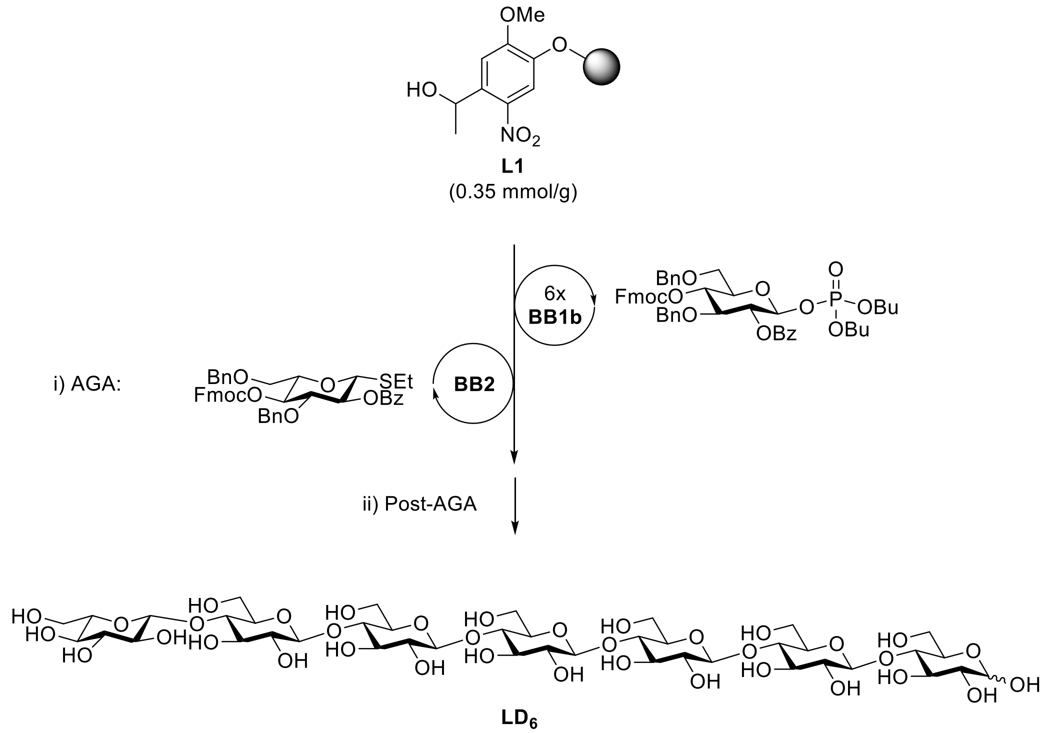

Step	Modules		Notes
AGA	**L1**	**A**	**L1** swelling
	(**BB1b**)x6	(**B**, **C2**, **D**, **E**)x6	**C2**: (**BB1b**, -35 °C for 5 min, -15 °C for 20 min)
	**BB2**	**B**, **C1**, **D**, **E**	**C1**: (**BB2**, -20 °C for 5 min, 0 °C for 20 min)
Post-AGA			**F**: (1 d)
		**F**, **G**, **H2**	**H2**: (4 h)
			**I**: (Method C)

Automated synthesis, global deprotection afforded compound **LD**$_6$ as a white solid (11.1 mg, 74% overall yield).

Analytical data for **LD**$_6$:
^{1}H NMR (600 MHz, D_2O) δ 5.12 (d, *J* = 3.8 Hz, 0.3H, H1^{I}-α), 4.63 (d, *J* = 8.0 Hz, 1H, H1^{VII}), 4.55 (d, *J* = 7.9 Hz, 0.7H, H1^{I}-β), 4.42 (dd, *J* = 10.7, 8.0 Hz, 5H, H1$^{II, III, IV, V, VI}$), 3.91 – 3.75 (m, 8H), 3.72 (dt, *J* = 15.8, 8.2 Hz, 5H), 3.66 – 3.60 (m, 3H), 3.60 – 3.47 (m, 15H), 3.45 (dd, *J* = 16.5, 3.3 Hz, 1H), 3.40 (t, *J* = 9.2 Hz, 1H), 3.35 (ddt, *J* = 12.3, 8.6, 4.2 Hz, 1H), 3.31 – 3.15 (m, 8H). ^{13}C NMR (151 MHz, D_2O) δ 102.97, 102.30, 95.70, 91.77, 78.54, 78.39, 78.18, 76.57, 75.70, 75.60, 75.16, 74.77, 74.43, 74.19, 73.93, 73.83, 73.47, 73.00, 72.87, 71.24, 71.17, 70.07, 69.66, 60.74, 60.18, 59.93, 59.78. (ESI-HRMS) m/z 1153.393 [M+H]$^+$ ($C_{42}H_{73}O_{36}$ requires 1153.388).

RP-HPLC of LD_6 (ELSD trace, Method A1, t_R = 14.8 min)

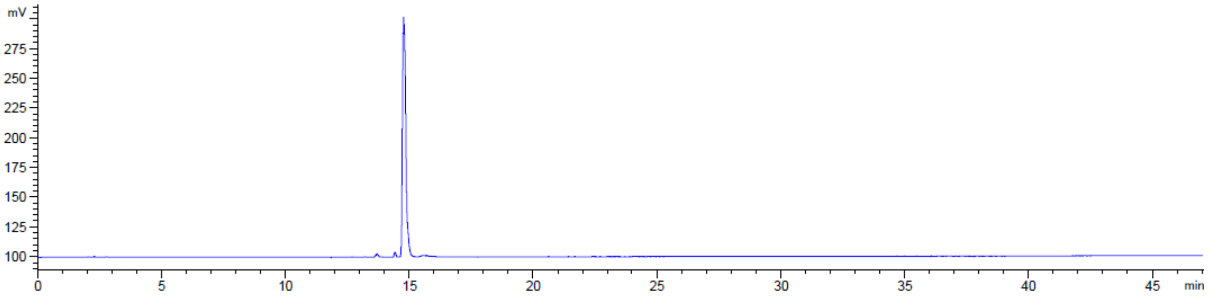

^{1}H NMR of LD_6 (600 MHz, D_2O)

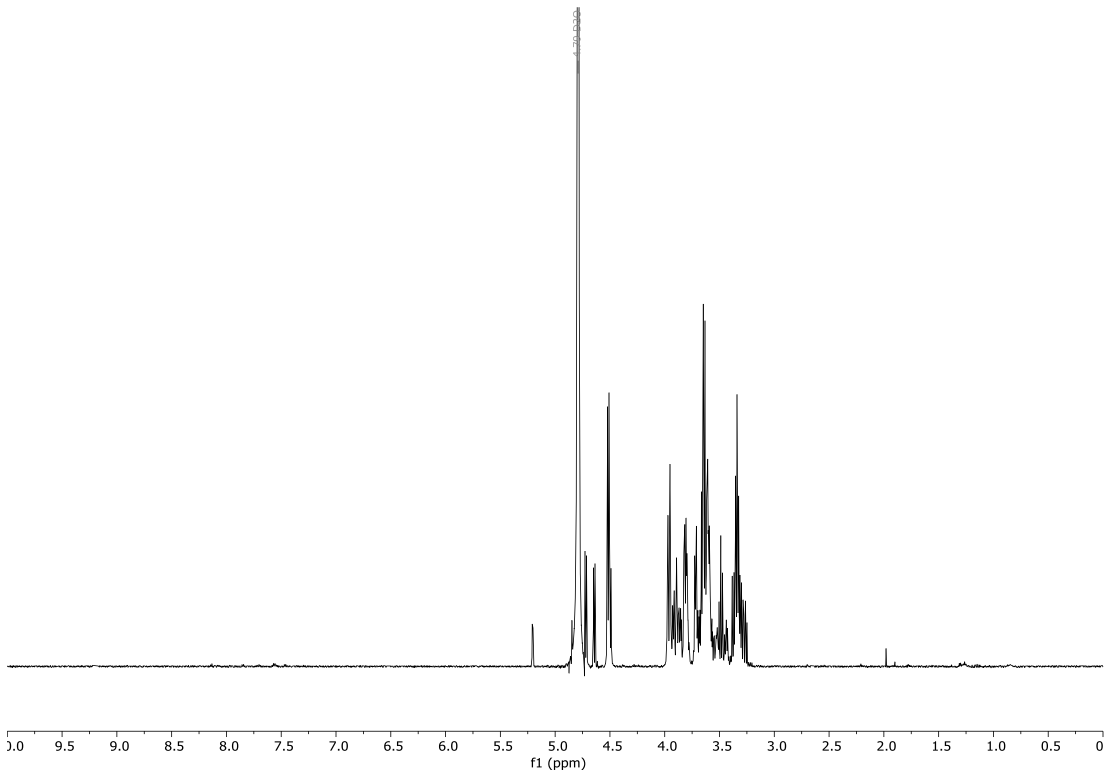

^{13}C NMR of LD$_6$ (151 MHz, D$_2$O)

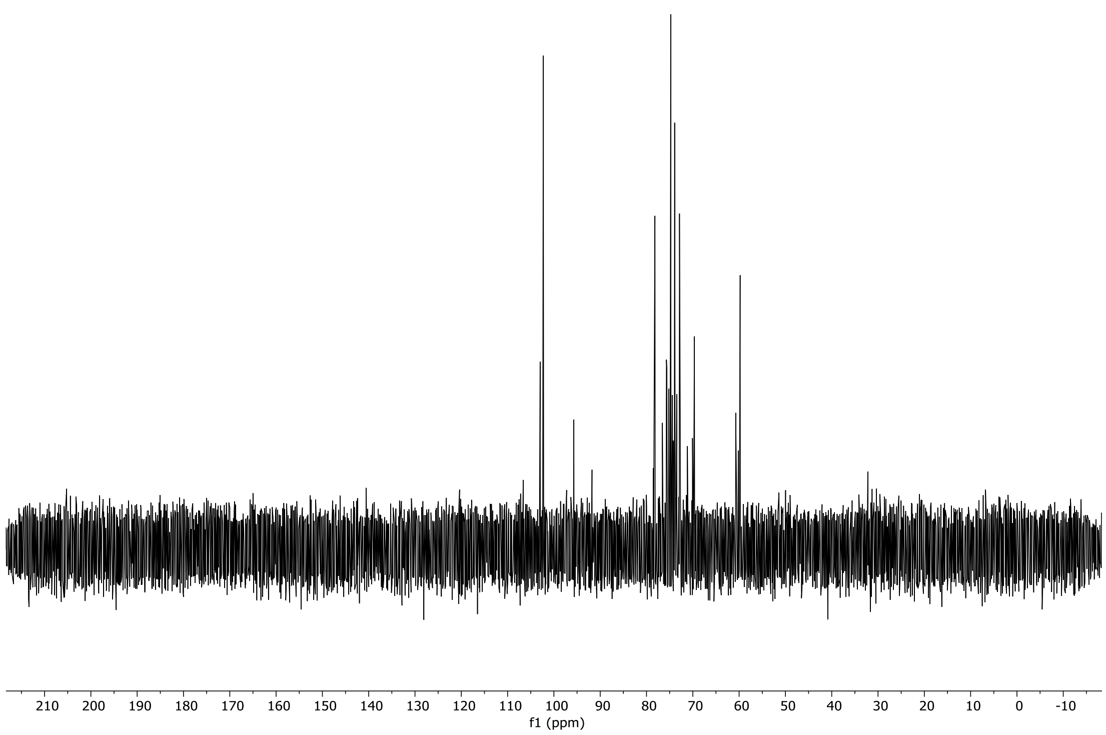

HSQC NMR of LD$_6$ (D$_2$O)

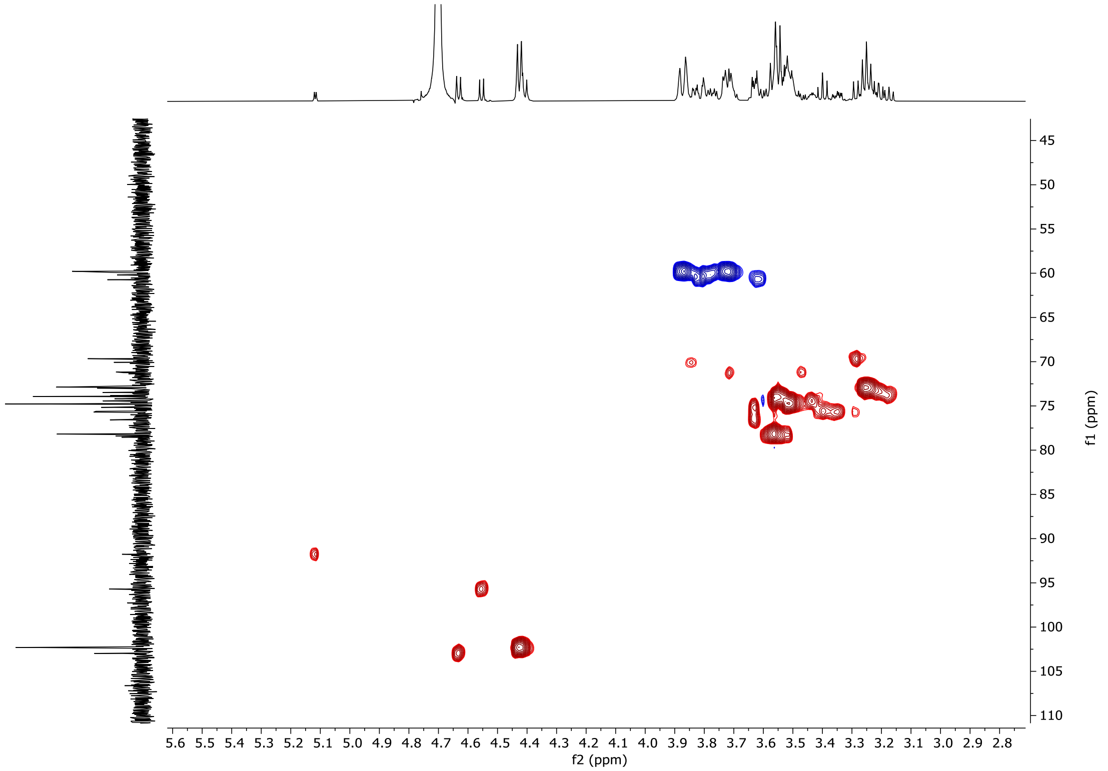

4 Oligosaccharides self-assembly

4.1 Solubility measurement

The lyophilized powder was weighed, MilliQ water was added in portions, and the mixture was bubbled with N_2 for 30 s. Upon visual disappearance of the precipitate a range of solubility was calculated. Water addition was stopped when the calculated solubility was < 0.5 mg/mL.

Compound	Mass (mg)	Volume upon dissolution (μL)	Solubility (mg/mL)
**D_5**	0.8	60	≥ 13.3
**D_6**	1.1	1000	≥ 1
**D_7**	0.9	-	< 0.5
**L_6**	1.1	1000	≥ 1
**L_3D_3**	1.0	20	≥ 50
**L_2D_4**	1.0	20	≥ 50
**LD_6**	1.0	200	≥ 5
**LD_6L**	1.1	2000	≥ 0.6
**LD_5L**	1.0	600	≥ 1.7

Table S1 Solubility measurement.

4.2 Recrystallization

The lyophilized compounds were dissolved in DMSO (10 mg/mL) under sonication in a heated bath at 40 °C until a clear solution was obtained. Crystallization was induced by the addition of MeOH (5 times the volume of DMSO used) to the DMSO solution. After 16 h the suspension was centrifuged, the supernatant removed and the solid washed three times with MeOH.

Compound	Dissolved in DMSO?	Precipitation rate
**D_6**	Yes	Fast
**D_6 + L_6 (1:1)**	Yes	Slow
**L_3D_3**	Yes	Slow
**L_2D_4**	Yes	Slow
**LD_6**	Yes	Fast
**LD_6L**	Yes	Fast
**LD_5L**	Yes	Intermediate

Table S2 Fast: within 5 min. Intermediate: within 2-3 h. Slow: more than 16 h

1.1. XRD analysis

X-ray diffraction experiments were carried out using a D8 Avance diffractometer (Bruker) in reflection mode with monochromatic Cu Kα radiation (λ= 1.5418 Å) generated at 40 kV and 40 mA (Siemens X-ray tube KFL CU 2K). The scans were performed in the scattering angle range between 4°and 40° with a step of 0.02°and an accumulation time of 6 or 10 s. Raw XRD profiles were corrected by subtraction of the sample holder signal, smoothing and baseline correction. The oligosaccharide samples were lyophilized prior to XRD measurement.

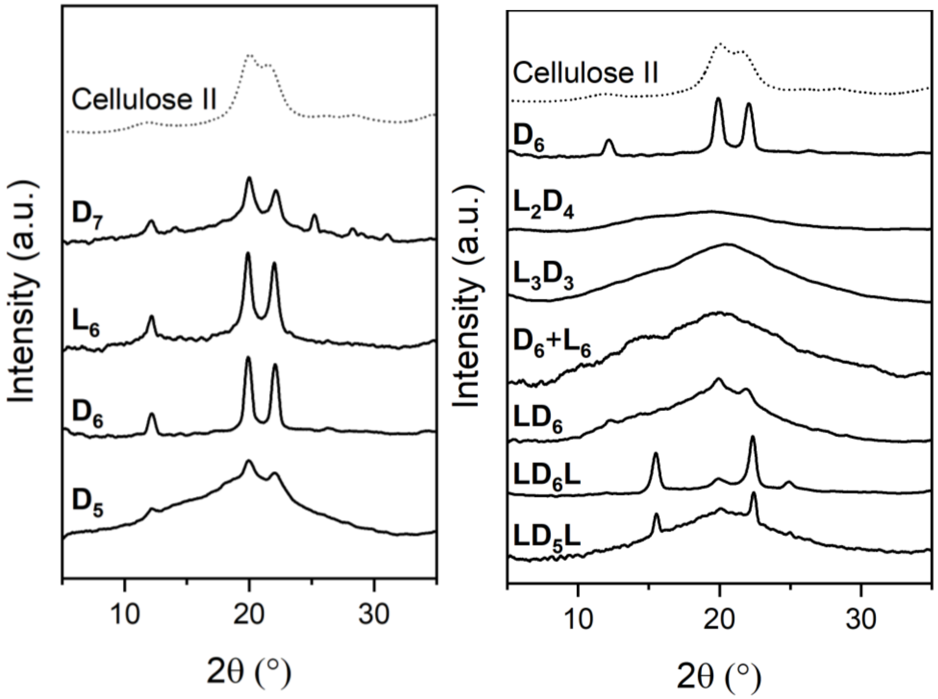

Figure S3 XRD profiles obtained from lyophilized powder after synthesis.

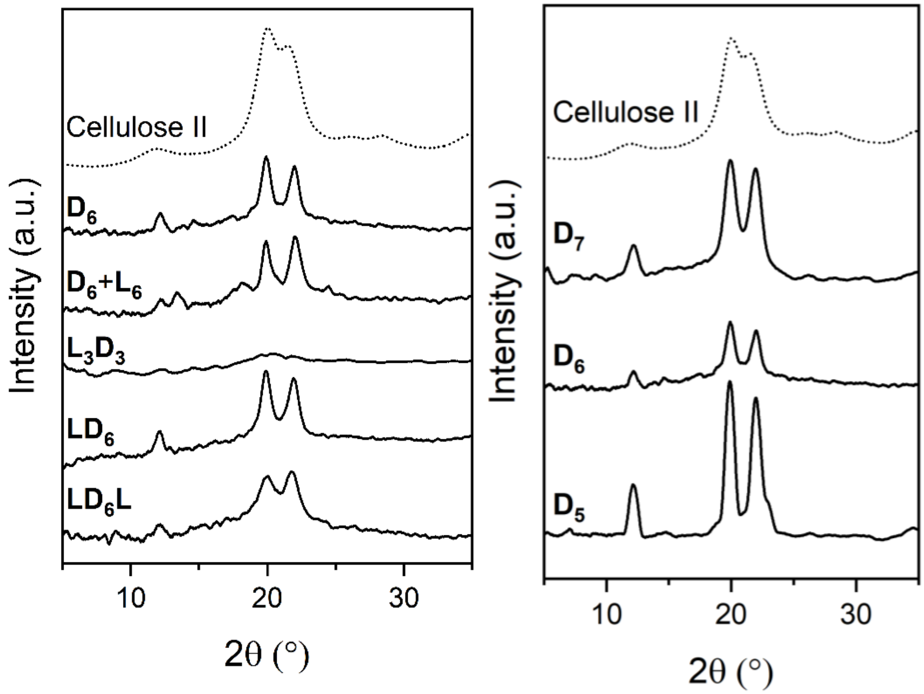

Figure S4 XRD profiles obtained from lyophilized powders after recrystallization.

4.3 TEM imaging

Transmission electron microscopy was performed using a JEM 2100Plus transmission electron microscope (Jeol, Japan) operated at an accelerating voltage of 200 kV or a FEI Talos L120C (Thermo Fisher, USA) operated at an accelerating voltage of 120 kV (LaB_6 cathode). Drops (3-4 μL) of aqueous suspensions (or MeOH suspensions) of crystallites were deposited on glow-discharged carbon-coated copper grids. Negative staining (2% uranyl acetate aqueous solution) was applied (3 μL drop) to the grid after the sample was deposited, allowed to settle for approximately 1 min, and then carefully blotted away with filter paper. Negative staining of the TEM grids has been used to enhance contrast only where specified in the images reported below.[10]

4.3.1 $\mathbf{D_6}$

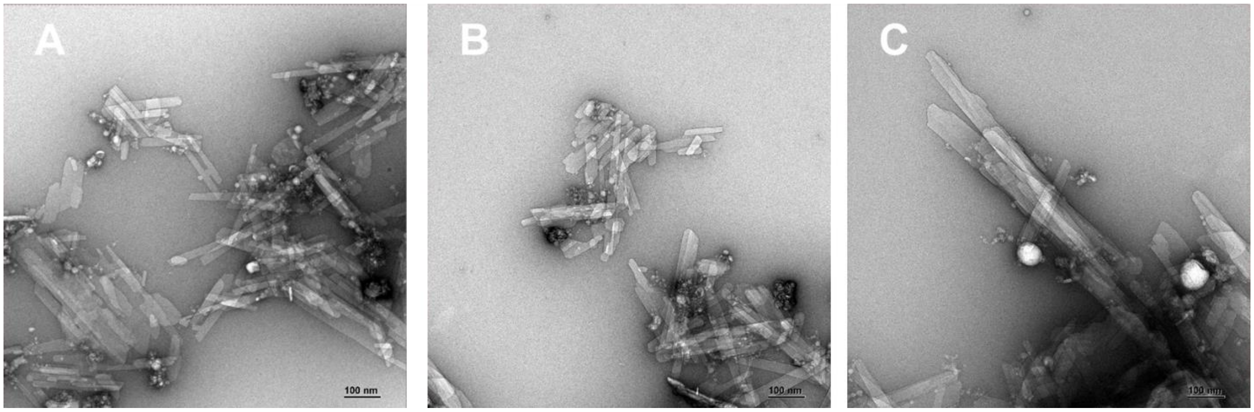

Figure S5 Representative TEM images of $\mathbf{D_6}$ obtained from aqueous suspension (1 mg/mL) using negative staining.

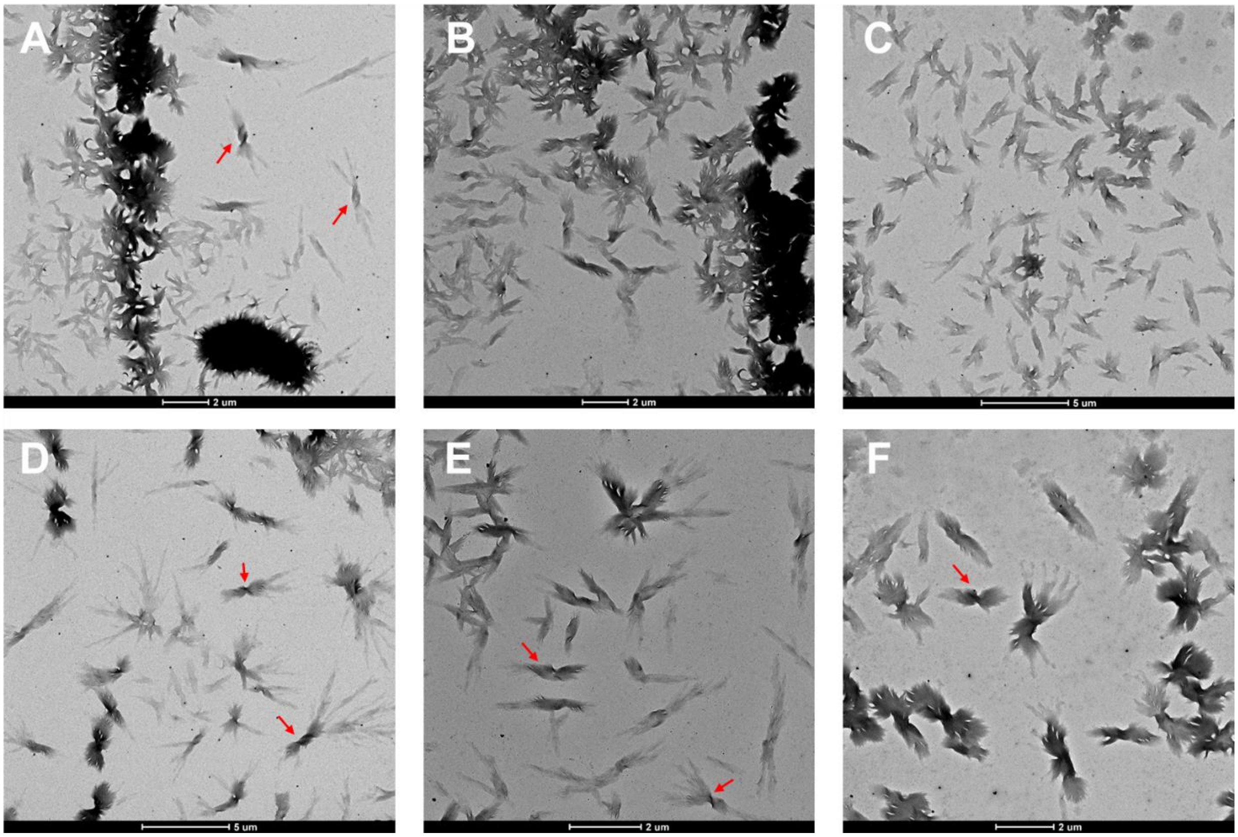

Figure S6 Representative TEM images of $\mathbf{D_6}$ obtained from aqueous suspension (1 mg/mL). The red arrows indicate the assemblies where the features indicating a twisted morphology are more evident.

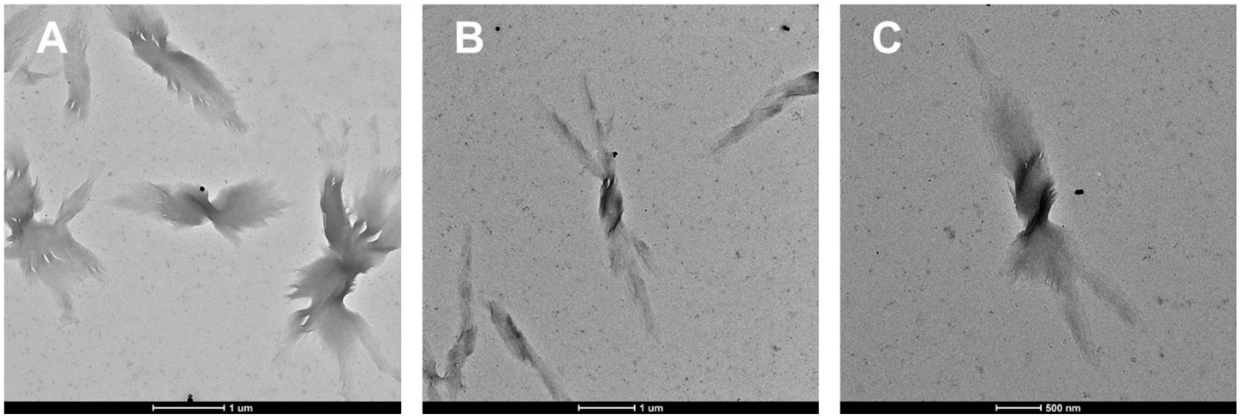

Figure S7 Representative TEM images of $\mathbf{D_6}$ twisted assemblies at high magnifications obtained from aqueous suspension (1 mg/mL).

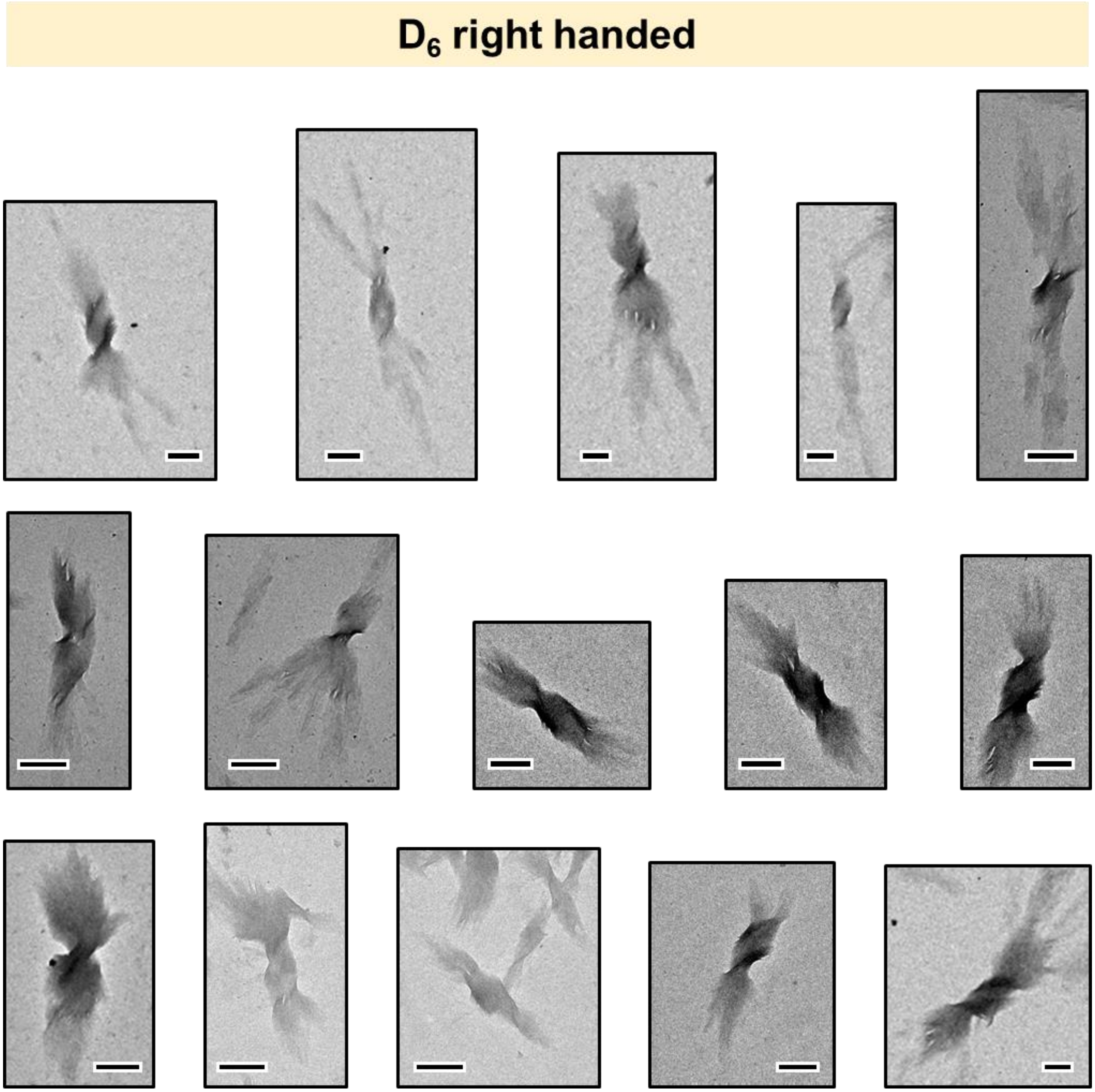

Figure S8 Excerpts of TEM images of **D$_6$** twisted assemblies obtained from aqueous suspension. Scale bar 500 nm.

4.3.2 L_6

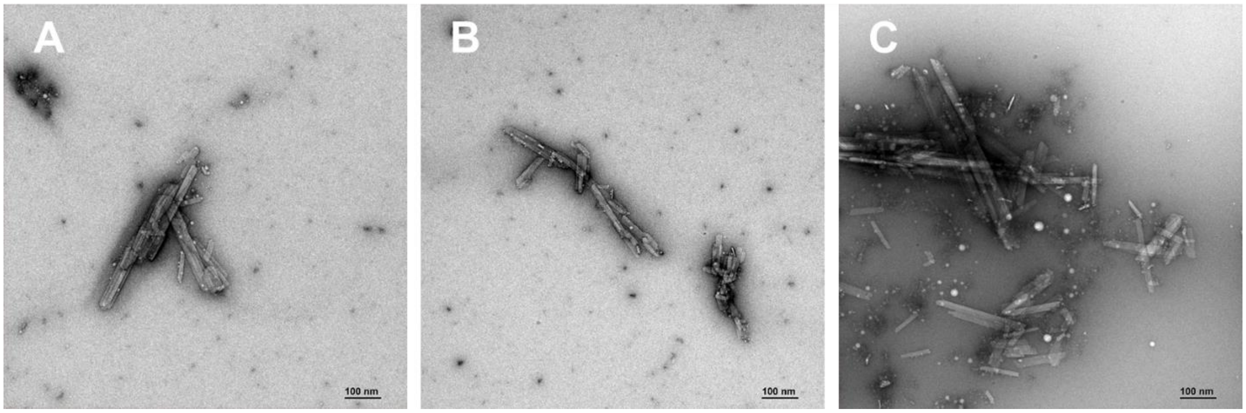

Figure S9 Representative TEM images of $\mathbf{L_6}$ obtained from aqueous suspension (1 mg/mL) using negative staining.

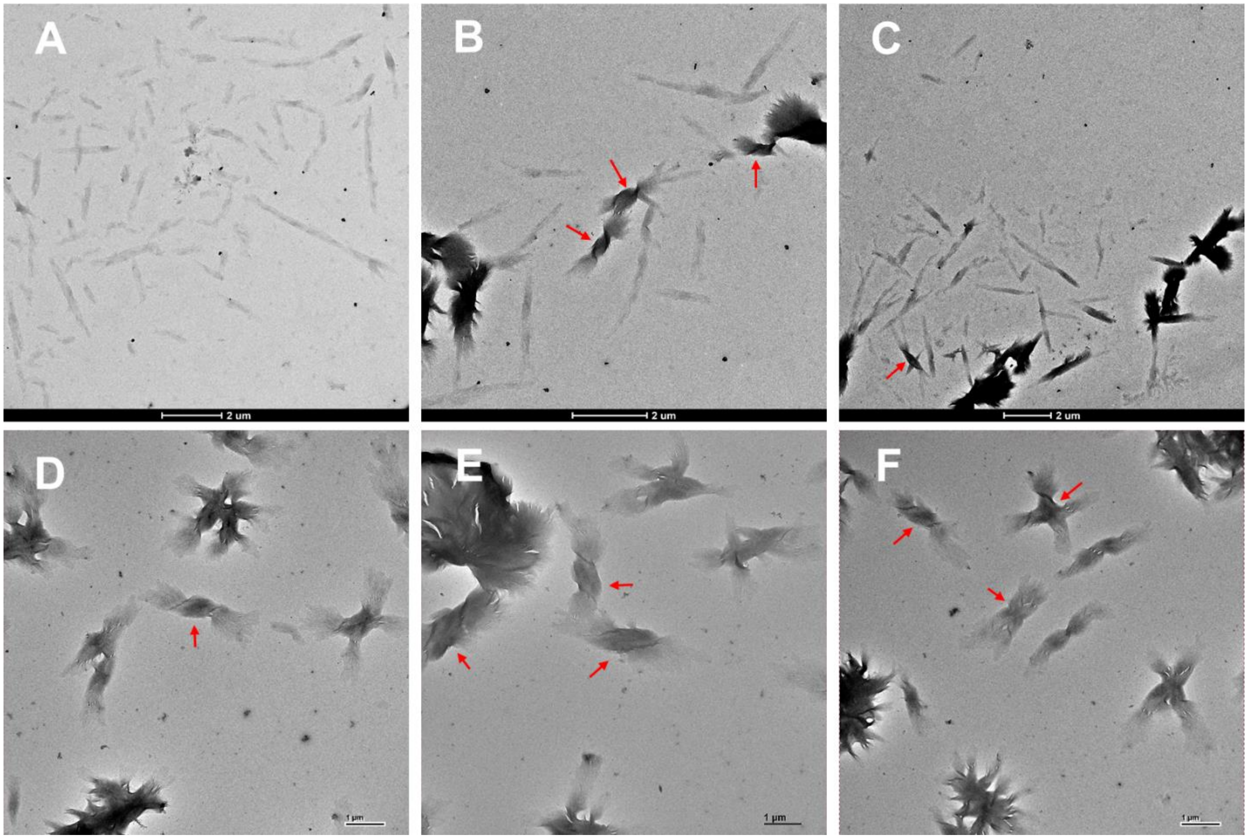

Figure S10 Representative TEM images of $\mathbf{L_6}$ obtained from aqueous suspension (1 mg/mL). The red arrows indicate the assemblies where the features indicating a twisted morphology are more evident.

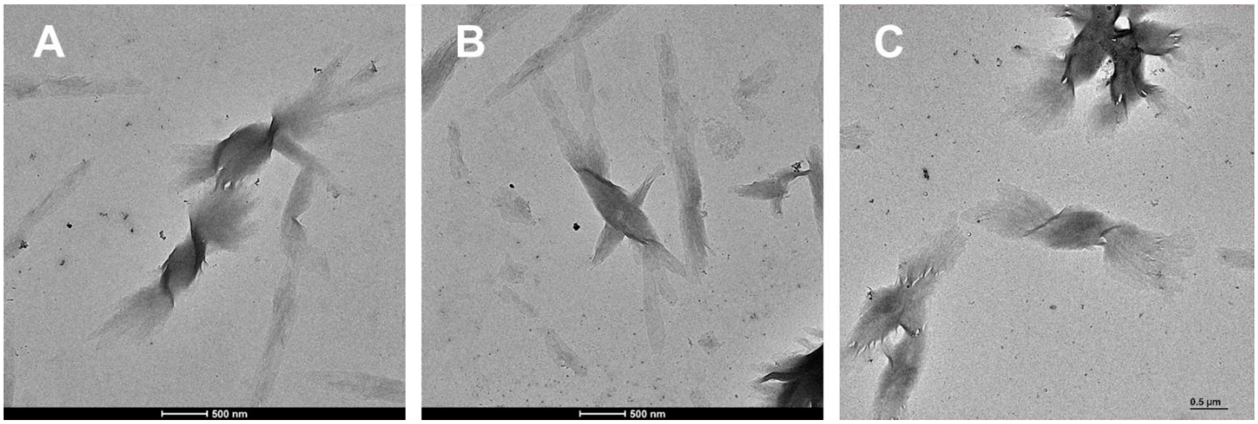

Figure S11 Representative TEM images of $\mathbf{L_6}$ twisted assemblies at high magnifications obtained from aqueous suspension (1 mg/mL).

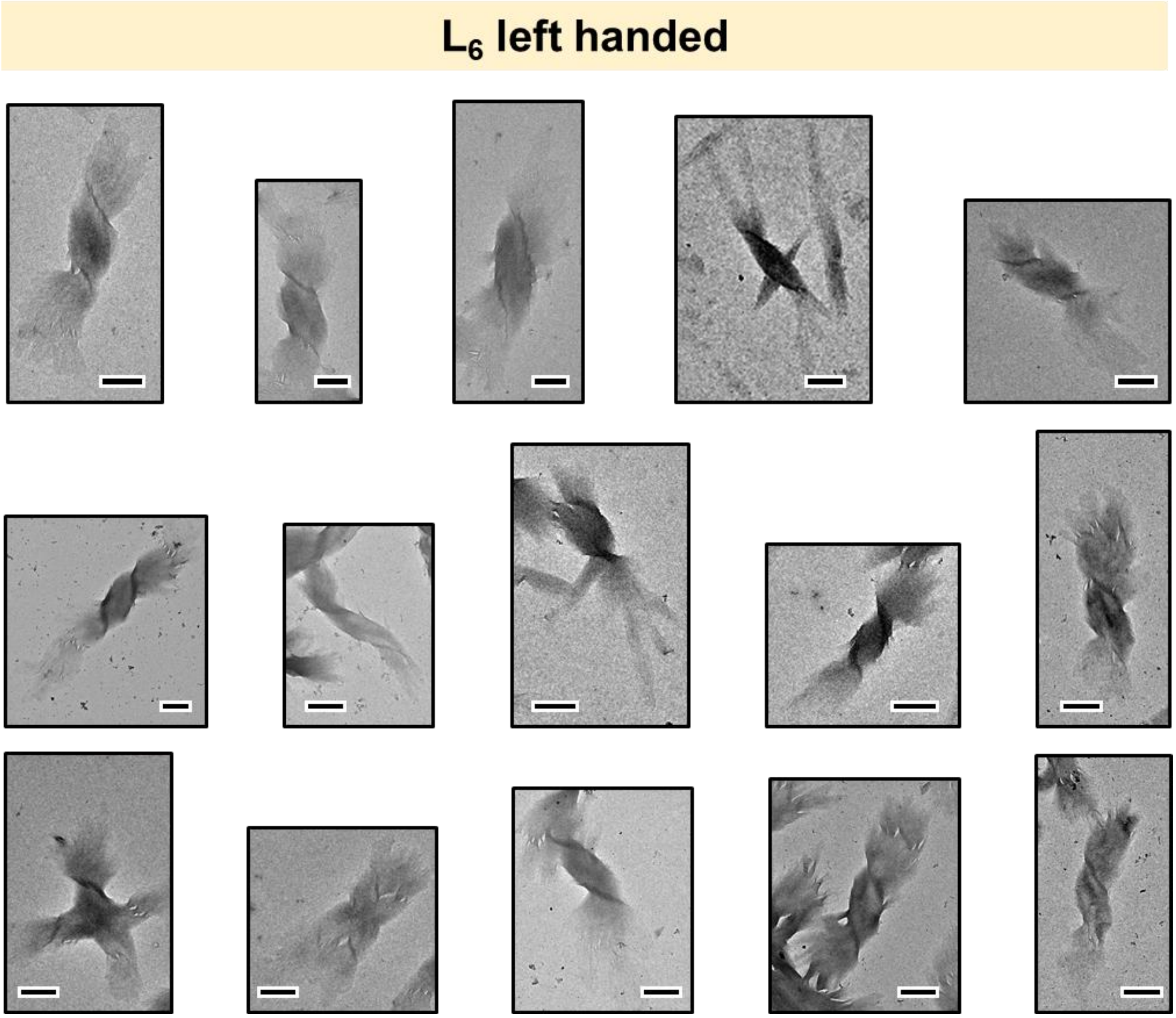

Figure S12 Excerpts of TEM images of **L_6** twisted assemblies obtained from aqueous suspension. Scale bar 500 nm.

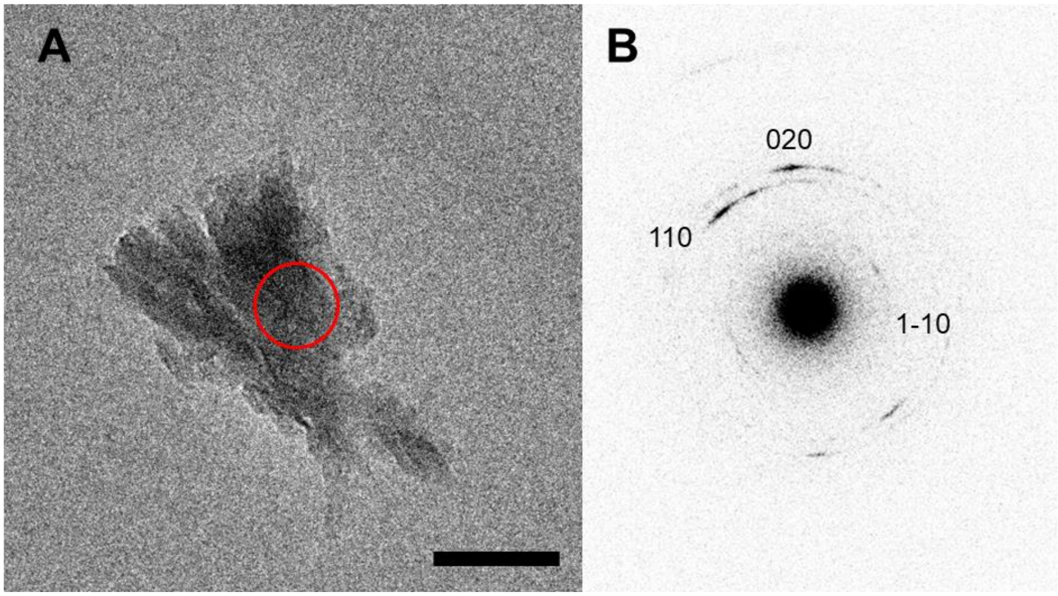

Figure S13 A-B) Tem image and electron diffraction analysis of $\mathbf{L_6}$ bundles obtained from aqueous suspension (1 mg/mL). The pattern was assigned to the cellulose II allomorph. Scale bar 500 nm.

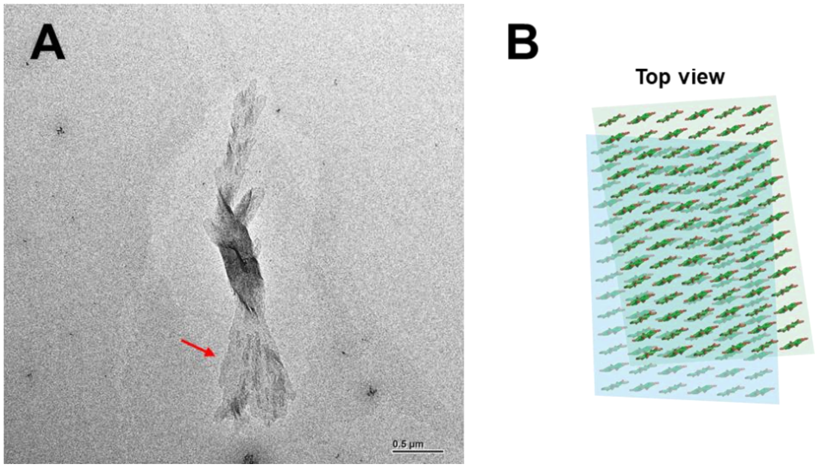

Figure S14 A) Representative TEM images of $\mathbf{L_6}$ obtained from aqueous suspension (1 mg/mL) showing a fan-like arrangement of the stacking platelets (*red arrow*). B) The fan-like arrangement of the stacking platelets was interpreted as a rotation between the (001) planes.

4.3.3 D_7

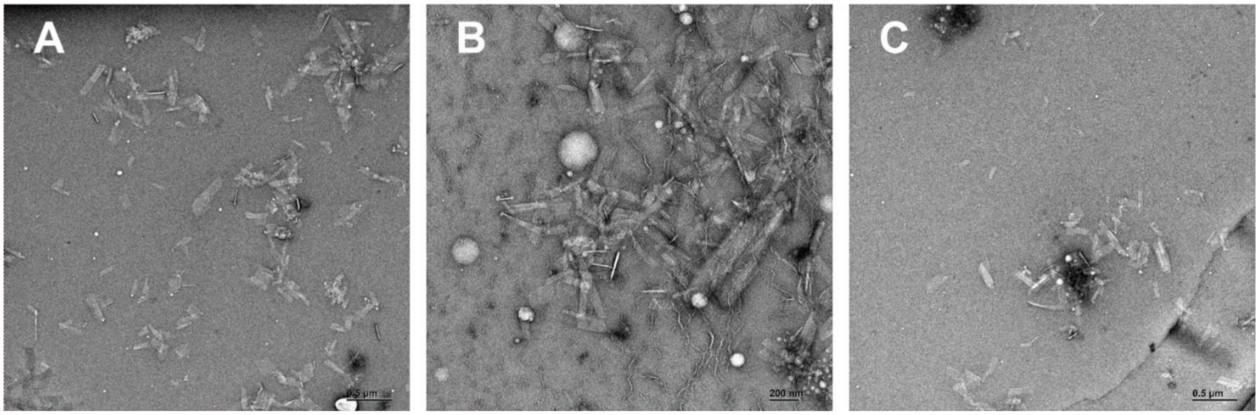

Figure S15 Representative TEM images of $\mathbf{D_7}$ obtained from aqueous suspension (1 mg/mL) using negative staining.

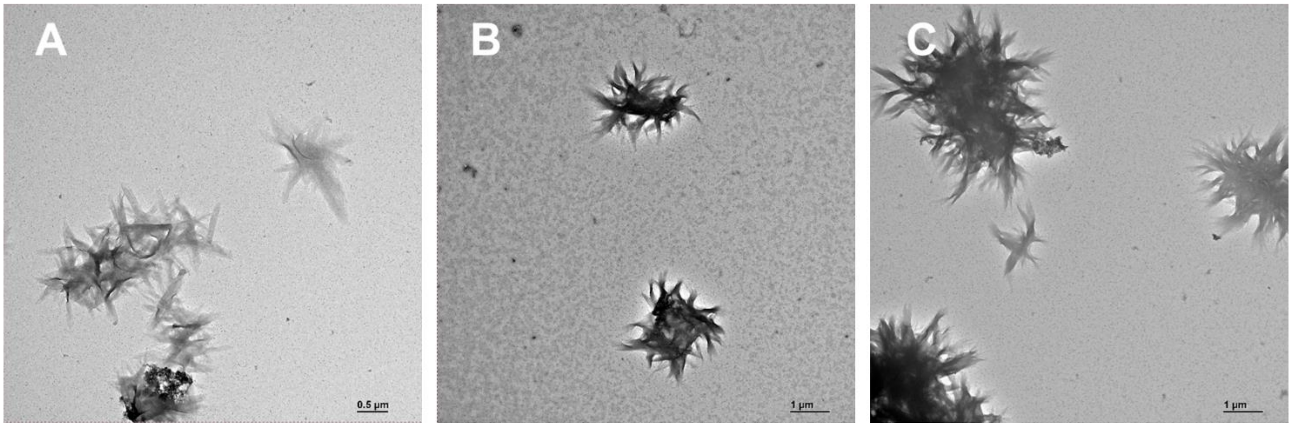

Figure S16 Representative TEM images of $\mathbf{D_7}$ obtained from aqueous suspension (1 mg/mL).

4.3.4 D_8

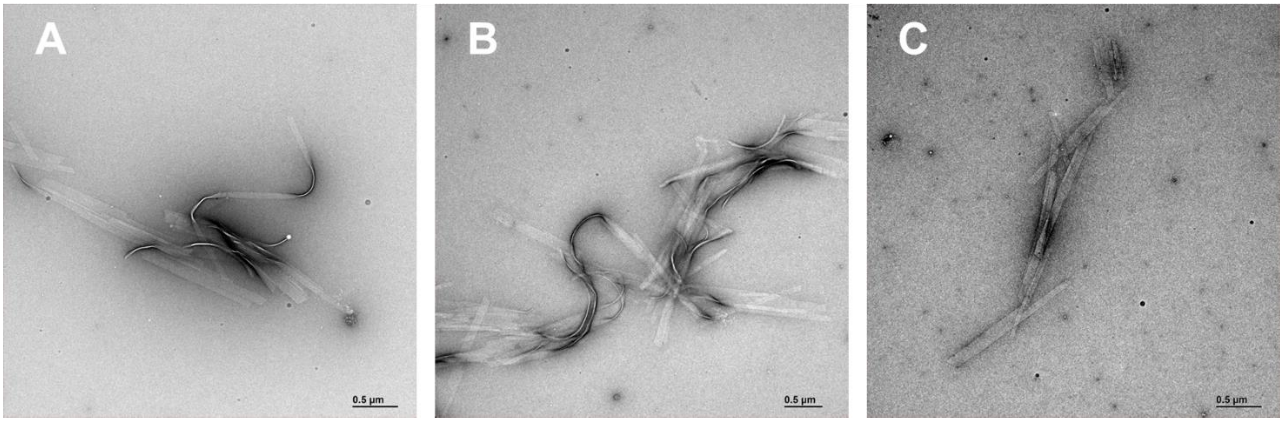

Figure S17 Representative TEM images of $\mathbf{D_8}$ obtained from aqueous suspension (1 mg/mL) using negative staining.

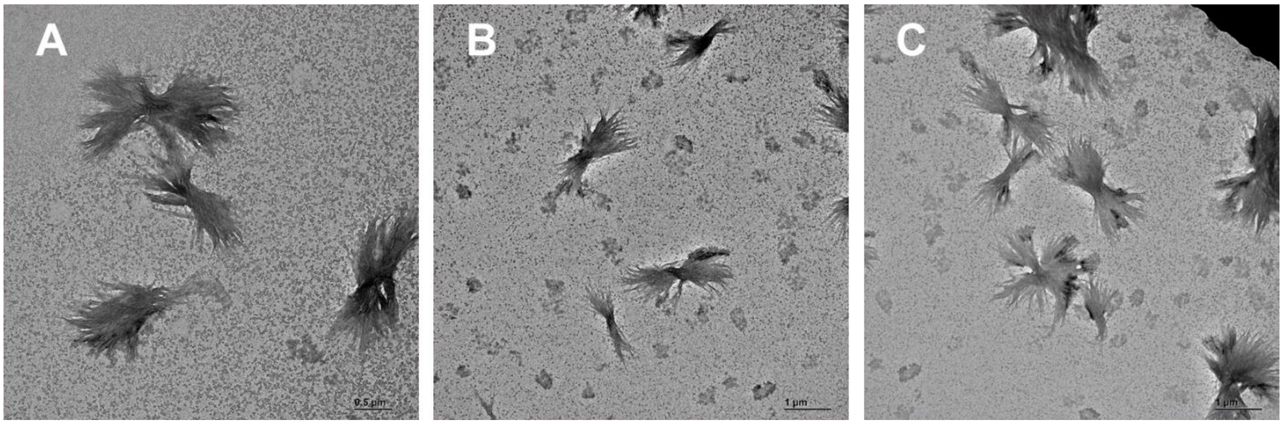

Figure S18 Representative TEM images of $\mathbf{D_8}$ obtained from aqueous suspension (1 mg/mL).

4.3.5 Mechanism of formation of twisted bundles

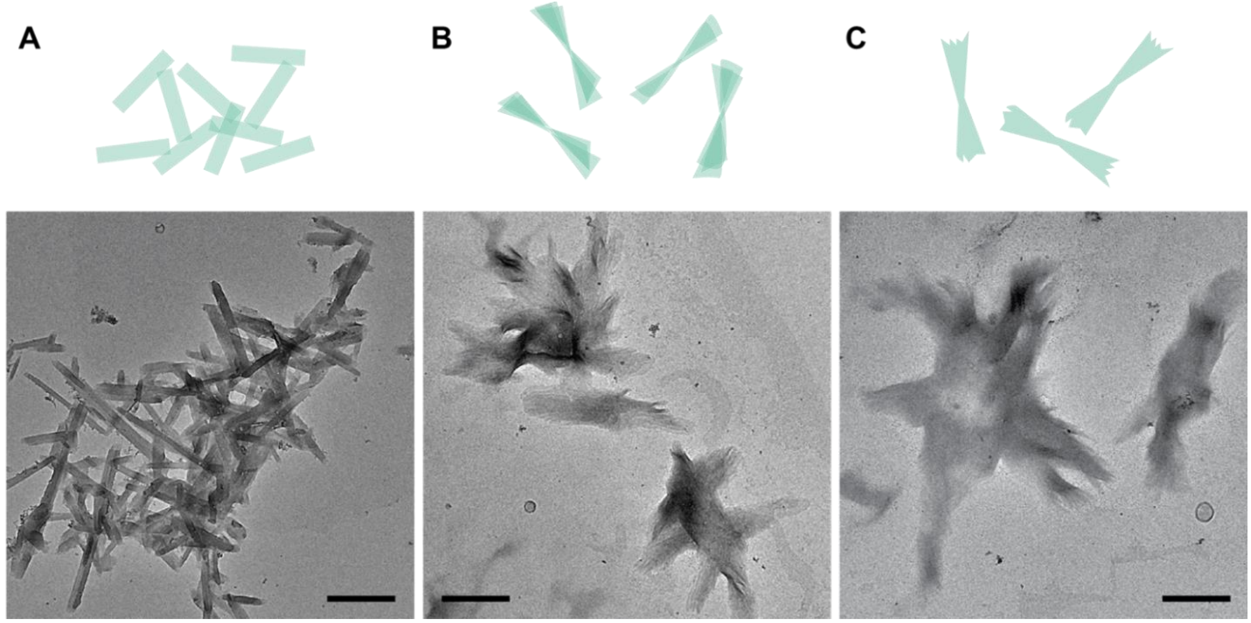

Figure S19 TEM images of grids obtained with different blotting strength of **L₆**. A) Extensive blotting yielded mainly discrete platelets. B) Low blotting strength induces formation of large bundles with platelets stacking. C) At the lowest blotting strength the bundles composed of platelets show no edge features suggesting that the platelets tend to merge (or fuse). Scale bars 200 m.

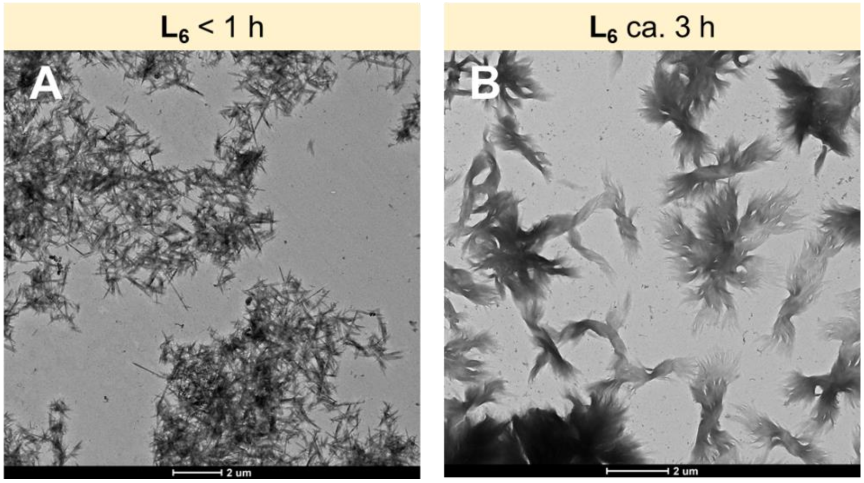

Figure S20 Representative TEM images of **L₆** obtained from aqueous suspension (1 mg/mL or 0.5 mg/mL) varying the evaporation rate. A) The grid was allowed to dry (< 1 h). B) The solvent evaporation rate was slowed down by covering the grid with a petri dish (ca. 3 h).

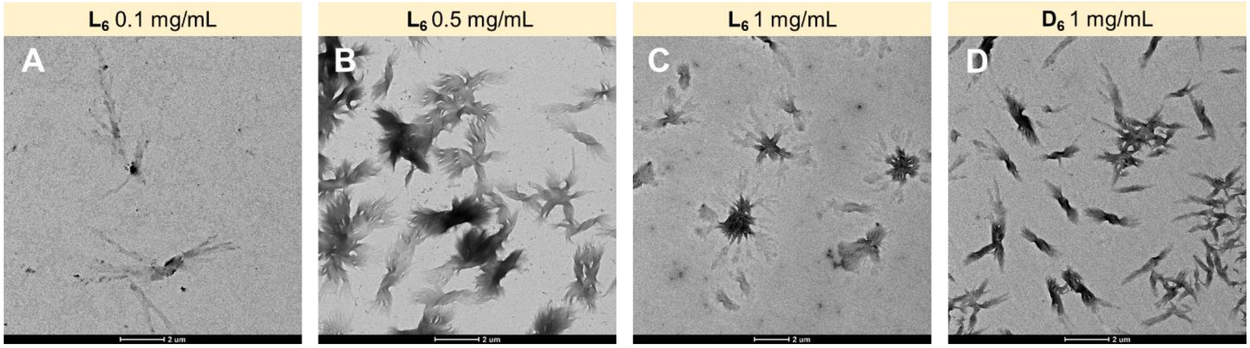

Figure S21 Representative TEM images of $\mathbf{L_6}$ (A-C) and $\mathbf{D_6}$ (D) obtained from aqueous suspension at different concentration. The solvent evaporation rate was slowed down by covering the grid with a petri dish (ca. 3 h).

4.3.6 D_6+L_6

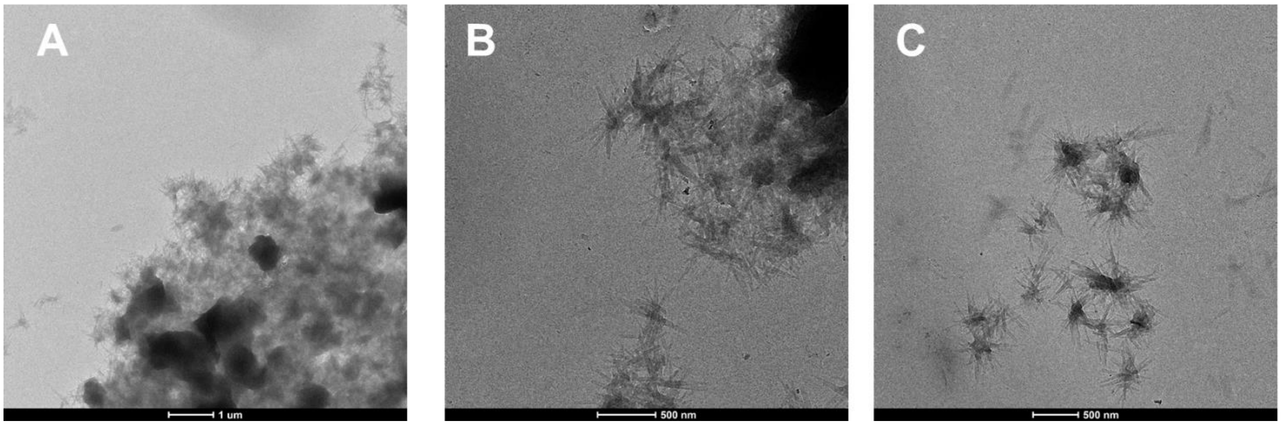

Figure S22 Representative TEM images of $\mathbf{D_6}$+$\mathbf{L_6}$ (1:1 ratio) obtained from MeOH suspension after recrystallization.

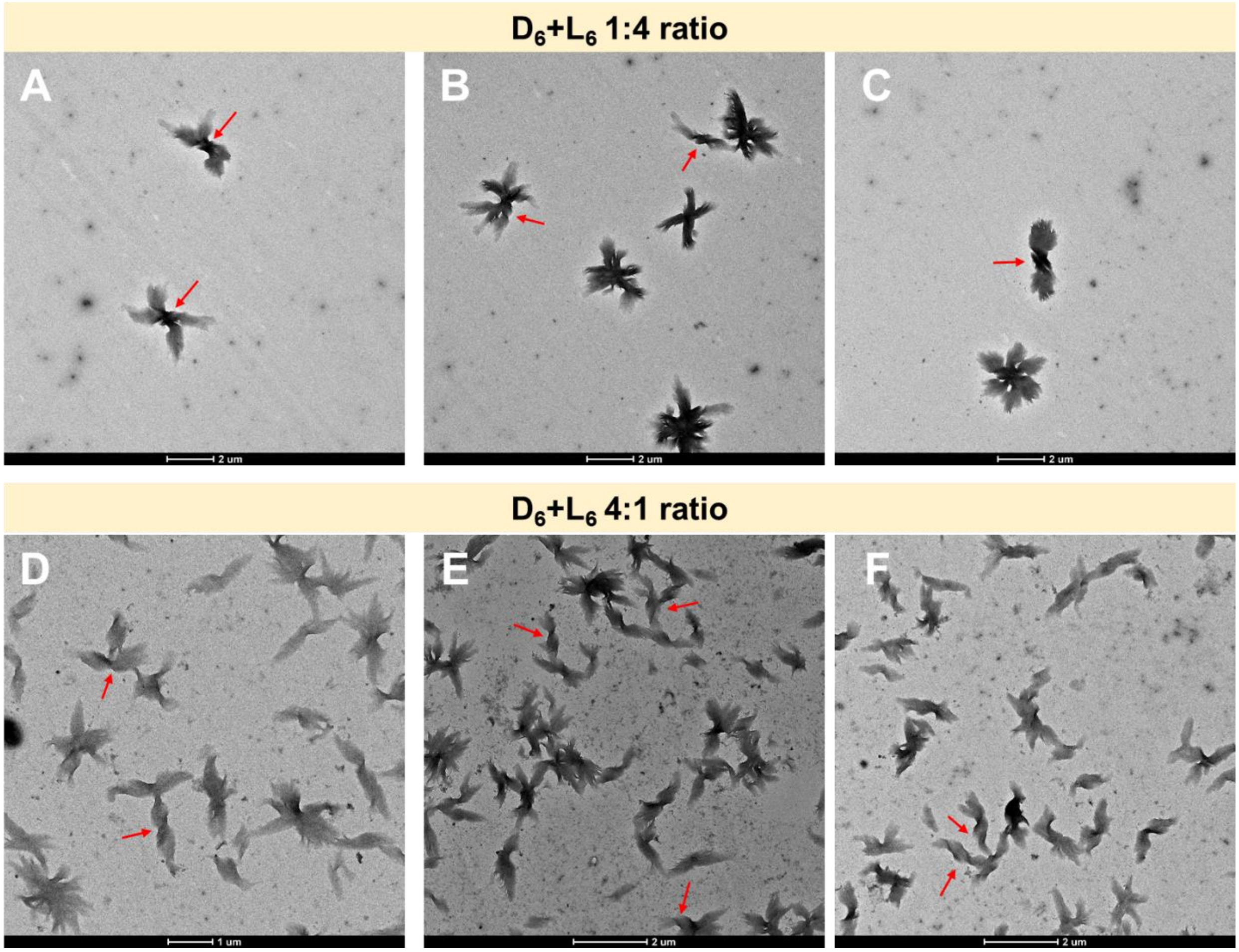

Figure S23 A-C) Representative TEM images of **D_6+L_6** (1:4 ratio) obtained from aqueous suspension (1 mg/mL). The red arrows indicate left handed chirality. D-E) Representative TEM images of **D_6+L_6** (4:1 ratio) obtained from aqueous suspension (1 mg/mL). The red arrows indicate right handed chirality.

4.3.7 LD$_6$

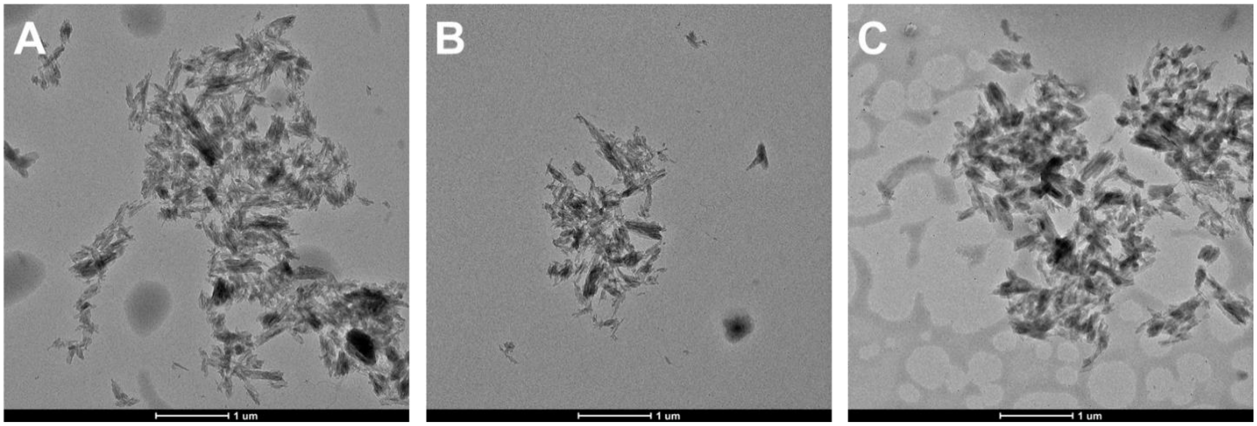

Figure S24 Representative TEM images of **LD$_6$** platelets obtained from MeOH suspension after recrystallization.

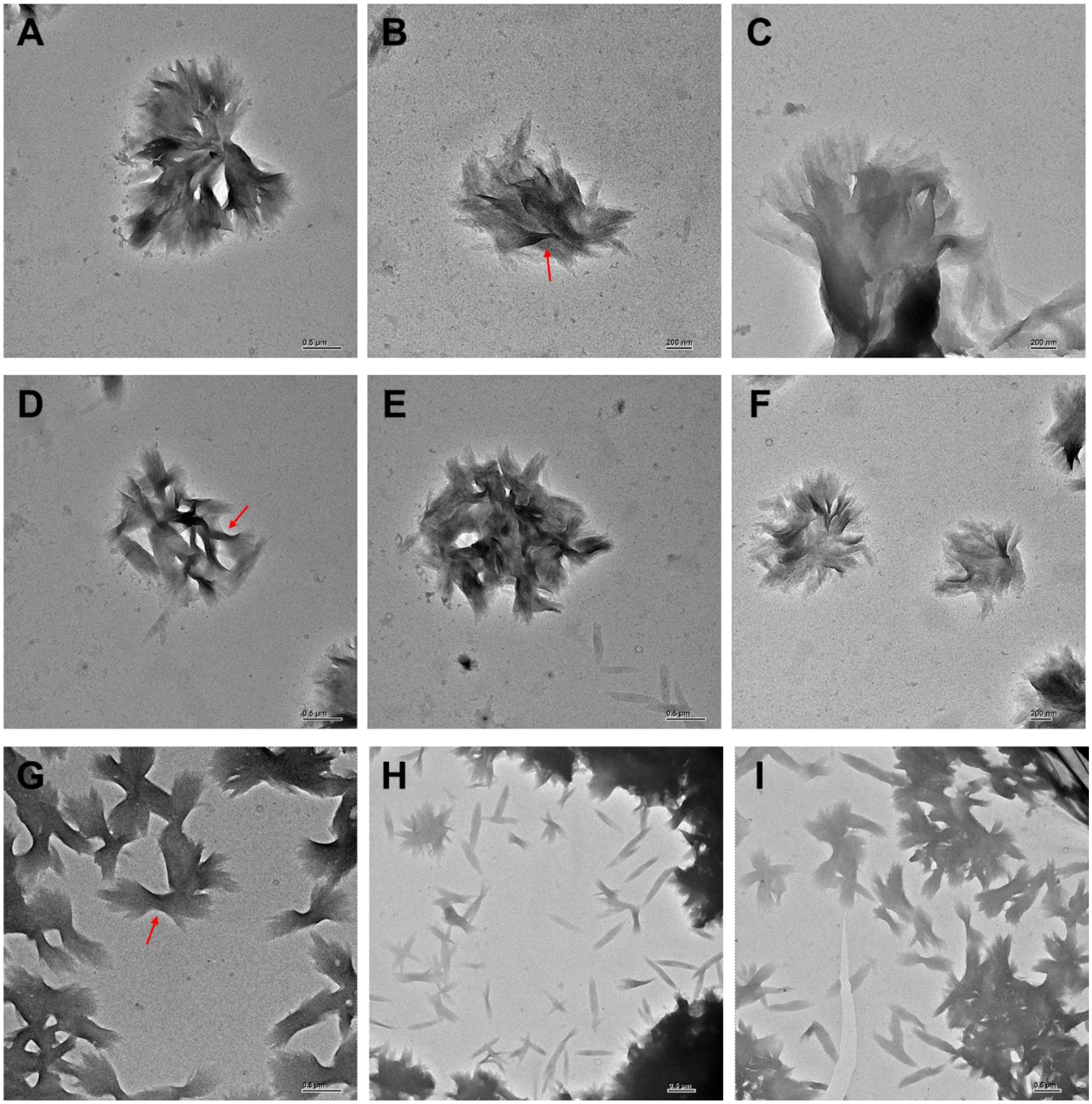

Figure S25 Representative TEM images of **LD_6** bundles obtained from aqueous suspension (1 mg/mL).

4.3.8 LD_6L

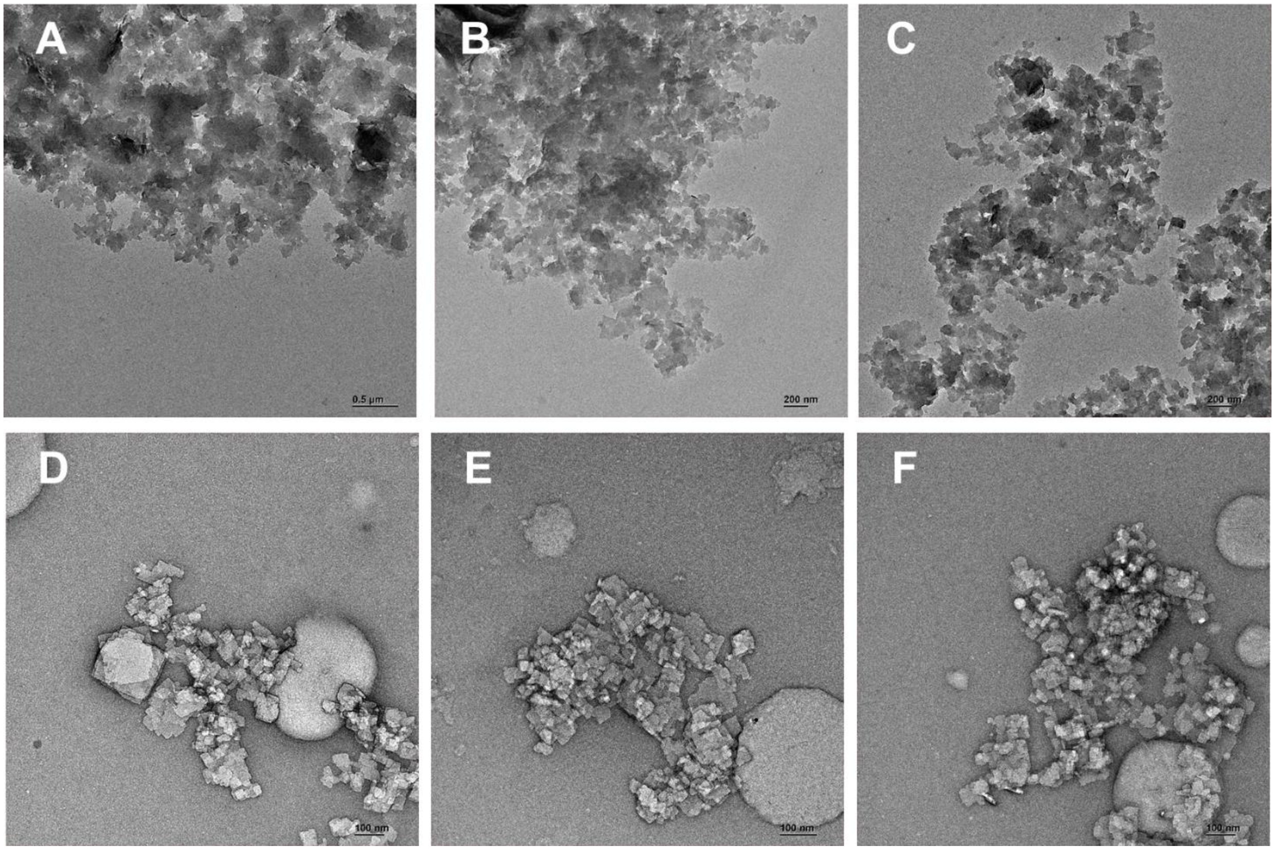

Figure S26 A-C) Representative TEM images of **LD_6L** obtained from aqueous suspension (1 mg/mL). D-F) Representative TEM images of **LD_6L** obtained from aqueous suspension (1 mg/mL) using negative staining.

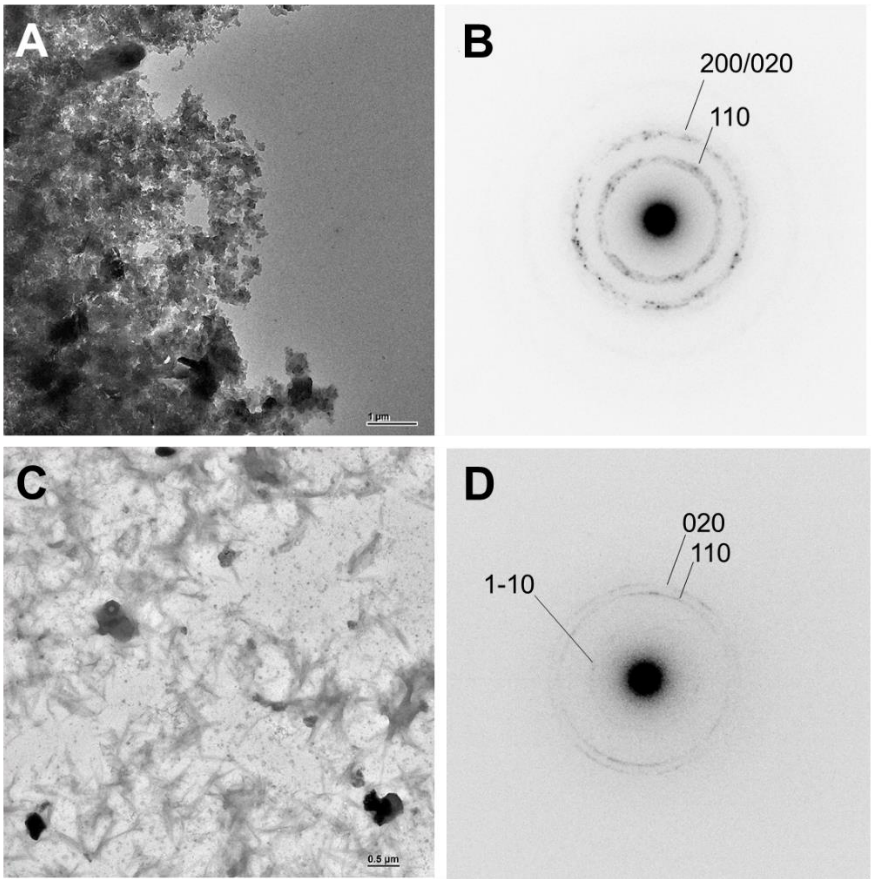

Figure S27 A, B) Electron diffraction analysis of **LD$_6$L** obtained from aqueous suspension (1 mg/mL). The pattern was assigned to the cellulose IV$_{II}$ allomorph. C, D) Electron diffraction analysis of **LD$_6$L** obtained from aqueous suspension after recrystallization. The pattern was assigned to the cellulose II allomorph.

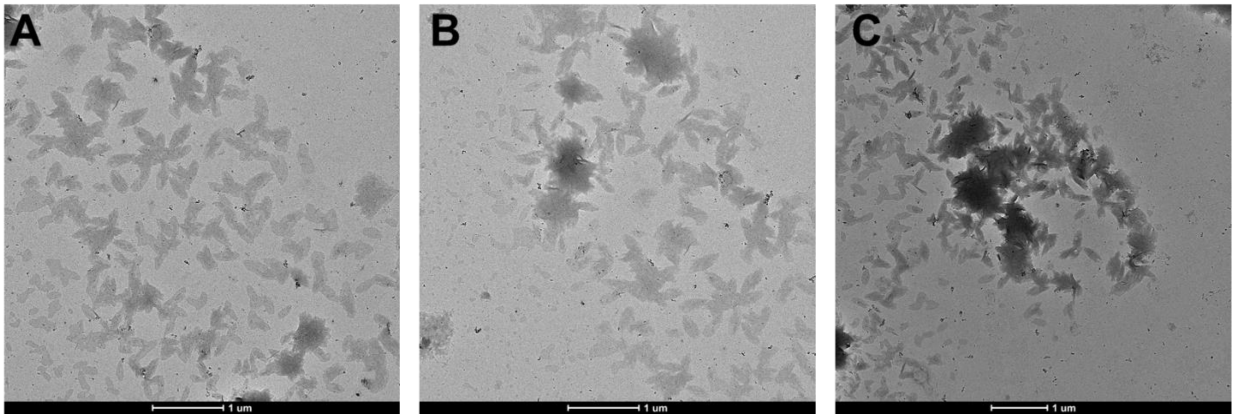

Figure S28 Representative TEM images of **LD$_6$L** platelets obtained from MeOH suspension after recrystallization.

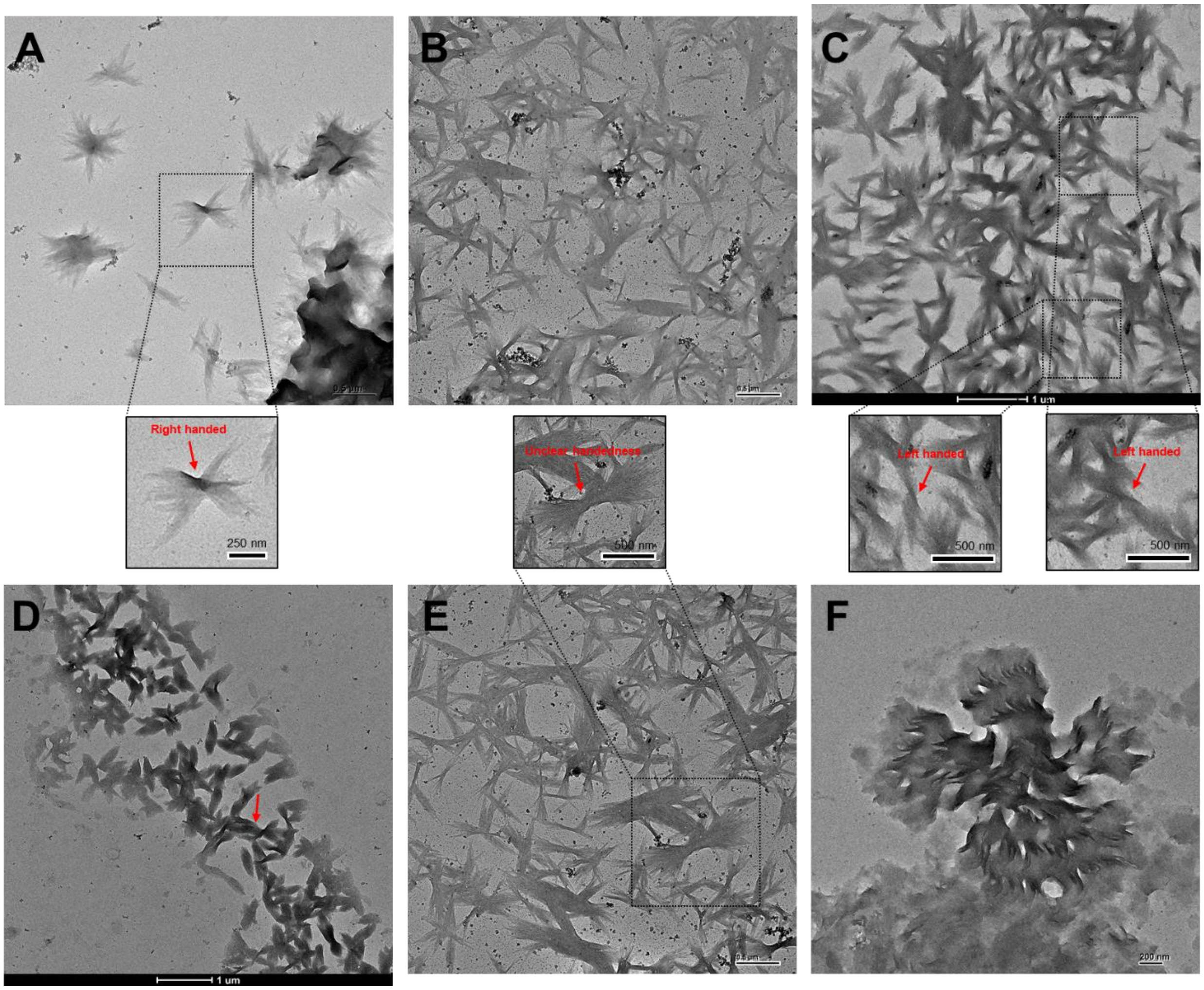

Figure S29 Representative TEM images of **LD$_6$L** bundles obtained from MeOH suspension after recrystallization.

4.3.9 LD_5L

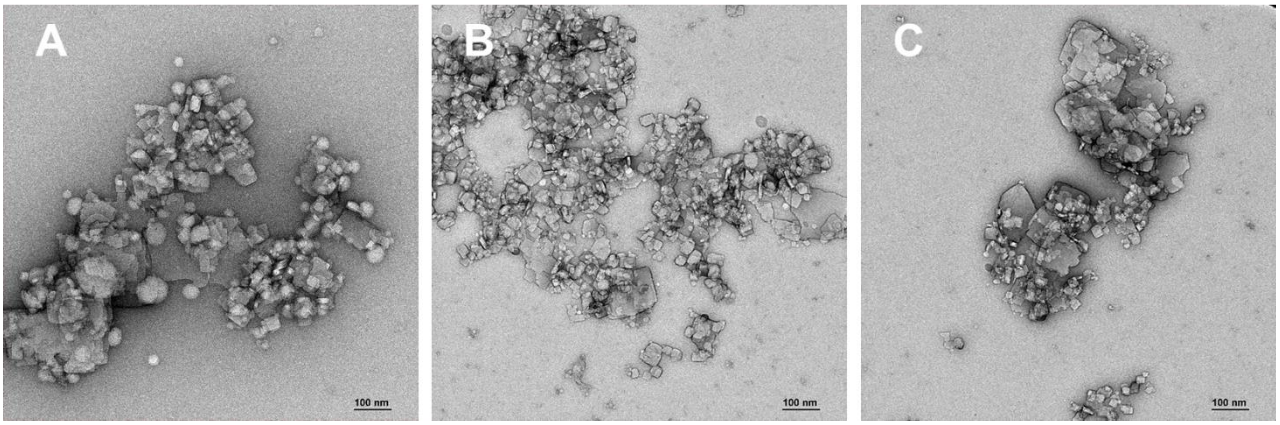

Figure S30 Representative TEM images of **LD_5L** obtained from aqueous suspension (1 mg/mL) using negative staining.

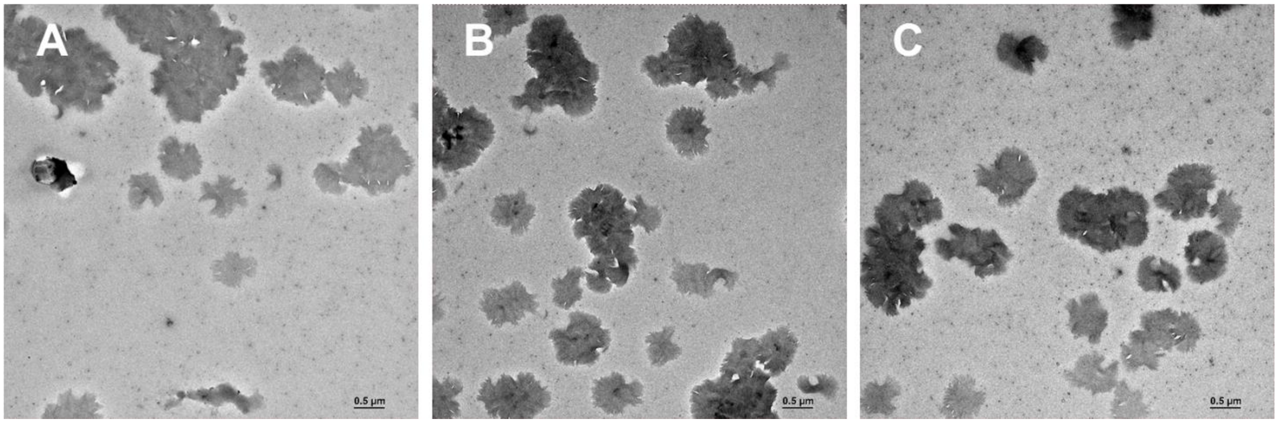

Figure S31 Representative TEM images of **LD_5L** obtained from MeOH suspension after recrystallization.

4.4 AFM and SEM imaging

Atomic force microscopy was performed with a JPK NanoWizard 4 AFM in tapping mode (AC mode) or a Dimension ICON instrument (Bruker) in pulse force (PeakForce) mode using SNL-10 A tip (0.35 N/m, 65 kHz, Bruker) or Arrow NCR tip (42 N/m, 285 kHz, Nano World). The samples were prepared as follows: approximately 0.1 mg of the lyophilized powder was weighed (in a glass or plastic vial), diluted with MilliQ water to reach the concentration of 1 mg/mL. For AFM imaging, the solution was further diluted with MilliQ water to reach the concentration of 0.1 mg/mL. Drops of aqueous suspensions were deposited on freshly cleaved mica or on glow-discharged (0.8 mbar, 30 mA for 20 s using air) silicon wafer and dried at room temperature. AFM images were collected with 1024 x 1024 pixels/frame and analyzed with the JPK Data Processing software.

Scanning electron microscopy (SEM) images were obtained with a Gemini SEM, LEO 1550 system with cold field emission gun operation at 3 kV. All the samples were coated with Au/Pd. The hydrophilic glass substrate was prepared by treating a round shape glass slide with an HCl aqueous solution (0.5 M) for 1 h and then rinsed with water.

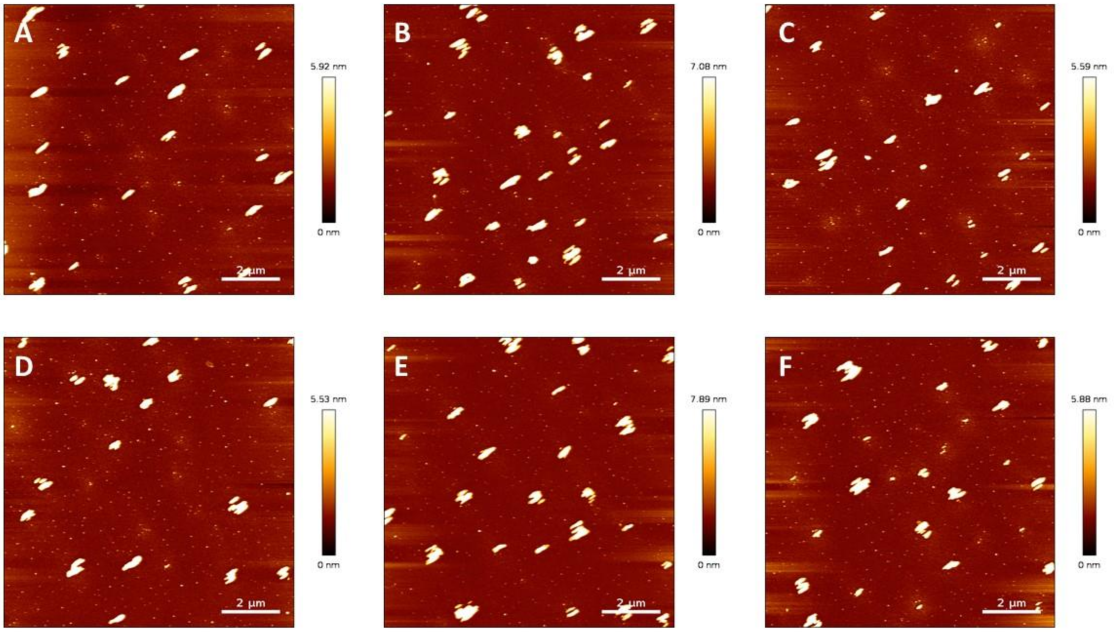

Figure S32 Representative AFM images of $\mathbf{D_6}$ obtained from aqueous suspension (0.1 mg/mL) drop casted on freshly cleaved mica.

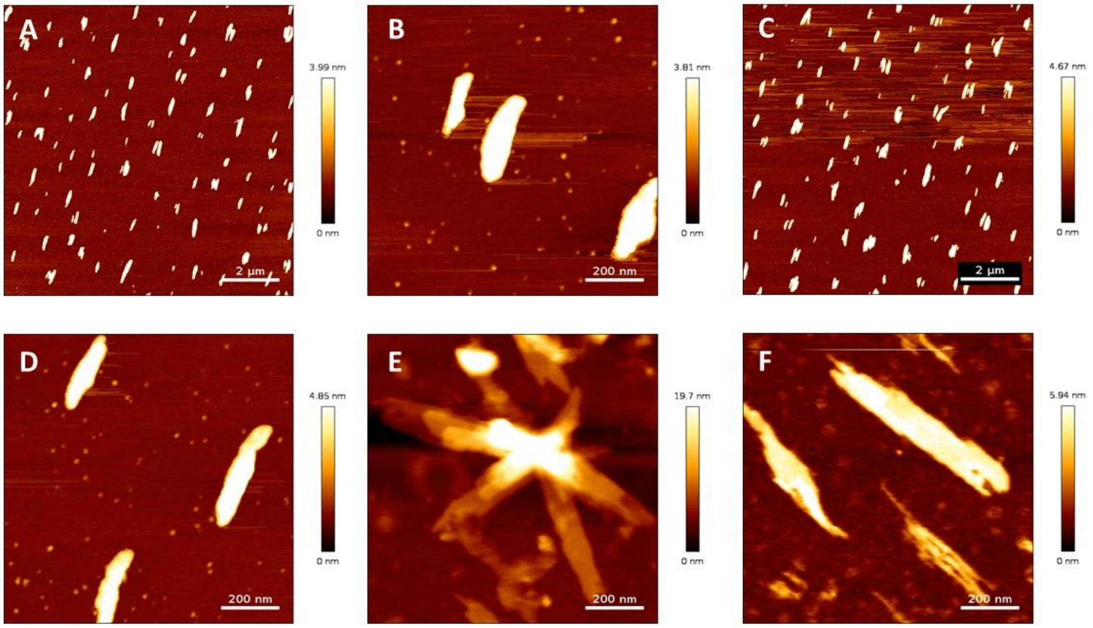

Figure S33 Representative AFM images of $\mathbf{D_7}$ obtained from aqueous suspension (0.1 mg/mL) drop casted on freshly cleaved mica.

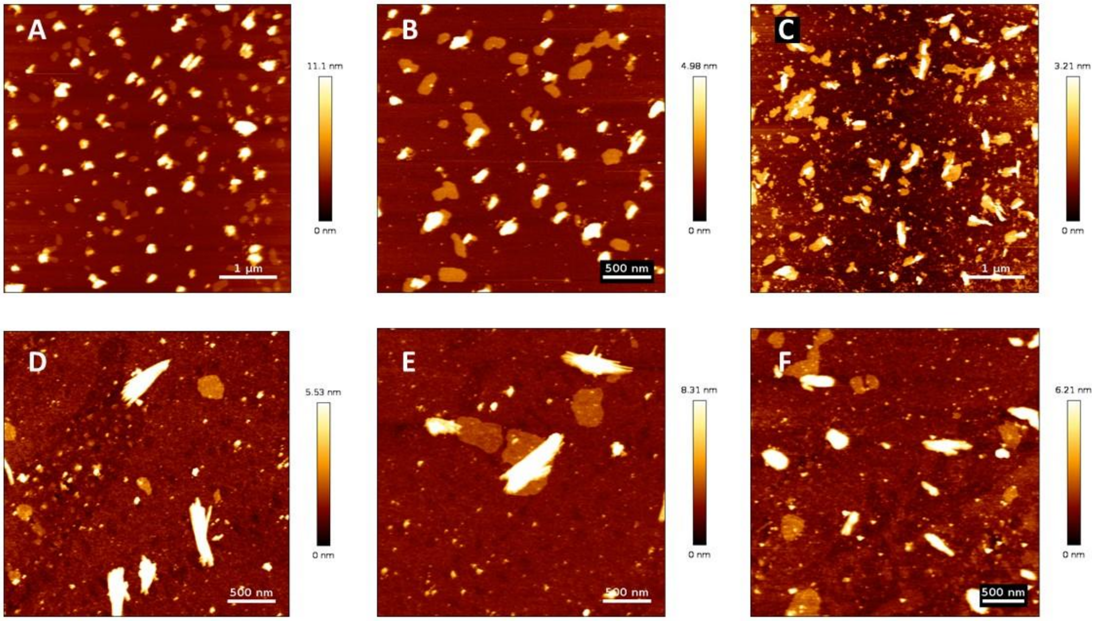

Figure S34 Representative AFM images of $\mathbf{D_8}$ obtained from aqueous suspension (approx. 0.1 mg/mL) drop casted on freshly cleaved mica (A-C) and silicon wafer (D-F).

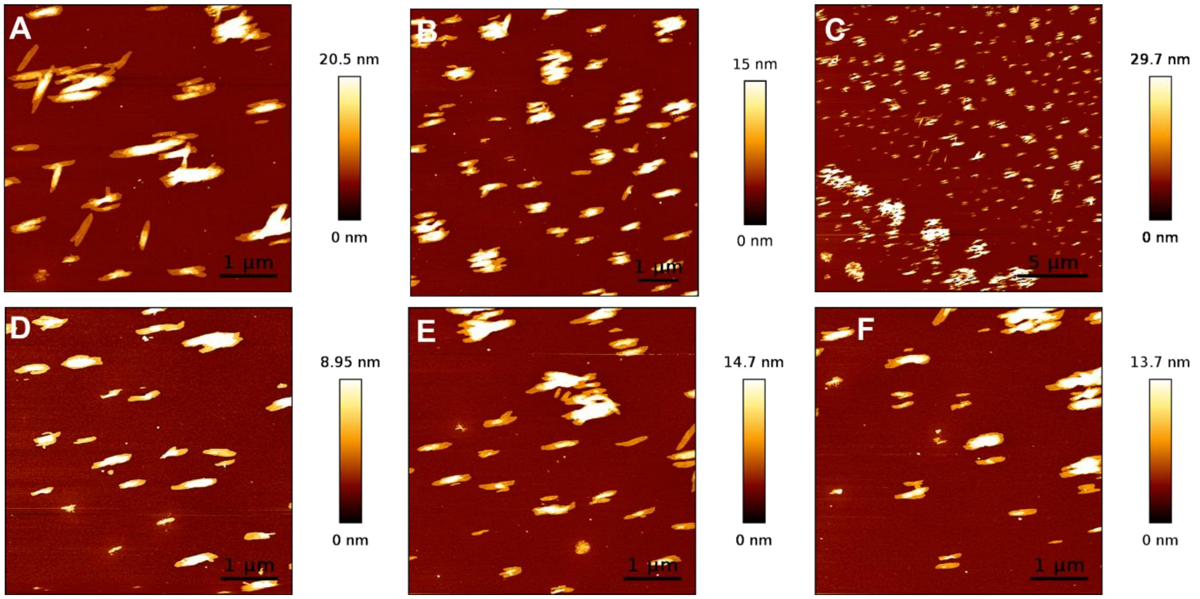

Figure S35 Representative AFM images of **L_6** obtained from aqueous suspension (0.1 mg/mL) drop casted on freshly cleaved mica.

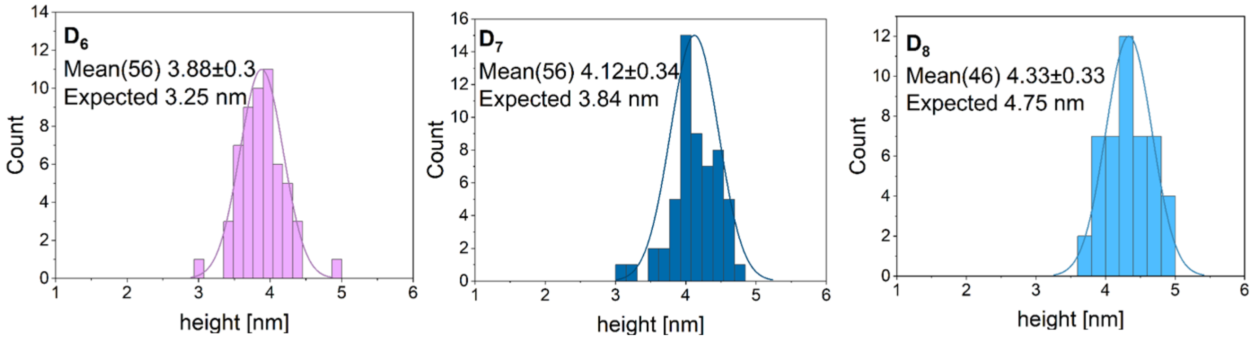

Figure S36 AFM height histograms for single-layer platelets. The height of the platlets was measured manually to avoid artifacts caused by edges or overlays.

4.5 Chirality analysis

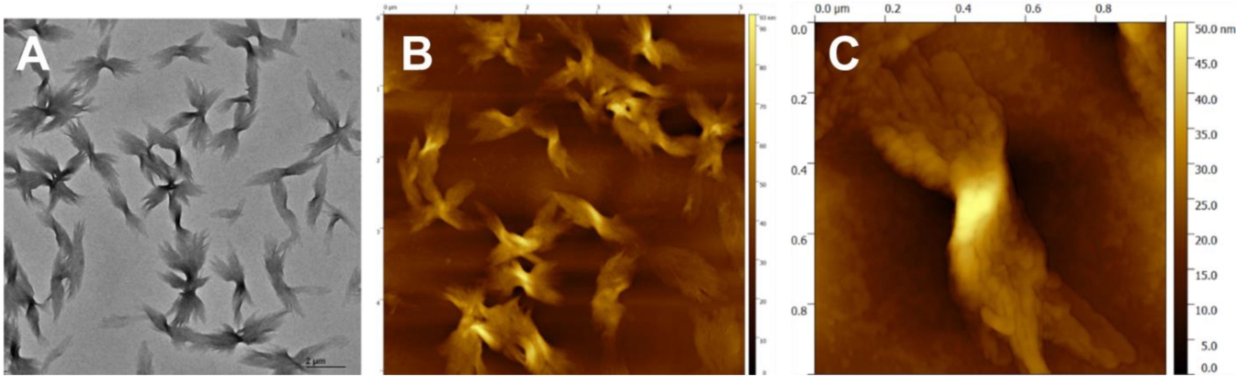

Figure S37 A) Representative TEM image obtained from $\mathbf{D_6}$ aqueous suspension (1 mg/mL). B-C) AFM height image obtained from the same TEM grid.

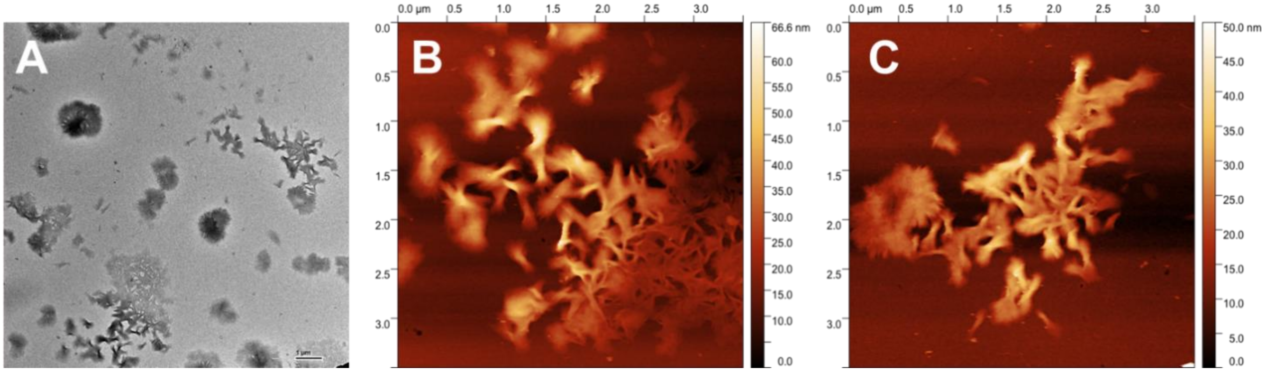

Figure S38 A) Representative TEM image obtained from $\mathbf{LD_6L}$ MeOH suspension. B-C) AFM height image obtained from the same TEM grid.

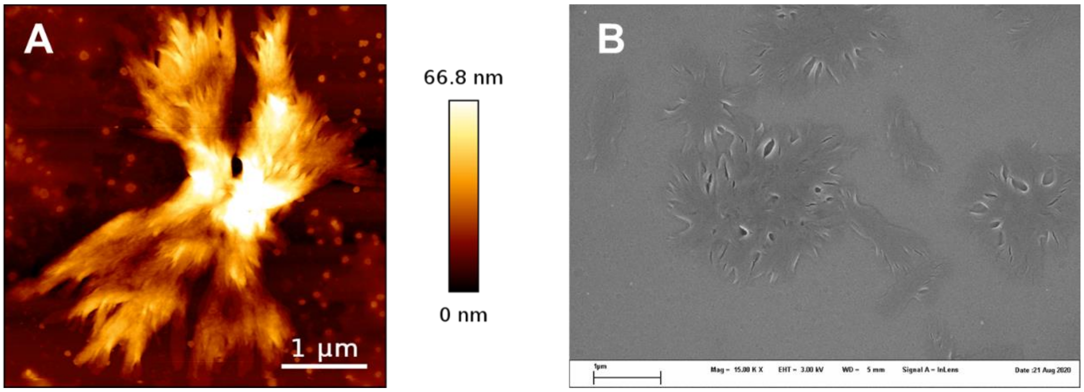

Figure S39 Bundles of cellulose platelets are observed on different surfaces. A) AFM height image obtained from $\mathbf{L_6}$ aqueous suspension (0.1 mg/mL) drop casted on a glow discharged silicon wafer. B) SEM image obtained from $\mathbf{D_6}$ aqueous suspension (1 mg/mL) drop casted on a hydrophilic glass substrate.

5 References

(1) Eller, S.; Collot, M.; Yin, J.; Hahm, H. S.; Seeberger, P. H. Automated Solid-Phase Synthesis of Chondroitin Sulfate Glycosaminoglycans. *Angew. Chem., Int. Ed.* **2013**, *52* (22), 5858–5861. https://doi.org/10.1002/anie.201210132.

(2) Dallabernardina, P.; Schuhmacher, F.; Seeberger, P. H.; Pfrengle, F. Automated Glycan Assembly of Xyloglucan Oligosaccharides. *Org. Biomol. Chem.* **2016**, *14* (1), 309–313. https://doi.org/10.1039/C5OB02226F.

(3) Le Mai Hoang, K.; Pardo-Vargas, A.; Zhu, Y.; Yu, Y.; Loria, M.; Delbianco, M.; Seeberger, P. H. Traceless Photolabile Linker Expedites the Chemical Synthesis of Complex Oligosaccharides by Automated Glycan Assembly. *J. Am. Chem. Soc.* **2019**, *141* (22), 9079–9086. https://doi.org/10.1021/jacs.9b03769.

(4) Gim, S.; Fittolani, G.; Nishiyama, Y.; Seeberger, P. H.; Ogawa, Y.; Delbianco, M. Supramolecular Assembly and Chirality of Synthetic Carbohydrate Materials. *Angew. Chem., Int. Ed.* **2020**, *59* (50), 22577–22583. https://doi.org/10.1002/anie.202008153.

(5) Gude, M.; Ryf, J.; White, P. D. An Accurate Method for the Quantitation of Fmoc-Derivatized Solid Phase Supports. *Lett. Pept. Sci.* **2002**, *9* (4), 203–206. https://doi.org/doi.org/10.1023/A:1024148619149.

(6) Yu, Y.; Tyrikos-Ergas, T.; Zhu, Y.; Fittolani, G.; Bordoni, V.; Singhal, A.; Fair, R. J.; Grafmüller, A.; Seeberger, P. H.; Delbianco, M. Systematic Hydrogen-Bond Manipulations To Establish Polysaccharide Structure-Property Correlations. *Angew. Chem., Int. Ed.* **2019**, *58* (37), 13127–13132. https://doi.org/10.1002/anie.201906577.

(7) Guberman, M.; Bräutigam, M.; Seeberger, P. H. Automated Glycan Assembly of Lewis Type I and II Oligosaccharide Antigens. *Chem. Sci.* **2019**, *10* (21), 5634–5640. https://doi.org/10.1039/C9SC00768G.

(8) Hurevich, M.; Kandasamy, J.; Ponnappa, B. M.; Collot, M.; Kopetzki, D.; McQuade, D. T.; Seeberger, P. H. Continuous Photochemical Cleavage of Linkers for Solid-Phase Synthesis. *Org. Lett.* **2014**, *16* (6), 1794–1797. https://doi.org/10.1021/ol500530q.

(9) Ohara, K.; Lin, C.-C.; Yang, P.-J.; Hung, W.-T.; Yang, W.-B.; Cheng, T.-J. R.; Fang, J.-M.; Wong, C.-H. Synthesis and Bioactivity of β-(1→4)-Linked Oligomannoses and Partially Acetylated Derivatives. *J. Org. Chem.* **2013**, *78* (13), 6390–6411. https://doi.org/10.1021/jo4005266.

(10) Ogawa, Y.; Putaux, J.-L. Transmission Electron Microscopy of Cellulose. Part 2: Technical and Practical Aspects. *Cellulose* **2019**, *26* (1), 17–34. https://doi.org/10.1007/s10570-018-2075-x.
